# Towards a proteomic plasma biomarker panel for diagnosing vasculitis remission

**DOI:** 10.1038/s41467-026-75755-6

**Published:** 2026-07-21

**Authors:** Uwe Jerke, Marieluise Kirchner, Theda UP Bartolomaeus, Lovis Kling, Vojtech Kratky, Zdenka Hruskova, Vladimir Tesar, Kai-Uwe Eckardt, Adrian Schreiber, Sofia Forslund, Philipp Mertins, Ralph Kettritz

**Affiliations:** 1 Experimental and Clinical Research Center (ECRC) and Max Delbrück Center for Molecular Medicine (MDC) in the Helmholtz Association and Charité, Berlin, Germany; 2 Core Unit Proteomics, Berlin Institute of Health (BIH), Charité and MDC, Berlin, Germany; 3 Centre for Ecological and Evolutionary Synthesis (CEES), Department of Biosciences, University of Oslo, Oslo, Norway; 4 Department of Nephrology and Medical Intensive Care, Charité, Berlin, Germany; 5 Department of Nephrology, General University Hospital and First Faculty of Medicine, Charles University in Prague, Prague, Czech Republic

**Keywords:** Autoimmunity, Mass spectrometry, Machine learning

## Abstract

Active anti-neutrophil cytoplasmic autoantibody (ANCA)-associated vasculitis (AAV) requires intensive immunosuppressive therapy, but continued treatment beyond remission risks serious harm. Because reliable biomarkers of remission are lacking, clinicians often prolong cost-intensive and toxic therapy unnecessarily. Here, we explore the plasma proteome to identify reliable biomarkers of disease remission in patients with AAV, adjusting for patient characteristics and clinical variables. Applying a two-tiered proteomics strategy that combines global discovery with targeted validation, we identify a protein signature of remission. We then implement the resulting 7-protein panel in a targeted mass spectrometry-based assay and confirm its diagnostic performance in an independent patient cohort. The panel consistently outperforms routine markers such as C-reactive protein and ANCA titer. Our findings suggest that this 7-protein panel provides a starting point for developing a clinical tool to support decision-making, with the potential to reduce treatment-related burden, mitigate toxicity, and lower healthcare costs. More broadly, our confounder-controlled proteomics approach provides a scalable blueprint for biomarker discovery in complex inflammatory diseases.

## Abstract (web summary)

ANCA-associated vasculitis requires immunosuppression, but reliable biomarkers to confirm remission are lacking. Here, the authors show that a proteomics-machine learning approach identify a biomarker panel that accurately discriminated remission from active autoimmune vasculitis.

## Introduction

Anti-neutrophil cytoplasmic autoantibody (ANCA)-associated vasculitis (AAV) comprises a group of rare life-threatening systemic autoimmune diseases that damages small blood vessels, most frequently in the lungs and kidneys^1^. The principal clinical disease entities of ANCA-AAV are granulomatosis with polyangiitis (GPA) and microscopic polyangiitis (MPA). AAV arises from a breakdown of immune tolerance to proteinase 3 (PR3) or myeloperoxidase (MPO), resulting in the production of PR3- or MPO-specific ANCA. These autoantibodies, in concert with dysregulated innate and adaptive immune cells, drive a pathogenic cascade that culminates in necrotizing inflammation of small vessels^2,3^.

Standard therapies for AAV consist of glucocorticoids, cytotoxic agents, B-cell-depleting antibodies, and C5a complement inhibitors, all of which effectively suppress autoimmune inflammation and induce remission^4–6^. However, immunosuppression comes at a high cost: infections remain the leading cause of morbidity and mortality and contribute substantially to both direct and indirect healthcare burden^7–13^. With an aging patient population and the rising prevalence of chronic inflammatory disorders, the need for safer, more precise diagnostic approaches to inform treatment is increasingly urgent^14,15^.

The standard AAV treatment is not individualized and involves an initial intensive induction phase of 3–6 months to achieve remission, followed by a de-escalated remission maintenance phase of at least 18 months^5,6^. In clinical practice, treatment durations are often extended particularly in patients with a history of relapse or elevated autoimmune and inflammatory markers such as ANCA titers^16–21^ and C-reactive protein (CRP)^22,23^. However, ANCA and CRP may remain elevated during remission or unchanged during relapse, limiting their clinical utility^16,24^. Thus, an unmet need for dependable, objective biomarkers of remission exists. Individualized treatment decisions, including when to taper, discontinue, or reinitiate immunosuppression, depend on reliable disease activity assessments together with additional variables, including relapse probability^25^. Assessment of AAV disease activity in clinical practice is currently assessed using the Birmingham Vasculitis Activity Score version 3 (BVAS v.3), a 56-item composite tool^26^. Although the BVAS performs well in clinical trials, its application in routine care is limited by complexity, subjectivity, and dependence on clinician interpretation. In addition, blood- and urine-based biomarkers have been proposed, yet a reliable, non-invasive molecular marker or marker panel of vasculitis remission remain an unmet need^27^.

Advances in plasma proteomics have opened new opportunities for immune monitoring in systemic disease^28,29^. High-resolution technologies such as liquid chromatography–tandem mass spectrometry (LC-MS/MS) and affinity-based multiplex proteomics such as Olink’s proximity extension assay (PEA) now enable sensitive and multiplexed protein quantification directly from plasma^30^. In particular, targeted approaches such as parallel reaction monitoring (PRM-MS) ensure precise and reproducible detection of candidate biomarkers, supporting translational implementation^31^. Proteomics-based multiprotein panels have already demonstrated diagnostic and prognostic value in complex conditions, including outcome prognosis in patients with COVID-19^32^ and disease monitoring in multiple sclerosis^33^.

Conceivably, the plasma proteome of AAV patients encodes remission-specific molecular patterns that can be uncovered using confounder-aware analysis. We applied a two-tiered proteomics strategy combining untargeted LC-MS/MS-based discovery with targeted PRM-MS validation in independent, clinically well-annotated patient cohorts. Integrating machine learning (ML) with stringent adjustment for confounders, we identified a 7-protein panel that accurately discriminates remission from active disease.

## Results

### Study design and analytical strategy

**Fig. 1:**
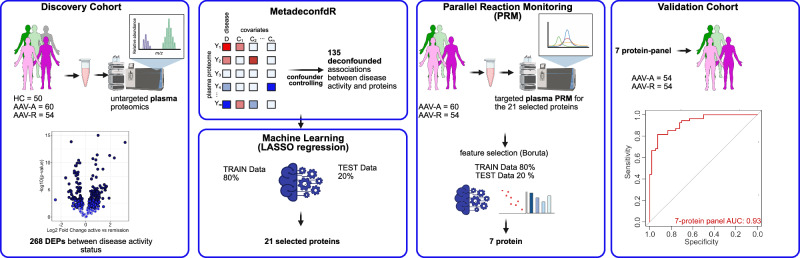
Study design. Step 1: Global plasma proteomics revealed 268 differentially expressed proteins (DEPs) in the discovery cohort of 50 healthy controls (HC), 60 active and 54 remission AAV patients. Step 2: Confounder-aware feature selection using MetaDeconfoundR accounting for CRP, eGFR, hemoglobin, hematocrit, platelets, ANCA titer, and other recorded clinical variables identified 135 proteins with disease associations not statistically reducible to any measured covariate. LASSO-penalized logistic regression revealed 21 candidate proteins. Step 3: Verification by PRM-based targeted MS and additional Boruta feature reduction identified a 7-protein signature panel for vasculitis remission diagnosis. Step 4: Integrative machine learning and application of the trained Generalized Linear Model (GLM) in the independent validation cohort (54 active and 54 remission AAV patients). (Figure was created in BioRender, Kling, L. (2026), https://BioRender.com/p9gk86j).

We conducted a multi-stage proteomics analysis to identify and validate a plasma protein signature of remission in ANCA-AAV. In an initial discovery cohort, we profiled the global plasma proteome using LC-MS/MS and applied confounder-aware statistical modeling combined with ML to isolate proteins that distinguish active disease from remission. We then developed a targeted PRM-MS assay for these candidates and validated the resulting diagnostic panel in an independent validation cohort (Fig. 1).

### Global plasma proteome and disease activity

To assess the plasma proteome profiles in a discovery cohort, we analyzed 50 HC (healthy controls) and 113 AAV patients (59 with active disease and 54 in remission, comprising both PR3-ANCA and MPO-ANCA subgroups, see Supplementary Table 1). Using data-independent acquisition mode (DIA)-based LC-MS/MS, we robustly quantified 605 proteins out of 970 initially identified (Method section, Supplementary Data 1).

**Fig. 2:**
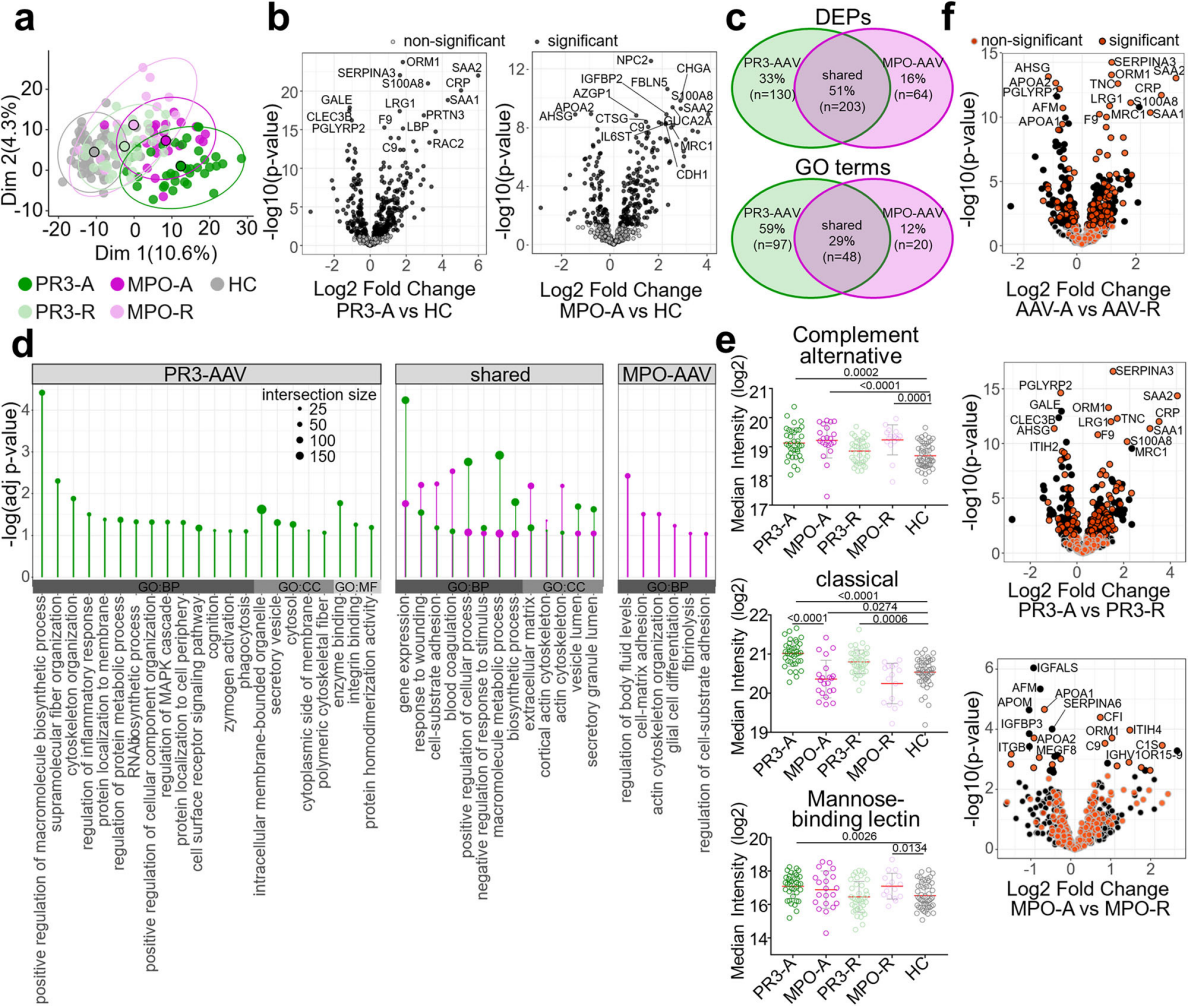
Global plasma proteomics and machine learning algorithms identify disease status specific signature. **a** Principal Component Analysis (PCA) of plasma protein intensity values from five groups: healthy controls (HC, gray, *n* = 50), active MPO-ANCA vasculitis (MPO-A, dark purple, *n* = 22), MPO remission (MPO-R, light purple, *n* = 16), active PR3-ANCA vasculitis (PR3-A, dark green, *n* = 38), and PR3 remission (PR3-R, light green, *n* = 38). Each point represents single individual. The first two principal components (Dim1 and Dim2) explain 10.6% and 4.3% of the total variance, respectively. Ellipses represent 95% confidence intervals, and group centroids are indicated. Group separation was significant (two-sided PERMANOVA, Canberra distance, *p* = 0.001***). **b** Volcano plots comparing active disease and HC, for PR3-A patients (left, *n* = 36 active, *n* = 50 HC) and for MPO-A patients (right, *n* = 22 active, *n* = 50 HC). The x-axis shows log₂ fold changes, and the y-axis indicates −log₁₀ p-value from two-sided Welch’s t-test with FDR correction. Significantly differentially expressed proteins (DEPs) are marked in black, non-significant proteins in gray. Top differential proteins are labeled with gene names. **c** Overlap of DEPs (top) and GO terms (bottom) between PR3-AAV (green) and MPO-AAV (purple). **d** Gene Ontology enrichment analysis of DEPs from PR3-A and MPO-A compared to HC. Lollipop plots show significantly enriched GO terms for PR3-specific (left), MPO-specific (right), and shared (middle) terms. Bar length indicates significance ( − log₁₀ adjusted p-value, one-sided Fisher’s Exact test with FDR correction) and circle size indicates the number of annotated genes. Bar colors indicate GO category: biological process (black), cellular compartment (gray), and molecular function (light gray). **e** Scatter dot plots showing median log₂ LFQ intensity of indicated protein signatures across clinical groups (*n* = 50 HC, *n* = 36 PR3-A, *n* = 22 MPO-A, *n* = 39 PR3-R, *n* = 16 MPO-R, each dot represents one patient). Mean and SD are presented, two-tailed Mann–Whitney U test was performed. The panel provides precise *p*-values for the comparisons illustrated. **f** Volcano plots comparing active and remission disease state for all AAV patients (top, *n* = 59 active, *n* = 54 remission), for PR3-AAV patients (middle, *n* = 36 active, *n* = 38 remission), and for MPO-AAV patients (bottom, *n* = 22 active, *n* = 16 remission). The x-axis shows log₂ fold changes, the y-axis indicates -log_10_ p-value from two-sided Welch’s t-test, FDR corrected. Significantly differentially expressed proteins (DEPs) are marked in black, non-significant in gray, and red highlights DEPs associated with inflammation. Top differential proteins are labeled with gene names.

Unsupervised analysis confirmed that AAV disease state has a strong imprint on the plasma proteome. Principal Component Analysis (PCA) revealed a clear separation of active AAV samples from both HC and remission AAV, indicating disease-associated proteomics signatures (Fig. 2a). Pairwise correlation of group-wise median intensities confirmed these findings and revealed overlapping profiles between active PR3- and active MPO-AAV subgroup (Supplementary Fig. 1a), suggesting a largely shared signature of active disease. PCA and permutational multivariate analysis of variance (PERMANOVA) of global plasma proteome showed that freezer time, MS plate, and MS run order each accounted for ≤1.2% of the total variance, whereas disease status explained 34.2% (*p* = 0.001), indicating that these technical covariates did not materially affect the dominant proteomic structure (Supplementary Fig. 1b–f).

To characterize biological processes associated with active AAV, we examined differentially abundant proteins that were either shared between or specific to active PR3- and MPO-AAV, followed by Gene Ontology (GO) enrichment analysis (Fig. 2b–d). Comparison with HC showed that 51% of differential proteins and 29% of enriched GO terms overlapped between active PR3- and MPO-AAV (Fig. 2c and Supplementary Data 2).

Shared GO biological processes included coagulation, adhesion, wound response, and gene expression, shared cellular components encompassed extracellular matrix, cytoskeleton, and granule structures (Fig. 2d). These findings indicate coordinated regulation of inflammation, tissue remodeling, fibrosis, and structural adaptation, consistent with known AAV pathology. Active PR3-AAV patients showed stronger inflammatory signatures than MPO-AAV, in line with higher CRP concentrations, leukocyte counts, and neutrophil numbers (Supplementary Table 1).

Selected GO terms linked to inflammatory and pathophysiological processes were further analyzed using representative proteins from the global proteome (Supplementary Data 1). Median log₂ intensity values confirmed a robust inflammatory response in active AAV, more pronounced in PR3-AAV, that did not normalize in remission despite a BVAS of zero (Supplementary Fig. 1g). Proteins associated with blood coagulation and venous thromboembolism (VTE) were strongly regulated in active AAV consistent with reported thromboembolic events during active disease^34^ (Supplementary Fig. 1h, i). Organ fibrosis signatures were elevated in both AAV serotypes and remained increased in remission (Supplementary Fig. 1j). Analysis of complement pathways that provide mechanistic and clinical disease features^35,36^ showed activation of the alternative pathway in both active PR3- and MPO-AAV, whereas classical and mannose-binding lectin (MBL) pathway activation was restricted to active PR3-AAV (Fig. 2e).

We next compared patients with active AAV and those in remission to identify proteins that distinguish these disease activity states. Differential expression analysis identified 268 proteins significantly altered between active and remission AAV at a false discovery rate (FDR) of 5% (Fig. 2f). Subgroup-stratified analysis yielded 260 differentially expressed proteins (DEPs) for PR3-AAV and 31 for MPO-AAV (Fig. 2f). Marker proteins of immune and inflammation processes, including acute phase response, regulation of cytokine production, and humoral immunity, were found among the most differential proteins, exemplified by CRP, S100A8, TNC, TIMP1, and ORM1.

### Confounder-aware feature selection

To identify proteins associated with AAV disease activity independently of systemic inflammation, renal function, and additional co-variates, we applied MetaDeconfoundR to test whether protein–disease activity associations were reducible to clinical covariates, including CRP, eGFR, ANCA, hemoglobin, hematocrit, and platelets. Of 605 quantified proteins, 135 showed disease-associated signal non-reducible to any of the measured covariates (Supplementary Data 3, Supplementary Fig. 2a, b).

Supervised feature selection using LASSO on the 80/20 discovery split reduced this protein set to 21 candidates (Supplementary Table 2 and 3), which showed only modest pairwise correlations, except for moderate co-expression among three keratins (Supplementary Fig. 2c). Inspection of the MetaDeconfoundR output for these 21 proteins confirmed covariate associations with CRP, eGFR, hemoglobin, hematocrit, platelets, and ANCA titer (Supplementary Fig. 2d), and all 21 proteins retained covariate-corrected disease activity associations confirming that their relationship with disease activity was not statistically reducible to any of these clinical variables (Supplementary Data 3). The 21-protein panel discriminates the disease activity status in the test set with an AUC of 0.92 (95% CI 0.81–1.00) (Supplementary Fig. 2e). For several proteins, such as AHSG and COMP, the correlation with CRP could reflect their shared involvement in disease activity processes rather than serving as simple CRP surrogates. LRG1 and CLEC3B showed residual associations with CRP consistent with their known roles in the acute-phase response, but their disease associations remained independent of CRP after covariate correction.

### Targeted biomarker validation and feature reduction

**Fig. 3:**
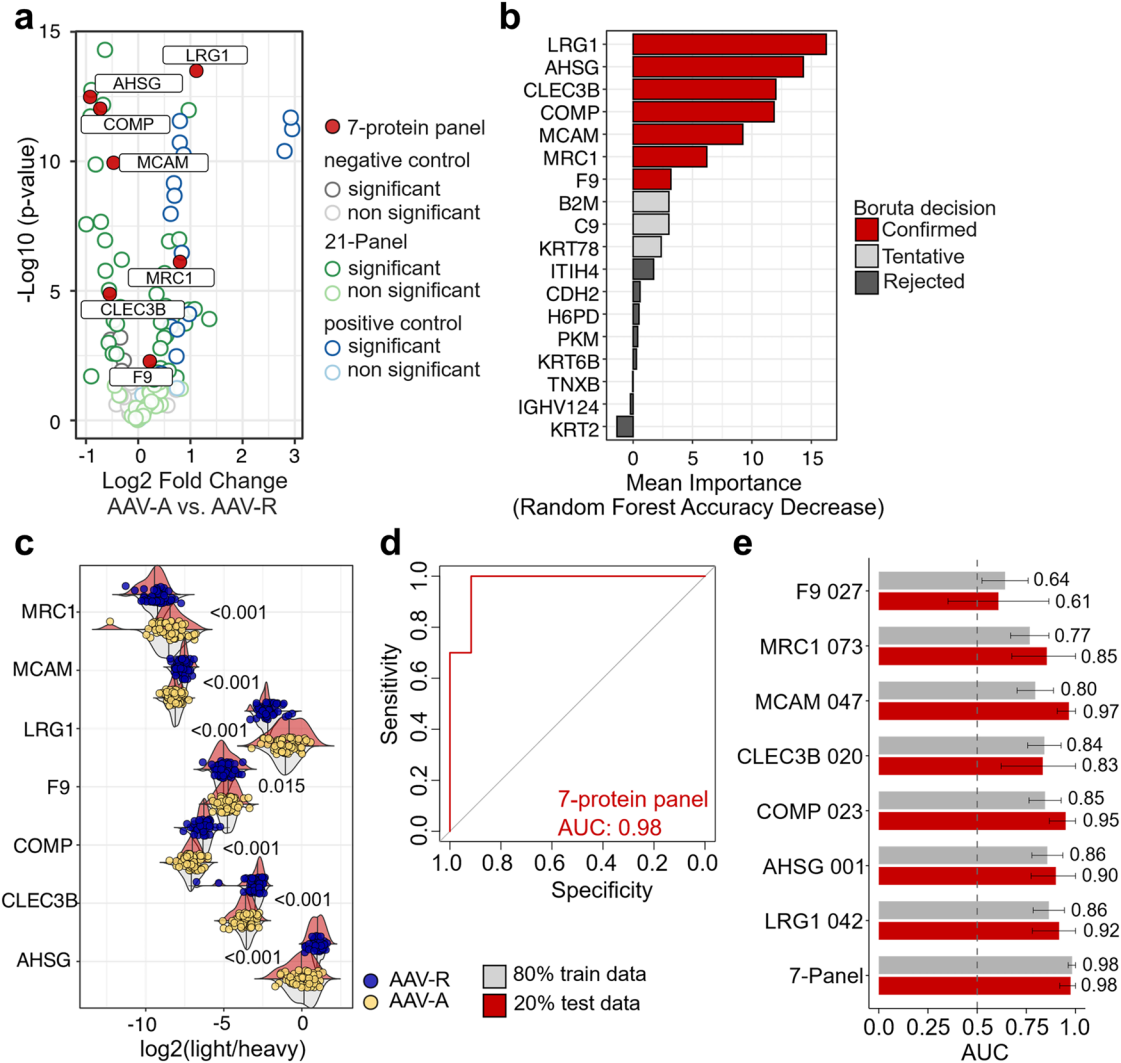
PRM validation of biomarker candidates and Boruta feature reduction yields a 7-protein panel. **a** Volcano plot illustrating differential peptide abundance measured by targeted PRM-MS. Peptides from the final 7-peptide panel are shown as filled dark red circles and labeled with gene name. All other peptides are shown as open circles with colored outlines: green outlines indicate the 21 LASSO-selected candidate proteins, gray outlines indicate technical loading controls peptides (negative controls), and blue outlines represent peptides previously reported as differentially expressed in AAV (positive controls). The x-axis shows log₂ fold change (AAV-A vs. AAV-R), and the y-axis represents statistical significance as –log_10_ (two-sided Welch’s t-test, BH-FDR). Peptides with FDR < 0.05 are shown in full color, non-significant peptides (FDR ≥ 0.05) are shown in lighter shades. **b** Feature importance ranking following Boruta feature selection. Confirmed, tentative, and rejected features are shown in dark red, gray, and green, respectively. **c** Violin plots of log_2_(light/heavy) ratios for the 7-protein panel (AHSG, CLEC3B, COMP, F9, LRG1, MCAM, MRC1) in active and remission. AAV patients are stratified by discovery split (80% training set (gray, *n* = 88) and 20% held-out test set (red, *n* = 22)). Two-tailed Mann-Whitney U test was applied. The precise p-values for the comparison between active and remission AAV are provided alongside the violin plots. **d** ROC curve from GLM trained on the 80% training set and applied once to the held-out 20% test set, with AUC indicated. **e** Comparison of ROC AUC values with 95% CI for the combined 7-protein panel versus each individual panel protein, evaluated in both the 80% training (gray, *n* = 88) and 20% test (red, *n* = 22) partitions of the discovery cohort.

We next developed a targeted mass spectrometry assay to validate the 21 candidates from the discovery phase. This targeted approach improves the sensitivity, reduces missing values, and enables precise protein quantification. For each protein, we selected four proteotypic tryptic peptides and their corresponding synthetic heavy-labeled internal spike-in standards (SpikeTide_L) (Supplementary Data 4a). The resulting PRM-MS data corroborated the global proteome findings: 53 peptides showed significant differential abundance between active and remission AAV, with 18 of 21 proteins supported by at least two independent peptides (Fig. 3a, Supplementary Data 5, Supplementary Table 4).

To identify a minimal reproducible diagnostic protein set, we employed Boruta feature selection to the same 80/20 training–test split. Boruta identified seven proteins as the most informative for distinguishing disease states forming the final diagnostic 7-protein panel: alpha-2-HS glycoprotein (AHSG), tetranectin (CLEC3B), cartilage oligomeric matrix protein (COMP), coagulation factor IX (F9), leucine-rich alpha-2-glycoprotein 1 (LRG1), cell surface glycoprotein MUC18 (MCAM), and macrophage mannose receptor 1 (MRC1) (Fig. 3b). Log₂(light/heavy) peptide ratio for these seven proteins showed reproducible distribution across the training and test and revealed statistically significant abundance differences between active and remission samples (Fig. 3c, Supplementary Table 5).

Next, a 7-protein logistic regression model (GLM) was trained on the same discovery 80/20 data split used for feature selection, with all modeling confined to the training data and the held-out 20% test set used solely for evaluation. The model achieved an AUC of 0.98 (95% CI 0.92–1.00) with a Brier score of 0.07 (Fig. 3d). Permutation testing confirmed no evidence of overfitting in the trained model, and label-permutation testing that the result was not attributable to chance (*p* < 0.001, Supplementary Tables 6 and 7). Calibration in this small internal test set showed near-complete separation (Supplementary Fig. 3a). Finally, a Firth-penalized sensitivity analysis yielded an identical AUC (ΔAUC = 0.00), confirming that discrimination reflected a genuine signal rather than a separation artifact (Supplementary Fig. 3b). The 7-protein panel outperformed each individual marker in the held-out test set (Fig. 3e, Supplementary Table 8), supporting the transferability of the 7-protein signature to the targeted PRM platform.

### Biomarker quantification and validation

**Fig. 4:**
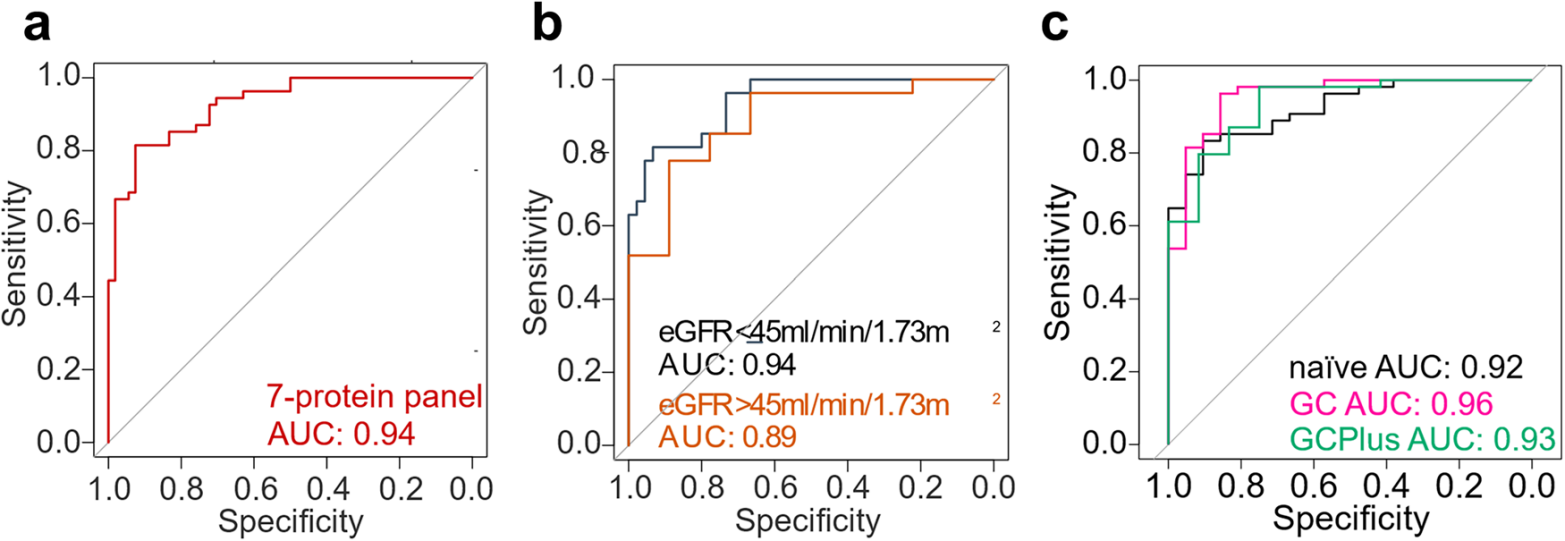
Validation of the 7- protein panel in an independent cohort. PRM-MS measurements with TQL peptides were performed and the GLM algorithm was trained in the discovery (*n* = 104) and tested in an independent validation cohort (*n* = 108). **a** The ROC curve shows excellent discrimination between active and remission AAV in the validation cohort, with (**b**) showing the 7-protein panel performance in remission AAV patients with eGFR > 45 ml/min/1.73m^2^ (*n* = 27) and ≤ 45 ml/min/1.73m^2^ (*n* = 27), with no significant difference between strata (two-sided DeLong test, n.s.) **c** Subgroup analysis of panel performance by treatment group. Active AAV patients were stratified into treatment-naïve (*n* = 21), glucocorticoid only (GC, *n* = 21), and glucocorticoid plus immunosuppressive therapy (GC + IS, *n* = 12) groups. Similar ROC curves were established for the three treatment groups, excluding significant treatment effects on the panel performance (n.s. in two-sided DeLong-Test).

To enhance clinical applicability, we optimized the 7-protein assay by including highly purified (isotopical purity > 99%), absolute-quantified heavy peptide standards (SpikeTides Tagged Quantified Labeled, TQL) for each target protein (Supplementary Data 4b). This refinement enabled precise quantification and reduced runtime from 110 to 30 min per sample. The resulting quantitative log_2_ ratio values (Supplementary Data 6a) showed excellent concordance with global proteomics data and correlated excellently with the corresponding data from global proteome measurements, which are based on at least five proteotypic peptides per protein and no less than 85% coverage within the cohort (Supplementary Fig. 3c and Supplementary Data 1). Using the quantitative output from the PRM analyses, we retrained the GLM on the full discovery cohort and evaluated its performance in an independent validation cohort of 108 AAV patients using the same platform and pipeline (Supplementary Table 9 for clinical characteristics, Supplementary Data 6b for log_2_ ratio values from PRM measurements). The 7-protein panel achieved an AUC of 0.94 (95% CI 0.90–0.98) for distinguishing active disease from remission in the external validation cohort, demonstrating good discriminative performance (Fig. 4a). Label-permutation confirmed that this discrimination exceeds chance expectation (*p* < 0.001). Thus, the 7-protein panel performed robustly in the external cohort, validating our protein selection. The calibration impairment observed with the discovery GLM (Supplementary Table 10) was attributable to near complete separation in the discovery training set. This known effect of logistic regression indicates that the calibration issue reflects model estimation instability rather than poor predictive signal in the protein panel composition. A parallel Ridge-penalized model confirmed this statement by showing that this discrimination was fully preserved and calibration substantially improved in the external cohort (Supplementary Fig. 4a, Supplementary Table 11). Performance was consistent across kidney function strata (AUC 0.94 (95% CI 0.89–0.99) for eGFR ≤ 45 ml/min/1.73m^2^, AUC 0.89 (95% CI 0.77–1.00) for eGFR > 45 ml/min/1.73m^2^) and induction treatment subgroups (immunosuppression-naïve: AUC 0.92 (95% CI 0.86–0.98), glucocorticoid monotherapy: AUC 0.96 (95% CI 0.91–1.00), glucocorticoid plus cyclophosphamide or rituximab: AUC 0.93 (95% CI 0.86–1.00)) without statistically significant differences between any subgroup comparison (Fig. 4b, c, Supplementary Tables 12–17).

A sensitivity analysis using Ridge penalization improved the calibration characteristics while preserving discriminative performance (AUC 0.95 (95% CI 0.91–0.98)), confirming that the protein-based signal is robust to the choice of estimation method (Supplementary Fig. 4b).

### 7-protein panel performance in the combined patient cohorts

**Fig. 5:**
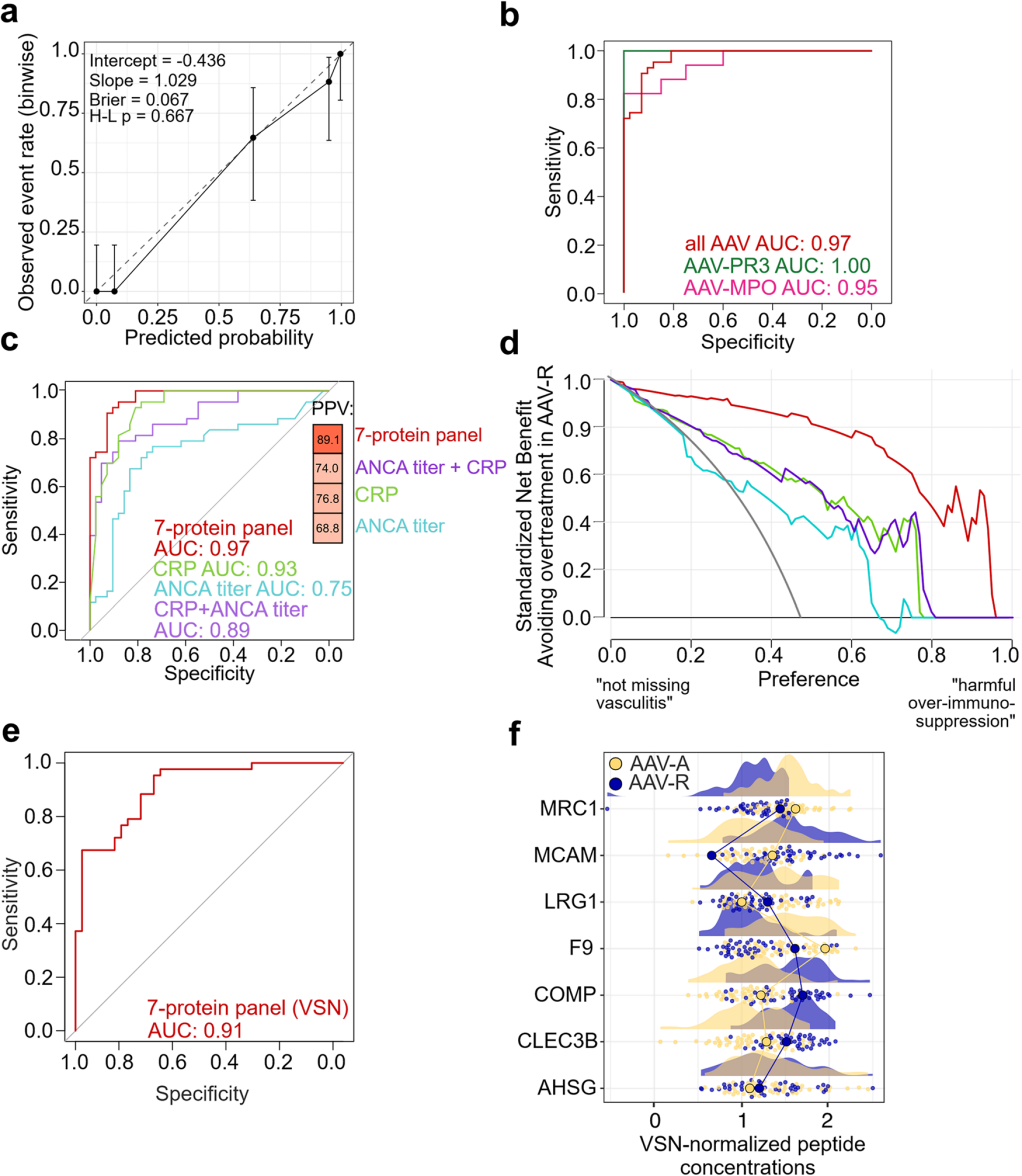
Performance of the diagnostic 7-protein panel in the combined cohort. **a** Calibration plot showing means of predicted versus observed remission rates binned across predicted probability quintiles. Error bars represent 95% confidence intervals from 1000 bootstrap replicates, and the dashed line indicates perfect calibration. Performance metrics shown include calibration intercept, slope, Brier score, and Hosmer–Lemeshow goodness-of-fit. **b** ROC curves of the 7-protein panel in the 40% test cohort stratified by ANCA serotype: all AAV patients (brown, *n* = 85), PR3-AAV (green, *n* = 48), and MPO-AAV (purple, *n* = 37). **c** ROC curves of the 7-protein panel (brown), ANCA titer (turquoise), CRP (green), and ANCA + CRP combined (violet) in the 40% test cohort (*n* = 85). Heat map of corresponding positive predictive values (PPVs) is depicted across probability thresholds. **d** Decision curve analysis (DCA) of the 7-protein panel prediction model (brown) compared to CRP (green), ANCA titer (turquoise), CRP + ANCA combined (violet). The DCA includes a hypothetical scenario where immunosuppression is stopped in all patients (intervene all, oblique gray line) and the opposite hypothetical scenario where all patients continue immunosuppression (intervene none, horizontal gray line) regardless of the diagnostic test result. The x-axis plots the preference (stopping treatment), ranging from “not missing vasculitis activity” (left) to “harmful over-immunosuppression” (right). The y-axis shows the standardized net benefit (avoidance of unnecessary immunosuppression in remission AAV patients) of using the predictive test as the percentage of true positive remissions within the intervention group. **e** ROC curve for a concentration-based implementation of the panel, in which TQL-based PRM-MS signal intensities were converted to VSN-normalized absolute peptide concentrations. **f** Density plots of plasma protein concentrations of all patients from the test data set (*n* = 85) for an active (blue) and a remission (yellow) AAV patient are highlighted and connected.

To further assess generalizability and clinical relevance, we introduced deliberate geographic and technical heterogeneity by combining both independent cohorts from different countries. The discovery and validation cohorts also differed in remission duration and maintenance therapy regimens (Supplementary Table 1 and 9). We performed a pre-specified 60/40 stratified split with larger patient numbers after excluding differential panel performance between cohorts (interaction *p* = 0.748). The GLM showed good calibration, permutation testing confirmed no evidence of overfitting in the retrained model, and label-permutation testing shows that the result was not attributable to chance (*p* < 0.001, Supplementary Table 18, Fig. 5a). Log-odds estimates from the final GLM revealed that MRC1, LRG1, and F9 are strongly associated with active AAV, whereas MCAM, COMP, CLEC3B, and AHSG contribute positively to the remission probability (Supplementary Fig. 5b). Patient-level analysis of TQL-normalized peptide ratios showed concordant predicted ratio values and close agreement with clinician-assessed BVAS scores, with closely aligned group-level medians (Supplementary Table 19, Supplementary Fig. 5b).

In the held-out Test40 set, the panel achieved an AUC of 0.97 (95% CI 0.95–1.00) and panel discrimination was maintained across ANCA serotype subgroups: AUC 0.95 (95% CI 0.89–1.00) in MPO-ANCA patients and AUC 1.00 in PR3-ANCA patients, though the latter should be interpreted cautiously given the small subgroup size (Fig. 5b, Supplementary Table 20). We next tested whether the panel outperformed CRP and ANCA. The AUC of the 7-protein panel was higher compared to ANCA titer (AUC 0.75 (95% CI 0.64–0.86), *p* < 0.001), CRP (AUC 0.93 (95% CI 0.88-0.98), *p* < 0.05), and their combination (AUC 0.89 (95% CI 0.83–0.96), *p* < 0.01). Moreover, the 7-protein panel generated fewer false positives compared to CRP (5 vs. 13, specificity 88.1% vs. 69.0%). Adding CRP, ANCA titer, or both to the 7-protein panel did not improve discrimination (Fig. 5c, Supplementary Tables 21 and 22).

Among patients in BVAS-defined remission, 65.7% remained ANCA-positive and 20.0% had elevated CRP. Thus, 73.3% had at least one of these conventional markers elevated despite clinical remission. The 7-protein panel discrimination was maintained in ANCA-positive remission patients (AUC 0.97 (95% CI 0.95–1.00)), in remission patients with elevated CRP ( > 5 mg/L) (AUC 0.97 (95% CI 0.92–1.00)), and in remission patients with both positive ANCA and elevated CRP (AUC 0.97 (95% CI 0.89–1.00)) (Supplementary Fig. 5c, Supplementary Table 23). In addition, the 7-protein panel showed a higher positive likelihood ratio (LR + 8.0 (95% CI 4.24–33.33)) compared to CRP (LR + 3.3 (95% CI 2.21–5.57)), suggesting improved identification of remission (Supplementary Table 23). These findings support the notion that the 7-protein panel reflects the AAV disease activity status where conventional biomarkers are ambiguous.

To evaluate the potential clinical impact of the panel, we conducted decision curve analysis (DCA)^37^ on the held-out Test40 set of the combined cohort with 85 patients and 43 remission events to simulate its use in supporting immunosuppression tapering. Across a broad range of threshold preferences, the 7-protein model demonstrated superior net benefit compared to CRP, ANCA, or their combination indicating its utility in avoiding unnecessary treatment in remission patients (Fig. 5d, Supplementary Fig. 5d and e, Supplementary Tables 24–28).

We further explored whether the 7-protein remission score predicted flare within 24 months in the combined cohort (*n* = 96, 15 flares [16%]). The protein score was not associated with time to flare (Cox per 0.1 increase: HR 0.92 (95% CI 0.76–1.11), *p* = 0.49). Discrimination for 24-month flare was poor (AUC 0.44 (95% CI 0.29–0.60)) (Supplementary Table 29).

For prospective practical implementation, we converted the TQL-based PRM-MS signal intensities into absolute plasma concentrations for each of the seven proteins (Method section, Supplementary Data 6c). After applying variance stabilization normalization (VSN) to account for differing concentration scales, diagnostic performance remained high (AUC 0.91 (95% CI 0.85–0.97)), PPV 78.7% (95% CI 64.3–89.3)) (Fig. 5e, Supplementary Fig. 6a and b, Supplementary Tables 30 and 31), even though individual protein values exhibited some signal overlap between active and remission states (Fig. 5f).

## Discussion

We report the development of an independently validated, clinically scalable, and metadata-aware plasma proteomic signature capable of distinguishing remission from active disease in ANCA-associated vasculitis (AAV). Accurate identification of remission is one critical factor for tailoring immunosuppressive therapy in AAV, a disease in which both under- and overtreatment carry significant clinical and economic consequences. Our work introduces a 7-protein panel that achieved excellent diagnostic accuracy across two independent cohorts, with an AUC of 0.97 (95% CI 0.95–1.00) and PPV of 89.1% (95% CI 76.4–96.4). This performance was superior to standard clinical biomarkers such as ANCA titer and CRP and can be obtained in a single analytical LC-MS analysis. We consider the 7-protein panel as an adjunct to cross‑sectional disease activity assessment in known AAV. We report several key innovations. First, using ML guided by confounder-aware modeling, we isolated a non-redundant set of proteins specifically associated with AAV disease activity, independent of systemic inflammation (CRP) or kidney function (eGFR, creatinine). Second, we developed a targeted, high-sensitivity PRM-MS assay with absolute quantification, ensuring clinical translatability. Third, we confirmed diagnostic performance across geographically and clinically heterogeneous cohorts, underscoring the robustness and generalizability of the panel. Our approach is in line with the Transparent Reporting of a multivariable prediction model for Individual Prognosis Or Diagnosis criteria (TRIPOD, see checklist in Supplementary Table 32)^38^. Fourth, our global proteome analysis provides insights into pathways and proteins engaged in active AAV. We describe shared but also distinct protein signatures in PR3- and MPO-AAV patients, thereby providing a rationale for future mechanistic and translational studies.

AAV remains a high-burden disease despite advances in immunosuppressive therapy. Although clinical remission can be achieved in approximately 75% of patients, treatment-related complications including infection, malignancy, and organ damage, remain leading cause of morbidity and mortality^8,11–13^. The economic impact is substantial, driven by prolonged hospitalizations due to infections, immunosuppressive drug use, and frequent monitoring requirements^7,9,10^. A biomarker panel that reliably identifies remission offers a powerful means helping to reduce overtreatment, mitigating toxicity, and ultimately decreasing healthcare costs.

In clinical practice, the BVAS is commonly used to assess disease activity in AAV^26^, supported by crude inflammatory markers such as CRP and ANCA titers, to assess disease activity. However, these tools have limitations: BVAS scoring is time-consuming and partly subjective. It lacks sensitivity for subclinical disease activity and cannot reliably distinguish activity from chronicity. CRP and ANCA titer can yield ambiguous results in the setting of subclinical disease or remission^16,24^. Thus, there is medical need for biomarkers that allocate patients to either active or remission AAV with high accuracy and specificity.

Several promising single plasma biomarkers have been proposed for assessing AAV disease activity, including TIMP-1, CXCL13, MMP3, leucine-rich α2-glycoprotein-2, MMP9, CD93, S100A8, transketolase, and tenascin C^39,40^. While these markers have shown potential in individual studies, none has been widely implemented or externally validated at scale across diverse clinical settings. We observed reduced plasma COMP levels in active AAV patients. Increased COMP levels were reported in osteoarthritis and rheumatoid arthritis and reduced levels recently in Takayasu arteritis. COMP is an extracellular matrix glycoprotein that contributes to endochondral ossification. However, it is found not only in cartilage, synovium, and bone from where it is released during arthritis^41,42^ but also in the vascular wall^43^. Vascular smooth muscle cells (VSMCs) produce and release COMP into the plasma and the vessel wall where it maintains vascular homeostasis. Conceivably, vasculitis causes VSMCs injury and dedifferentiation resulting in decreased plasma concentrations and possibly contributing to vascular injury. Reduced COMP was recently reported in another vasculitis entity, namely in Takayasu’s suggesting shared disease mechanisms^44^. Activity markers, such as CD163, MCP-1 and T cells, were described in the urine of patients but are limited to kidney AAV activity assessment^45–48^. Across two independent cohorts totaling over 200 patients with this rare disease, the panel achieved AUCs of 0.98 (95% CI 0.92–1.00) discovery, 0.94 (95% CI 0.90-0.98) in the validation, and 0.97 (95% CI 0.95–1.00) in the combined cohort - exceeding the performance of ANCA titer and CRP, which remain current clinical standards. By explicitly training the algorithm to ignore variation attributable to classical acute‑phase reactants such as CRP and to kidney function surrogates like eGFR, we show that machine learning can help identify a signature that is truly independent of generic inflammation or kidney function. Although the 80/20 training split was small, findings were corroborated in an independent validation cohort and a 60/40 re-split analysis, calibration was not pre-specified as a primary endpoint.

Our work also highlights the advantages of a mass spectrometry–based multi-protein assay over conventional single-analyte tests, including higher specificity, the ability to multiplex, and antibody-independent detection of isoforms. The PRM-MS approach ensures high reproducibility, absolute quantification, and potential for cross-platform standardization. Notably, the assay runtime was reduced to 30 min per sample, enabling throughput suitable for both clinical labs and prospective cohort studies. With further technical advances, sub-10-min run times are realistic, enabling 300–1200 samples per week on a single instrument. Importantly, the full workflow, including LC-MS/MS profiling, confounder modeling, and PRM-MS quantification, is modular and adaptable, allowing rapid extension to diverse populations. Nonetheless, future studies should include multi-ethnic cohorts to confirm applicability across broader clinical settings.

There are limitations to acknowledge. Unrecorded pre-analytical variables could have affected the plasma proteome. Although biosampling was not strictly harmonized between the discovery and validation cohorts, a stable protein signature was confirmed in both cohorts. Furthermore, remission and active disease states were defined using BVAS, which, while widely accepted, has known subjectivity and limited sensitivity for subclinical activity. Thus, some patients classified as “remission” could have had mild ongoing inflammation below the detection threshold of BVAS. Moreover, due to the limited sample size and heterogeneity of treatments, the possibility of medication affecting biomarker concentrations independently of disease activity cannot be definitively excluded. Our findings suggest accuracy across treatment strata rather than providing evidence of the absence of treatment effects. Another aspect is that AAV patients were categorized according to their serological status. Three MPO-AAV patients in the discovery and two patients in the validation cohort had eosinophilic granulomatosis with polyangiitis (EGPA). Although EGPA and MPO-ANCA–positive microscopic polyangiitis (MPA) share certain pathogenic features, they may also exhibit mechanistic differences. Therefore, the inclusion of EGPA patients could introduce additional heterogeneity. Nevertheless, given the small number of EGPA cases, this heterogeneity is unlikely to obscure the performance of the 7-protein panel in AAV. Spike-in-based PRM-MS offers a standardized, multiplex quantification strategy. However, cross-laboratory validation is required to ensure reproducibility. The absolute quantification with internal standards employed supports clinical translation, while future clinical deployment could also be supported by alternative targeted platforms (e.g. immunoassays or affinity-based multiplex assays) for routine clinical use following analytical validation.

Finally, future studies are needed to identify proteome signatures predicting AAV flares. We are currently performing the prospective PRE-FLARED 2-study that aims to predict flares using several biomarkers, including plasma and urine proteomics (German Clinical Trials Register DRKS00034858). In addition, whether the 7-protein panel helps with diagnosing AAV needs to be explored by comparisons with appropriate disease controls. Other future research fields pertain to the performance of the plasma 7-protein panel in patients with low level disease activity (“grumbling disease”), in AAV patients with co-morbidities causing inflammation, in other inflammatory autoimmune diseases, and the comparison with urine proteomics and kidney histology.

In summary, we report a 7-protein panel for diagnosing remission in AAV, validated across one independent cohort and developed using metadata-aware machine learning and targeted proteomics. This panel offers a promising tool for individualized immunosuppression management and may reduce treatment-related harm and healthcare costs. Future prospective trials should evaluate its utility in guiding therapy de-escalation and improving long-term outcomes in patients with AAV. Whilst the clinical applicability of the biomarker panel has yet to be determined, it can nevertheless be classified as a generalizable proof-of-concept for biomarker discovery in complex immunological diseases. This classification is based on state-of-the-art proteomics, external validation, and decision curve analyses. However, the clinical benefit of this approach for safe therapy de-escalation must be evaluated in prospective studies.

## Methods

### Study design

For the discovery cohort, 50 healthy controls (HC) and 114 patients with anti-neutrophil cytoplasmic antibody (ANCA)-associated vasculitis (AAV) were included in this study. Patients were described previously^49^ and gave informed consent (local Charité Ethics Committee, Charité- Universitätsmedizin Berlin, Germany (EA4/025/18)). The validation cohort from Prague consisted of 108 patients with AAV. Diagnosis was based on criteria from Chapel Hill Consensus Conference^50^. Vasculitis activity was assessed using the Birmingham Vasculitis Activity Score Version 3 (BVAS) for Wegener’s granulomatosis. All patients were ANCA positive, had generalized disease, and were grouped according to ANCA specificity (PR3-ANCA versus MPO-ANCA) and disease activity (active versus remission), whereby remission was defined as BVAS = 0. Characteristics of HC and AAV patients are given in Supplementary Tables 1 and 9.

### Plasma sample collection and processing for proteomic analyses

Blood samples were collected in heparin-containing tubes using standard venipuncture protocols. Plasma was recovered by centrifugation and aliquoted samples were stored at −80 °C until analysis. Freeze and thaw cycles were limited to a maximum of two. Samples were thawed on ice and dispensed into 96-well plates and the complete sample preparation, except for boiling and centrifugation steps, was performed on an Agilent Bravo liquid handling platform. Plasma was diluted 1:10 in water and 30 μL of the dilution were added to 30 μL SDC digestion buffer (2% Sodium deoxycholate (SDC), 20 mM dithiothreitol (DTT), 80 mM chloroacetamide (CAA), 200 mM Tris-HCl pH 8), mixed and boiled for 10 min. After cooling down to room temperature, LysC and trypsin were added in a 1:25 enzyme to protein ratio and digestion was performed at 37 °C over-night. Peptides were acidified with trifluoro-acetic acid (final concentration 1%) and diluted with 1% formic acid. Samples were centrifuged for 10 min at 1200 g and peptide containing supernatant was transferred for desalting step using C18 5 µL cartridges and the vendors protocol (Agilent). Purified peptides were eluted with 100 μL of elution buffer (50% acetonitrile/0.1%formic acid), completely dried using a SpeedVac centrifuge at 45 °C and stored at −20 °C. For mass spectrometric measurements, peptides were suspended in 3% acetonitrile/0.1% formic acid.

### Global proteome measurements

#### Individual plasma samples

Peptides (1 μg) were loaded onto a 20-cm reversed-phase C18 column (1.9 μm Reprosil-Pur C18 beads, Dr. Maisch) and eluted using a High-Performance Liquid Chromatography (HPLC) system (ThermoScientific). A gradient was generated by using a dual-buffer system with buffer A (3% acetonitrile in H_2_O) and buffer B (90% acetonitrile in H_2_O). Peptides were separated from 2% to 60% B in 32 min with a flowrate of 250 nL/min. Samples were measured in a randomized order on an Q Exactive HF-X Orbitrap instrument (Thermo Fisher Scientific), running on data-independent mode (DIA) with variable isolation window sizes as described^51^. A blank run was placed after each analytical run.

#### Plasma library

5 µL peptides of each sample within the cohort were combined and fractionated using high pH reversed phase off-line chromatography (1290 Infinity, Agilent, XBridge C18 4.6 mm × 250 mm column [Waters, 3.5 µm bead size]). The 196 fractions were concatenated into 52 analytical fractions, which were dried and resuspended in 3% acetonitrile/0.1% formic acid for measurement on an Orbitrap HF-X mass spectrometer (Thermo Fisher Scientific, Waltham, MA, USA), using the same LC parameters as described for DIA measurements. MS data were acquired with a Top20 data-dependent MS/MS scan method (topN method). Target values for the full scan MS spectra were 3 × 10^6^ charges in the 300–1800 m/z range, with a maximum injection time of 10 ms and a resolution of 60 K. Fragmentation of precursor ions was performed at a resolution of 15 K with an ion target value of 1 × 10^5^ and a maximum injection time of 22 ms. Dynamic exclusion was set to 20 s.

#### RAW data analyses

The spectral library was created using Spectronaut software (version 14.3) with a human Uniprot database (2021-01) and a universal protein contaminants list. Modifications were set to Carbamidomethyl (C) as fixed and Acetyl (Protein N-term), Deamidation (NQ), and Oxidation (M) as variable modification. Individual samples were analyzed with Spectronaut as well, using standard settings with precursor *q* value filtering (0.01), global normalization and MaxLFQ quantification. Protein lists were exported, log2-transformed and filtered for human protein entries, having at least 50% valid values. Remaining missing values were imputed using down-shift imputation by random draw from the Gaussian distribution with 0.3x standard deviation and downshift of 1.8x standard deviation of the observed values per sample. One patient sample was excluded from further analyses due to low sample quality. From the 970 identified protein groups, 605 passed the quality filtering and were used for statistical analyses.

The processed global proteome data matrix is provided in the Supplementary Information (Supplementary Data 1). The proteomics raw data have been deposited to the ProteomeXchange Consortium via the PRIDE partner repository with the dataset identifier PXD079232.

### PRM analyses

#### Panel 21

Four tryptic peptides per protein, that were unique across the human proteome, showed robust intensity signals in global proteome data and have a sequence length between 7 and 25 amino acids, were selected, and heavy labeled synthetic variants (SpikeTides) for each peptide were purchased (JPT Inc., Berlin, Germany). Heavy spike-in peptides were processed according to vendors instructions and used for PRM assay development, including library generation, retention time and charge state determination, optimization of collision energy and injection time, as well as spike-in concentration. An inclusion list was created containing light and heavy precursor variants of 132 peptides covering 32 proteins, including the 21 candidates (AHSG, LRG1, CLEC3B, MCAM, F9, APMAP, B2M, CDH2, COMP, H6PD, IGHV1-24, KRT6B, KRT7B, C9, HSPA8, MRC1, KRT2, PKM, IGFBP3, TNXB, ITIH4) and several control proteins (APOB, A1BG, PLTP, GPX3, PROZ, FCGBP, TIMP-1, TNC, ANGPT2, CRP, SERPINA1). PRM measurements were performed on a Vanquish Neo liquid chromatography system (Thermo Fisher Scientific) using a 98 min gradient of increasing buffer B (90% acetonitrile in H_2_O) concentration (from 2% to 60%, 250 nl/min flow rate) coupled to an Exploris 480 mass spectrometer (Thermo Fisher Scientific). For analytical measurements, which were performed in randomized order, a full mass spectrum at 45 K resolution (AGC target 300%, 10 ms maximum injection time) was followed by PRM scans at 60 K resolution (standard AGC value, 15 min retention time window, adjusted injection time and collision energy) as triggered by the scheduled inclusion list. For each analytical run 2 μL of the plasma peptide/spike-in mixture (1 μg/100 fmol) were injected. Data analysis was carried out using the Skyline software package^52^. Heavy spike-in signals, as well as the internal library-based fragment ranking were used to manually confirm the peak assignment. The summed peak area of the 3–8 most intense fragment ions was used to quantify both, the light (endogenous) and heavy (SIL) peptides. The abundance of each targeted peptide was calculated as the ratio between the light and heavy peptide (light/heavy ratio), which were used for quantitation. 126 peptide pairs passed all quality criteria and were used for further statistical analyses. Two sample were excluded from further analyses because of low data quality. Additional patients were excluded from all PRM analyses due to recent plasma exchange (*n* = 4), septic constellation (*n* = 2), and high dose steroid treatment (*n* = 4) that exceeded established protocols at sampling.

#### Panel 7

For the PRM analyses of the reduced diagnostic panel (7-protein panel), best performing peptides from Panel 21 assay were selected based on diagnostic relevance (feature importance) and mass spectrometric criteria (best signal intensity, fragmentation efficiency, dotproduct value). Cross-validation with global proteome data supported the robustness of the seven candidates, which were identified with at least five peptides per protein and a minimum cohort coverage of 85% in the discovery data set (Supplementary Data 1). Highly purified quantifiable heavy labeled synthetic variants (SpikeTide TQL) for each peptide were purchased (JPT Inc., Berlin, Germany). These TQL spike-ins were processed according to vendors instructions, including tryptic cleavage of the TQL tag, and used for PRM assay development. Amount of spike-in standard was adjusted for each target peptide, based on MS signal intensity that was pre-determined in quality control analyses of individual peptides, aiming for a total intensity signal of at least 1E06, allowing robust detection, and close to the endogenous (light) level within a range smaller than 100-fold deviation, for precise quantitation. A PRM inclusion list was created containing light and heavy precursor variants of 15 peptides, covering 12 proteins, including the 7 biomarker candidates (AHSG, LRG1, CLEC3B, MCAM, F9, COMP, MRC1) and five control proteins (APOB, A1BG, TIMP-1, TNC, CRP). PRM measurements were performed on a Vanquish Neo liquid chromatography system (Thermo Fisher Scientific) using a 25-min gradient of increasing buffer B (90% acetonitrile in H_2_O) concentration (from 2% to 60%, 250 nL/min flow rate) coupled to an Exploris 480 mass spectrometer (Thermo Fisher Scientific). Parameters for analytical measurements were set as described for Panel 21, except for the adjustment of retention time window to 7 min. For each analytical run 500 ng plasma peptides mixed with TQL spike-in pool (ranging from 24 fmol to 2 pmol per peptide) were injected. Data analysis was carried out as described for Panel 21.

The processed quantitative PRM data are provided in the Supplementary Information (Supplementary Data 5 and 6). The PRM raw data have been deposited to the ProteomeXchange Consortium via the PRIDE partner repository with the dataset identifier PXD079232.

### Calculation of absolute protein concentration

Absolute peptide concentration in each patient sample was determined from calibration curves, constructed with seven-point serial dilution of heavy labeled TQL peptides (range: 0.4–2000 fmol) in pooled human plasma (500 ng/µL) to account for matrix effects. Linear regression analysis using natural log transformed values of each calibration curve was performed, and upper (ULOQ), as well as lower limits of quantitation (LLOQs) were defined. Endogenous protein concentration was calculated based on plasma volume analyzed and is expressed in μg/mL (Supplementary Data 6c). Resulting values were largely in agreement with published concentrations (Human Protein Atlas, https://www.proteinatlas.org/humanproteome/blood). For AHSG we observed a ten-fold lower range compared to the Human protein atlas reference (average 12.24 μg/mL compared to 110 μg/mL, https://www.proteinatlas.org/ENSG00000145192-AHSG/blood). However, AHSG values reported here are in line with recently published MRM data^32^, 2.5–5 μg/mL). F9 concentration values within the cohorts were found to be lower than commonly reported, which is consistent with strong reduction of F9 plasma level in systemic inflammatory diseases (https://www.proteinatlas.org/ENSG00000101981-F9/blood). Absolute peptide concentrations were subjected to Variance Stabilization Normalization (VSN) to achieve approximately constant variance across the dynamic range of quantified values. Although VSN does not guarantee perfect normality for each peptide’s distribution, it ensures homoscedasticity required by downstream statistical methods^53^.

### Data analyses

#### Differential protein abundance calculation in discovery cohort

Proteomic profiling was performed on plasma samples from 163 individuals, measuring the abundance of 605 proteins. Healthy controls (*n* = 50) were excluded from disease activity analyses, yielding 113 patients stratified into remission (*n* = 54) and active disease (*n* = 59) groups. Within each antigen-specific subgroup (MPO-AAV and PR3-AAV), log₂ fold-changes between active and remission samples were computed. Statistical significance was assessed by Welch’s two-sample t-test, followed by Benjamini–Hochberg false discovery rate (FDR) correction (α = 0.05).

### Confounder assessment

#### Confounder-aware feature selection with metadeconfoundR

To identify proteins whose association with AAV disease activity was not statistically reducible to clinical covariates, we applied MetaDeconfoundR (v1.0.4), a rank-based confounder-aware feature selection framework. Protein abundance (605 features) and patient metadata were supplied as input. Metadata comprised disease activity status (active vs. remission, first column), age, sex, creatinine, eGFR, CRP, hemoglobin, hematocrit, leukocytes, neutrophils, platelets, ANCA titer, and binary indicators for organ involvement (kidney, lung, ENT, muscle/joints, skin/mouth/eye, CNS). The method proceeds in two steps. First, univariate associations between each protein and each metadata variables are tested using rank-based statistics (Wilcoxon test for binary, Spearman correlation for continuous variables), with FDR correction applied across all features–metadata pairs. Second, for each protein–metadata association passing the significance (*q* < 0.1) and effect size ( | Cliff’s δ | > 0.1) thresholds, nested linear models are fitted to rank-transformed protein abundances and compared by likelihood ratio test to determine whether the disease activity association is statistically reducible to any other covariate. Each protein-disease activity association is assigned one of five status labels: strictly deconfounded (OK_sd: association not reducible to any covariate), trivially deconfounded (OK_nc: no other significant covariates present), ambiguously deconfounded (AD), confounded (C), or not significant (NS). Only proteins classified as OK_sd or OK_nc were retained for downstream modeling, yielding 135 confounder-independent proteins. (Supplementary Data 3). In this application, the term ‘confounding status’ refers strictly to statistical reducibility within the observed metadata structure, that is, whether a protein–disease activity association can be explained by co-variation with another measured variable, and does not imply causal confounding in the epidemiological sense. Accordingly, proteins classified as OK_sd, OK_nc or AD were interpreted as having covariate-independent associations with disease activity, not as being causally independent of these variables.

#### Gene ontology enrichment

Functional enrichment analysis was performed using the g:Profiler version e113_eg59_p19_6be52918^54^, applying the Benjamini–Hochberg multiple testing correction method with a significance threshold of 10%. An analysis of differentially expressed proteins (DEPs) between active PR3-AAV and healthy controls (HCs), and between active MPO-AAV and HCs was conducted. These analyses were performed against a customized landscape of totally quantified *n* = 605 proteins for statistical testing. To facilitate concise biological interpretation, redundancy among the enriched Gene Ontology (GO) terms was reduced using the REVIGO (Reduce and Visualize Gene Ontology, version 1.8.2) web tool^55^. The ‘small’ 0.5 similarity cut-off was applied, and the UniProt human database (04/09/2025) was used as the reference for GO term sizes. We further refined the GO term list by applying a hierarchical depth filter using AmiGO 2 (version 2.5.17). Terms located at root level (levels 1–3) of the Gene Ontology tree were excluded, as were terms with a direct parent–child relationship, to prioritize terms with higher information content and biological specificity. For visualization of signature sets, median log2 intensity for each term was calculated per patient. Statistical analysis was performed using the GraphPad® Prism V.8.4.1 software (La Jolla, USA). All values are shown as scatter dot plots with median. Mann-Whitney U test was applied (*p < 0.05, **p < 0.01, ***p < 0.001).

#### Machine learning model development, feature selection, and classifier construction

Candidate proteins independently associated with disease activity after adjustment for clinical variables, including CRP, eGFR, and ANCA, were identified using MetaDeconfoundR (*n* = 135 proteins). The discovery cohort was split 80/20 into training (*n* = 88) and test (*n* = 22) sets prior to any modeling, stratified by outcome (set.seed(123)). LASSO-penalized logistic regression (glmnet) was applied to the global DEPs and subsequently to the PRM training data. Peptides with the largest absolute LASSO coefficient were selected. Boruta feature selection (set.seed(42), maxRuns = 100) was then applied within the PRM training data, identifying the final 7-protein panel. The predictor set was fixed at this point, prior to any access to the external validation cohort or the pooled validation data described below.

The 7-protein logistic regression model was first refit on the full discovery cohort and applied without modification to the independent external validation cohort (*n* = 108). Subsequently, for the primary validation, all patients were pooled and randomly assigned 60% to training (Train60, *n* = 127, EPV = 8.86) and 40% to test (Test40, *n* = 85), stratified by remission status and cohort (set.seed(7)). The 7-protein formula was refitted on Train60 and applied once to Test40. Model performance was assessed in both held-out sets, no model parameter was adjusted based on validation results.

Discrimination was quantified by AUC with 95% CIs by DeLong method and non-parametric bootstrap (2,000 resamples). Panel discrimination was compared against CRP alone, ANCA alone, and their combination using DeLong’s test for correlated ROC curves. Threshold-dependent metrics (sensitivity, specificity, PPV, NPV) were computed at a fixed 0.5 probability threshold with exact Clopper–Pearson 95% CIs, alongside confusion matrix counts. Calibration was assessed by calibration slope, calibration-in-the-large, Brier score (bootstrap 95% CI, 2000 resamples), and Hosmer–Lemeshow test. Clinical utility was evaluated by decision curve analysis (dcurves, net benefit differences vs. CRP with 95% CI by paired bootstrap, 1,000 resamples). A concentration-based version of the panel, converting PRM intensities to VSN-normalized absolute concentrations, was evaluated post-hoc on the same Test40 split.

Quasi-complete separation in both training sets was evaluated transparently: Firth penalized logistic regression confirmed separation did not inflate the AUC in either case (ΔAUC ≤ 004). A Ridge-penalized sensitivity analysis confirmed discrimination reflects genuine protein signal rather than inflated coefficients (external validation AUC 0.95 vs. GLM 0.94, DeLong *p* > 0.05, ~10-fold coefficient shrinkage) this model is reported as a supplementary sensitivity analysis only. Overfitting in the primary 60/40 validation was assessed by a full refit-permutation test (2,000 resamples, permutation *p* = 0.744), confirming the train/test AUC gap of 0.001 is not distinguishable from chance variation.

### Software and statistical environment

All analyses were conducted in R version 4.1.2, employing the tidyverse (v1.3.1), caret (v6.0-94), pROC (v1.18.5), glmnet (v4.1-7), enrichGO (v3.0.4), metadeconfoundR (v0.3.0), and rmda (v1.6) packages. Data visualization was performed with ggplot2 (v3.5.1).

### Statistics

For the statistical workflow see Method section. Details of presented statistical results are described in the respective figure legends.

### Ethics

The study was approved by the local ethic committee of Charité-Universitätsmedizin Berlin, Germany (EA4/025/18) and the General University Hospital Prague, Czech Republic (No. 1443/11 S-IV). Patients and healthy controls (HC) gave written, informed consent.

### Reporting summary

Further information on research design is available in the Nature Portfolio Reporting Summary linked to this article.

## Supplementary information

Supplementary Information

Description of Additional Supplementary Files

Supplementary Data 1

Supplementary Data 2

Supplementary Data 3

Supplementary Data 4.

Supplementary Data 5

Supplementary Data 6

Reporting Summary

Peer Review file

## Source data

Source Data

## Data Availability

The mass spectrometry proteomics raw data generated in this study have been deposited to the ProteomeXchange Consortium via the PRIDE partner repository with the dataset identifier ProteomeXchange Dataset PXD079232. Processed quantitative proteomics data are provided in the Supplementary Information (Supplementary Data 1, 5 and 6). Source data for all figures are provided in the Source Data file. Source code, frozen model objects, and full reproducibility documentation including all random seeds are provided at GitHub: https://github.com/Theda-sys/vasculitis-remission-proteomics (10.5281/zenodo.20450403). Source data are provided with this paper.

## Code availability

All relevant code used for the analysis of data in this work are freely available and provided at GitHub: https://github.com/Theda-sys/vasculitis-remission-proteomics. (10.5281/zenodo.20450403).
