# Supplementary material for: Towards a proteomic plasma biomarker panel for diagnosing vasculitis remission
Source: Nat Commun. 2026 Jul 21;17:6825. doi: 10.1038/s41467-026-75755-6 (PMC13389191; doi:10.1038/s41467-026-75755-6)
Supplement: Supplementary file 1 — Supplementary Information [file 41467_2026_75755_MOESM1_ESM.pdf]

Supplementary Information

Towards a proteomic plasma biomarker panel for diagnosing vasculitis remission

Uwe Jerke, Marieluise Kirchner, Theda UP Bartolomaeus, and al.

Content:

Supplementary Figure 1: Global proteome evaluation.

Supplementary Figure 2: Covariate-aware feature selection and 21-protein candidate panel.

Supplementary Figure 3: Calibration diagnostics and PRM–global proteome concordance for the 7-protein panel.

Supplementary Figure 4: Calibration of the discovery cohort-trained GLM and Ridge sensitivity model in the external validation cohort.

Supplementary Figure 5: Overfitting assessment, protein-level contributions, and subgroup performance in the pooled 60/40 validation.

Supplementary Figure 6: Calibration and overfitting assessment for the concentration-based 7-protein panel in the pooled 60/40 validation.

Supplementary Table 1: Discovery cohort (Berlin): demographic and clinical characteristics of HC and AAV patients.

Supplementary Table 2: Discovery cohort (Berlin), global DIA proteome, 80/20 split, LASSO regression: 21-protein candidate coefficients and direction.

Supplementary Table 3: Discovery cohort, 21-protein panel: gene/protein annotation and biological process mapping.

Supplementary Table 4: Discovery cohort (Berlin), PRM, 80/20 split, LASSO peptide-level: peptide-to-protein consolidation.

Supplementary Table 5: Discovery cohort (Berlin), PRM, Boruta + LRT/AIC/RF consensus: feature selection to final 7-protein panel.

Supplementary Table 6: Discovery cohort (Berlin), PRM, 80/20 split (Train80/Test20), 7-protein GLM: discrimination, calibration and separation diagnostics.

Supplementary Table 7: Discovery cohort (Berlin), PRM, Train80, 7-protein GLM vs Ridge: coefficient comparison (separation-driven inflation).

Supplementary Table 8: Discovery cohort (Berlin), PRM, 80/20 split, individual proteins and 7-protein panel: AUC, train vs test.

Supplementary Table 9: Validation cohort (Prague), AAV patients: demographic and clinical characteristics.

Supplementary Table 10: TQL data, validation cohort (external, n=108), 7-protein GLM and Ridge (discovery-trained): discrimination, calibration and separation diagnostics.

Supplementary Table 11: TQL data, discovery training set (full, n=104), 7-protein GLM vs Ridge: coefficient comparison (separation-driven inflation).

Supplementary Table 12: TQL data, validation cohort (n=108, 54 events), 7-protein GLM vs CRP/ANCA ± combined: discrimination and threshold metrics (0.5 cut-off).

Supplementary Table 13: TQL data, validation cohort, 7-protein GLM: discrimination by eGFR and treatment subgroup.

Supplementary Table 14: TQL data, validation cohort, 7-protein GLM vs comparators: DeLong AUC comparison (ΔAUC and p-values).

Supplementary Table 15: TQL data, validation cohort, 7-protein GLM: AUC comparison, eGFR ≤45 vs >45 mL/min/1.73m².

Supplementary Table 16: TQL data, validation cohort, 7-protein GLM: AUC comparison across treatment strata (Naïve/GC/GC+CYC+RTX).

Supplementary Table 17: TQL data, validation cohort (post hoc), Ridge 7-protein panel vs CRP/ANCA: discrimination and threshold metrics (0.5 cut-off).

Supplementary Table 18: TQL data, pooled cohort (Train60→Test40, n=85), 7-protein panel: discrimination, calibration and separation diagnostics.

Supplementary Table 19: TQL data, pooled cohort, 7-protein panel: actual vs predicted median ratios by disease state.

Supplementary Table 20: TQL data, pooled Test40 (n=85), 7-protein panel: discrimination by ANCA specificity (MPO vs PR3).

Supplementary Table 21: TQL data, pooled Test40 (n=85), 7-protein panel vs CRP/ANCA ± combined: discrimination and threshold metrics (0.5 cut-off).

Supplementary Table 22: TQL data, pooled Test40, 7-protein panel vs comparators: DeLong AUC comparison (ΔAUC and p-values).

Supplementary Table 23: TQL data, pooled Test40, 7-protein panel vs CRP: AUC stratified by ANCA/CRP status plus likelihood ratios.

Supplementary Table 24: TQL data, pooled Test40 (n=85), 7-protein panel vs CRP alone: decision curve analysis — ΔNB by threshold.

Supplementary Table 25: TQL data, pooled Test40, 7-protein panel vs ANCA alone: decision curve analysis — ΔNB by threshold.

Supplementary Table 26: TQL data, pooled Test40, 7-protein panel vs ANCA+CRP: decision curve analysis — ΔNB by threshold.

Supplementary Table 27: TQL data, Test40 by cohort subset, 7-protein panel vs CRP/ANCA/ANCA+CRP: cohort-specific net benefit difference.

Supplementary Table 28: TQL data, pooled Test40, 7-protein panel, CRP, ANCA, ANCA+CRP: net benefit by threshold (decision-curve values).

Supplementary Table 29: 24-month follow-up, pooled cohort (n=96), 7-protein score (pre-trained, no refit): flare/relapse prediction — AUC, Cox, KM.

Supplementary Table 30: Concentration data, pooled cohort (Test40), 7-protein panel, absolute concentration (VSN): discrimination, calibration and separation diagnostics.

Supplementary Table 31: Concentration data, pooled Test40, 7-protein panel, absolute concentration (VSN): threshold metrics (0.5 cut-off).

Supplementary Table 32: Reporting checklist, TRIPOD adherence: item-by-item development and external validation assessment.

Supplementary Figure 1

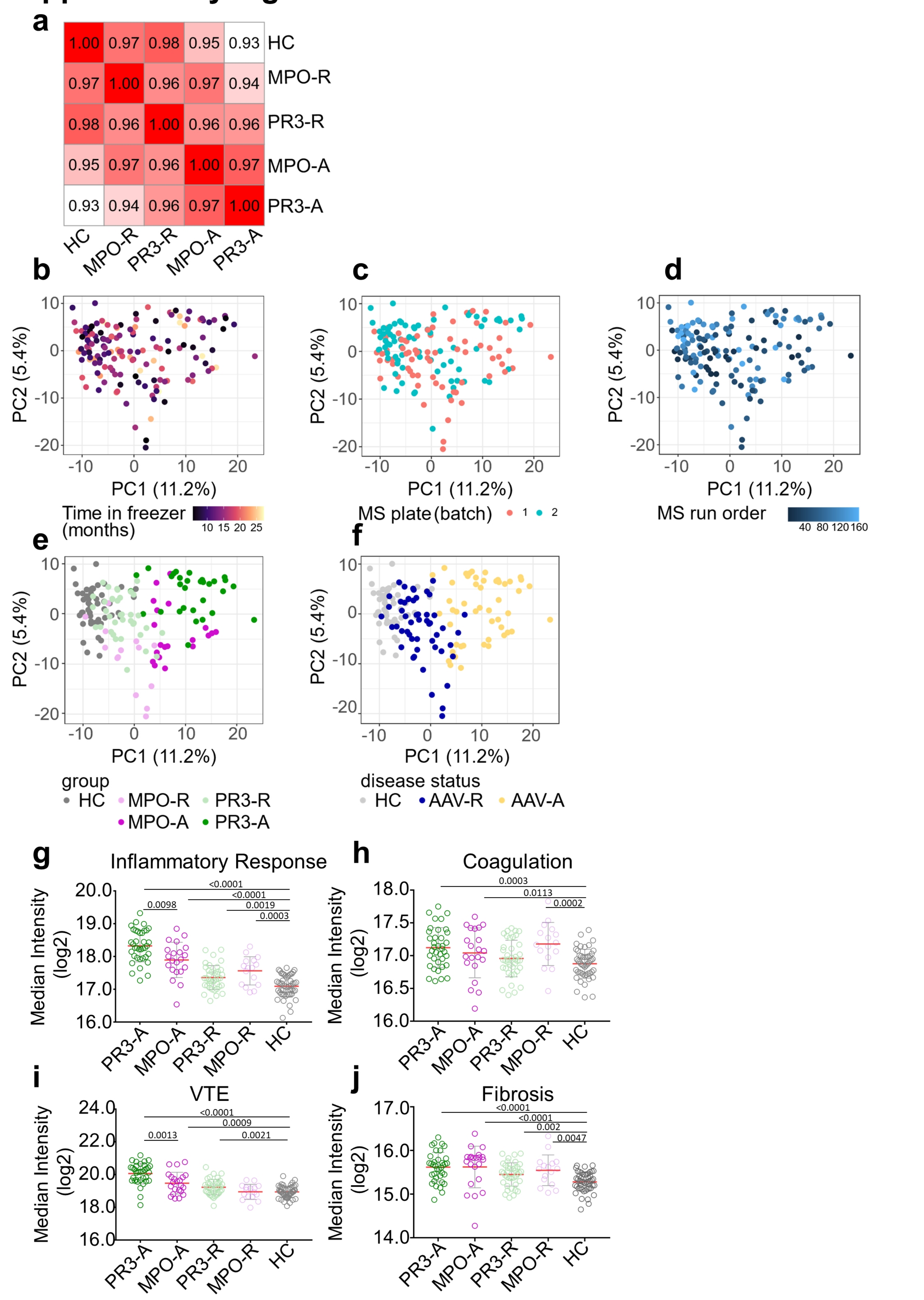

Supplementary Figure 1. Global proteome evaluation. (a) Spearman correlation heatmap of median protein intensities across patient groups. Pairwise Spearman correlation coefficients are shown based on median protein intensities for each patient group. Higher values indicate greater similarity in protein expression profiles. Color scale ranges from white (low correlation) to red (high correlation), with coefficients shown in each cell. **(b-f)** Principal component analysis (PCA) of the 605-protein matrix (centered and scaled) colored by technical and biological covariates. **(b)** Time in freezer (months from blood draw to MS measurement), **(c)** MS plate/batch identity (batch 1 (n=71) vs. batch 2 (n=81)), **(d)** MS run order, **(e)** clinical group (HC (n=50), MPO-R (n=14), PR3-R (n=37), MPO-A (n=20), PR3-A (n=31)), **(f)** disease status (HC (n=50), AAV remission (n=51) , AAV active (n=51)). PC1 and PC2 explain 11.2% and 5.4% of total variance, respectively. Linear model and PERMANOVA analyses confirmed that disease status was the dominant source of variance ($R^2 = 0.342$, $p = 0.001$), whereas freezer storage time ($R^2 = 0.008$, $p = 0.259$), MS plate ($R^2 = 0.012$, $p = 0.123$), and MS run order ($R^2 = 0.010$, $p = 0.157$) each explained less than 1.2% of variance and were non-significant, indicating that recorded technical variables do not materially influence the observed proteome structure. **(g-j)** Scatter dot plots showing median log_2 protein intensity for pathway-level protein signatures across clinical groups (n = 50 HC, n = 36 PR3-A, n = 22 MPO-A, n = 39 PR3-R, n = 16 MPO-R, each dot represents one patient): **(g)** inflammatory response, **(h)** coagulation, **(i)** venous thromboembolism (VTE), and **(j)** fibrosis. Mean and SD are presented, statistical comparisons by two-tailed Mann–Whitney U test was performed. The panel provides precise p-values for the comparisons illustrated. (MS = mass spectrometry measurement).

Supplementary Figure 2

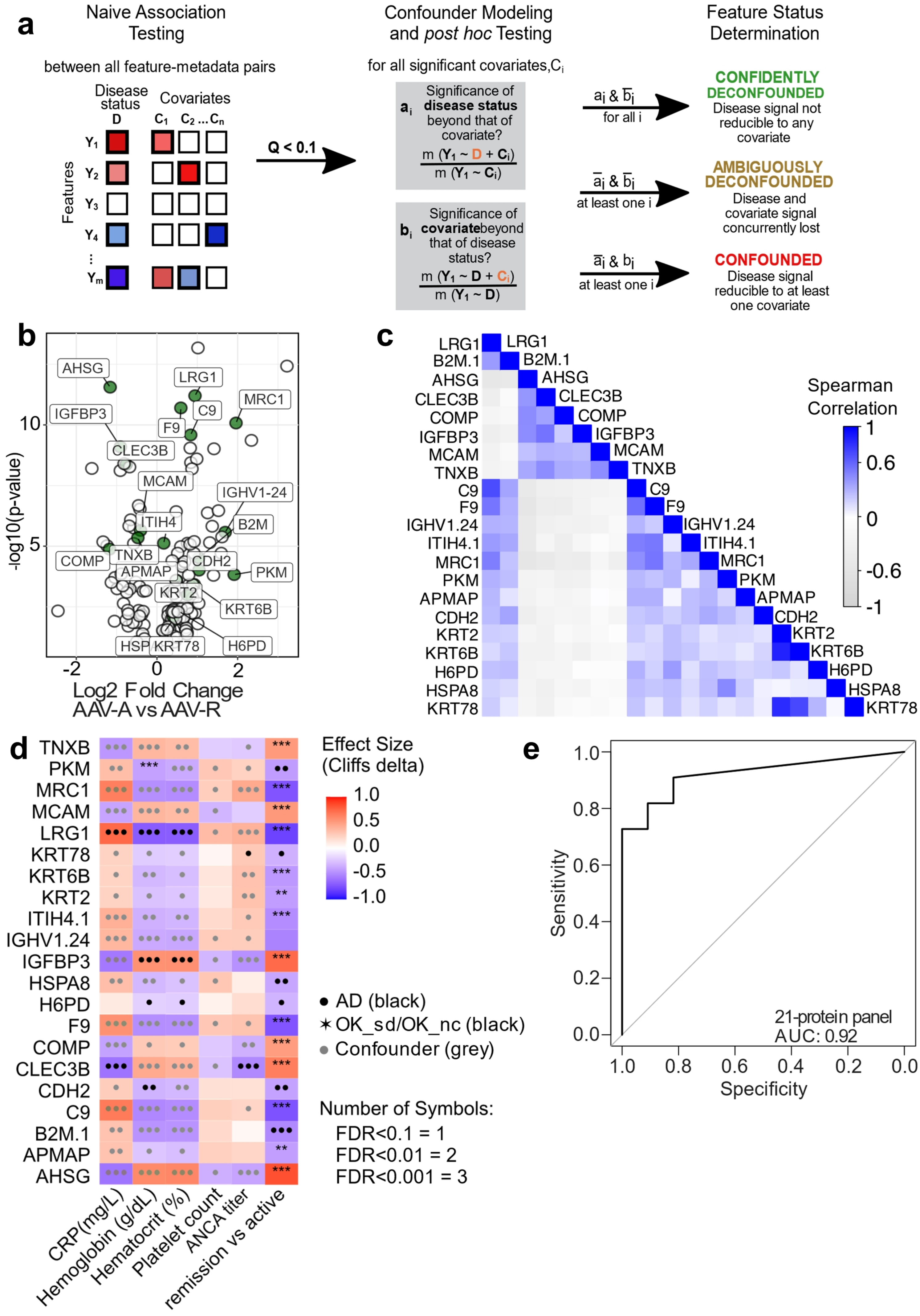

Supplementary Figure 2. Covariate-aware feature selection and 21-protein candidate panel. (a) Schematic overview of the confounder control framework for feature selection. The workflow consists of three steps: (1) Naïve association testing is performed between all features ($Y_1 \ldots Y_m$) and metadata variables, including disease status (D) and potential covariates ($C_1 \ldots C_n$). associations passing FDR correction ($q < 0.1$) proceed to post hoc testing. (2) Confounder modeling: for each significant feature-covariate pair, two nested likelihood ratio tests are performed: (a_i) tests whether disease status explains feature variation beyond the covariate, (b_i) tests whether the covariate explains feature variation beyond disease status. (3) Feature status is assigned based on the outcome of these tests: CONFIDENTLY DECONFOUNDED (a_i & $\bar{b}_i$ for all i): Disease signal is not reducible to any covariate. AMBIGUOUSLY DECONFOUNDED ($\bar{a}_i$ & $\bar{b}_i$ for at least one i): both disease and covariate signals are lost when controlling for each other, CONFOUNDED ($\bar{a}_i$ & b_i for at least one i): disease signal is reducible to at least one covariate. MetaDeconfoundR was applied to the full 605-protein dataset with the following covariates: age, sex, creatinine, eGFR, CRP, hemoglobin, hematocrit, leukocytes, neutrophils, platelets, ANCA titer, and binary organ involvement indicators (kidney, lung, ENT, muscle/joints, skin/mouth/eye, CNS). Only proteins classified as OK_sd or OK_nc were retained, yielding 135 covariate-independent proteins. **(b)** Volcano plot of 135 covariate-independent proteins comparing active (n = 59) and remission (n = 54) AAV patients. The x-axis shows the $\log_2$ fold change, and the y-axis shows the $-\log_{10}$ p-value (Welch's T-test, BH-FDR corrected). The 21 LASSO-selected candidate proteins are shown as filled dark green circles and labeled with gene names, significantly and non-significantly differentially expressed proteins are shown as black and grey open circles, respectively. **(c)** Spearman correlation matrix to of the 21 LASSO-selected candidate proteins, illustrating pairwise multicollinearity. **(d)** MetaDeconfoundR heatmap for the 21 LASSO-selected candidate proteins showing effect sizes (Cliff's delta) for associations with CRP, eGFR, hemoglobin, hematocrit, platelet count, disease activity status (remission vs. active), and ANCA titer. Tile color encodes effect size (red: positive, blue: negative). Symbol overlays indicate confounding status after FDR correction: asterisks denote OK_sd or OK_nc associations, filled circles denote AD associations, and grey circles denote associations confounded by another covariate. All 21 proteins show covariate-independent disease associations. For the majority of proteins, associations with CRP, hemoglobin, hematocrit, and platelets are classified as confounded by disease activity, indicating these correlations are driven by shared disease burden rather than independent covariate effects. **(e)** ROC curve of a GLM trained on the 21-protein candidate panel in the 80% discovery training set (n = 88) and applied once to the held-out 20% test set (n = 22).

Supplementary Figure 3

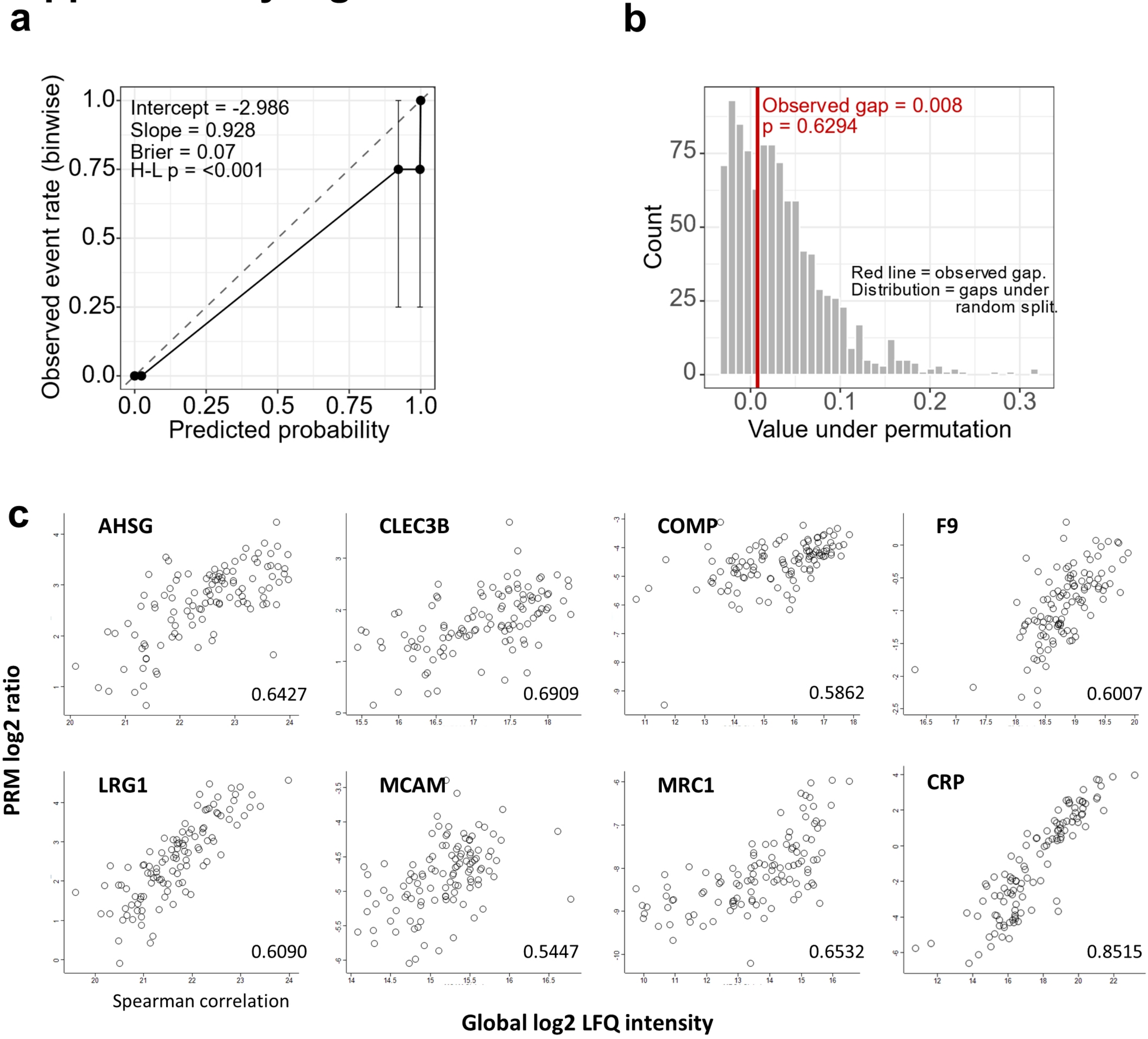

Supplementary Figure 3. Calibration diagnostics and PRM–global proteome concordance for the 7-protein panel. (a) Calibration plot of the GLM trained on the 80% discovery training set (n = 88) and evaluated in the held-out 20% test set (n = 22). Observed event rates (binwise) are plotted against predicted probabilities, the dashed line indicates perfect calibration. Data are presented as mean and SD. Performance metrics are shown in the plot. **(b)** Refit-permutation overfitting test for the 80/20 discovery split. The histogram shows the distribution of train–test AUC gaps under 2,000 random split permutations, the red vertical line indicates the observed gap.**(c)** Concordance between global proteome and PRM data for the 7-panel proteins and CRP (n = 102). Scatter plots show the log2 transformed LFQ intensity from global proteome measurements (x-axis) and the log2 light/heavy peptide ratios from PRM-MS (y-axis). Spearman correlation was calculated and coefficients are shown on the lower right quadrant of each plot.

Supplementary Figure 4

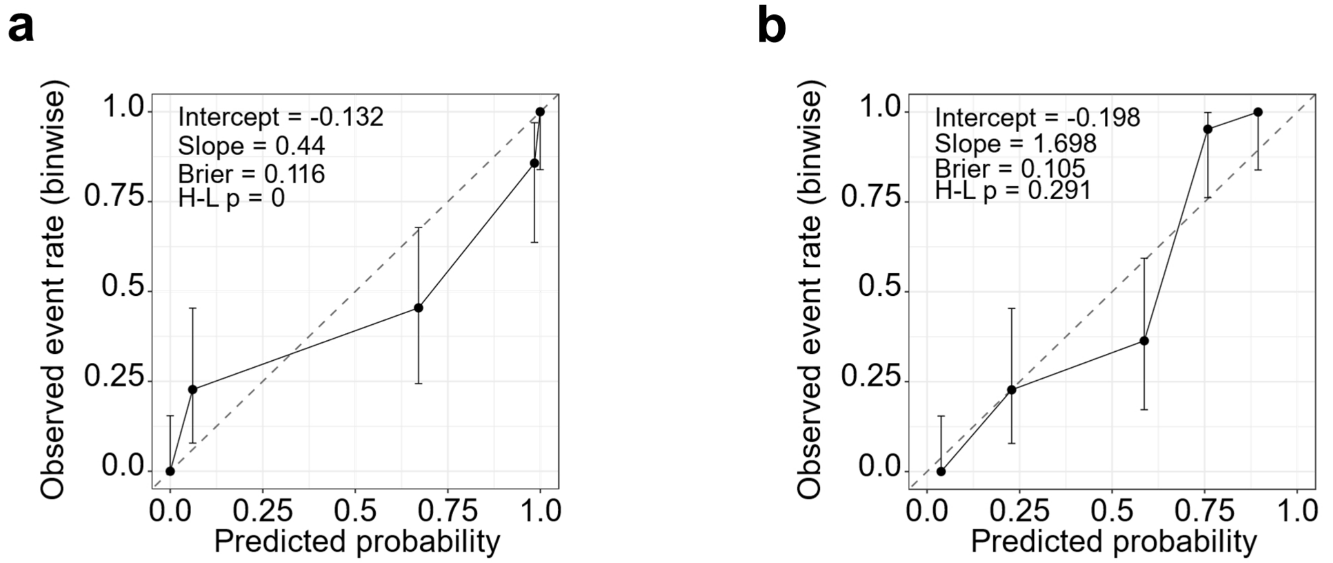

Supplementary Figure 4. Calibration of the discovery cohort-trained GLM and Ridge sensitivity model in the external validation cohort. (a) Calibration plot of the GLM trained on the full discovery cohort and applied to the external validation cohort (n = 108). Observed event rates (binwise) are plotted against predicted probabilities. The dashed line indicates perfect calibration. Performance metrics are shown in the plot. **(b)** Calibration plot of the Ridge-penalized sensitivity model trained on the discovery cohort and applied to the external validation cohort. Performance metrics are shown in the plot. Ridge model was selected following inspection of these calibration metrics. The external validation cohort does not constitute a fully independent validation for this model and the results are therefore reported as a sensitivity analysis only. Data are presented as mean values and SD.

Supplementary Figure 5

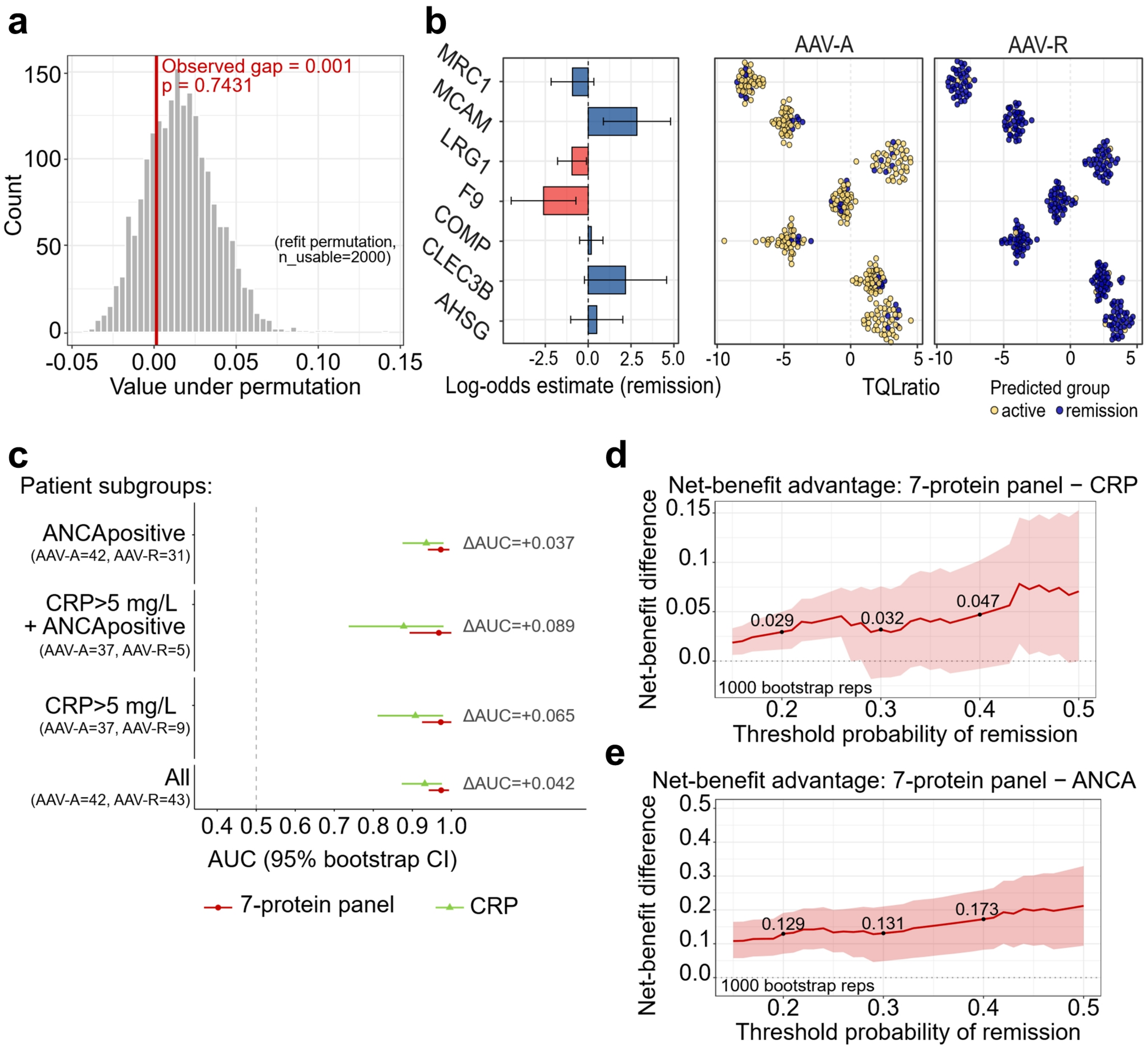

Supplementary Figure 5. Overfitting assessment, protein-level contributions, and subgroup performance in the pooled 60/40 validation. (a) Refit-permutation overfitting test for the pooled 60/40 split. The histogram shows the distribution of train–test AUC gaps under 2,000 random refit permutations, the red vertical line indicates the observed gap (0.001, p = 0.743), confirming no evidence of overfitting. **(b)** Left: box plots showing the distribution of TQL $\log_2$ light/heavy ratios for each of the seven panel proteins in the Train60 dataset (n = 127), stratified by disease status (active: yellow, remission: blue). Exact p-values for significant differences between groups are provided. Center and right: scatter plots of individual TQL ratios (x-axis) against GLM-predicted remission probability (y-axis) for active (yellow, center) and remission (blue, right) patients in Test40 (n = 85), illustrating per-protein contribution to model predictions. **(c)** Forest plot comparing AUC of the 7-protein panel (red) versus CRP (green) across prespecified active (AAV-A) and remission (AAV-R) subgroups in Test40 (n = 85): all patients, patients with CRP > 5 mg/L, with CRP > 5 mg/L and ANCA positive, and all ANCA positive patients. Points represent AUC estimates with 95% bootstrap confidence intervals. ΔAUC values indicate the advantage of the 7-protein panel over CRP alone within each subgroup. **(d, e)** Net benefit difference between the 7-protein panel and CRP alone **(d)** and between the 7-protein panel and ANCA titer **(e)** across threshold probabilities for remission (0.15–0.50). The solid line shows the mean paired net benefit difference and the shaded band the 95% bootstrap confidence interval (1,000 resamples). Positive values indicate that the 7-protein panel provides greater net benefit than either comparator across the full clinical decision range.

Supplementary Figure 6

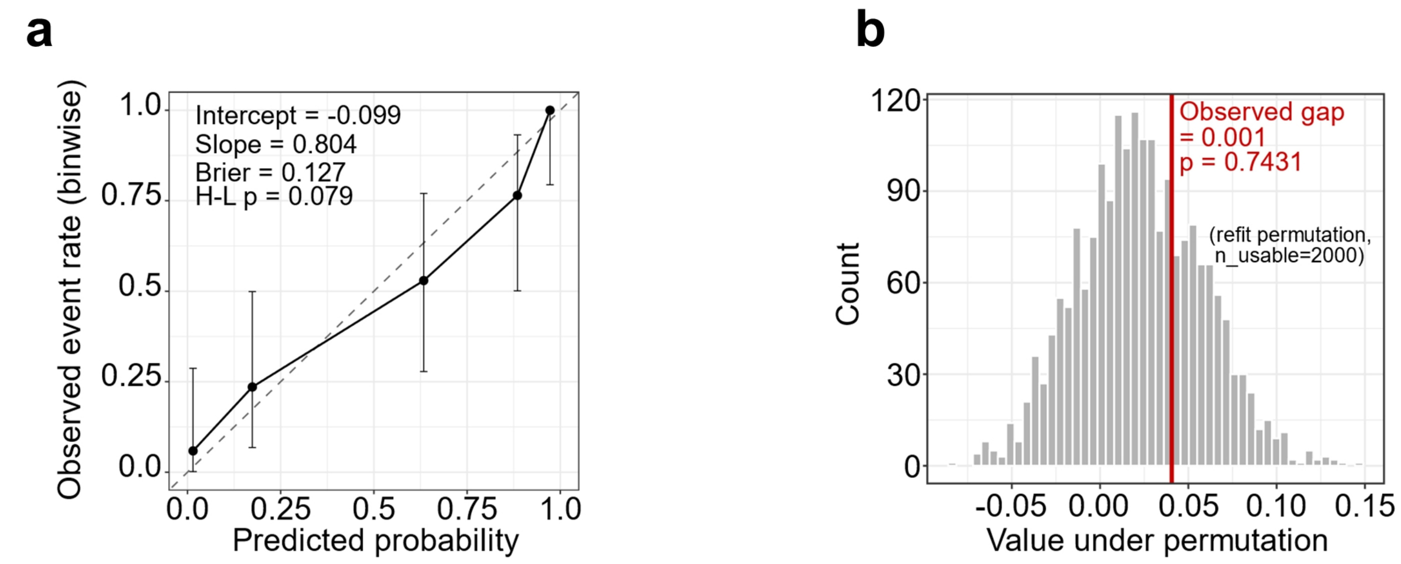

Supplementary Figure 6. Calibration and overfitting assessment for the concentration-based 7-protein panel in the pooled 60/40 validation. (a) Calibration plot of the concentration-based GLM (VSN-normalized absolute peptide concentrations) evaluated in the held-out Test40 partition (n = 85). Observed event rates (binwise) are plotted against predicted probabilities. Data are presented as mean and SD. The dashed line indicates perfect calibration. Performance metrics are shown in the plot. **(b)** Refit-permutation overfitting test for the concentration-based model. The histogram shows the distribution of train–test AUC gaps under 2,000 random refit permutations, the red vertical line indicates the observed gap (0.001, p = 0.743).

Supplementary Table 1. Discovery cohort (Berlin): demographic and clinical characteristics of HC and AAV patients.

	Diagnosis				
	**HC**	**active PR3-AAV**	**rem. PR3-AAV**	**active MPO-AAV**	**rem. MPO-AAV**
n	50	37	38	23	16
Age -year	53	60.6±15.6	63.5±12.4	66.3±12.5	56.4±16.2
Female -no. (%)	27 (54)	16 (43)	18 (49)	9 (39)	9 (56)
Female -no. (%)	23 (46)	21 (57)	19 (51)	14 (61)	7 (44)
**Disease entity**					
GPA	-	37	38	0	0
MPA	-	0	0	20	16
EGPA	-	0	0	3	0
**Clinical parameters**					
BVAS (0-63)	-	16.8±7.1	-	17.7±5.2	-
ANCA titer (ELISA, U/mL)	-	141±64<br>●<0.001<br>*<0.001	63±67<br>*<0.001	76±37<br>●<0.001<br>*<0.001	24±26<br>*<0.001
CRP (mg/L)	-	73.0±64<br>●0.032<br>*<0.001	5.3±9.3<br>*<0.001	37.9±57.4<br>●0.032<br>*0.024	8.0±14.2<br>*0.024
Hematuria (dipstick. 0-3)	-	2.5±1.0<br>*<0.001	0.4±0.8<br>*<0.001	2.3±0.9	1.3±1.3
Creatinine (mg/dL)	-	3.0±2.3<br>*<0.001	1.4±0.8<br>*<0.001	2.8±2.2	1.8±1.1
Hemoglobin (g/dL)	-	10.0±2.0<br>*<0.001	13.6±1.3<br>*<0.001	10.5±1.6<br>*<0.001	12.8±1.9<br>*<0.001
Hematocrit (%)	-	30.2±6.2<br>*<0.001	40.9±3.5<br>*<0.001	31.2±4.4<br>*<0.001	38.1±5.2<br>*<0.001
eGFR (ml/min/1,73 m²)	-	41.7±37.6<br>*0.041	57.2±26.0<br>*0.041	30.5±18.4<br>*0.015	51.7±28.2<br>*0.015
Leukocytes (/nL)	-	13.6±6.0<br>●0.023<br>*<0.001	8.2±2.1<br>*<0.001	10.5±4.3<br>●0.023	8.4±2.7
Neutrophils (/nL)	-	11.4±5.7<br>●0.002<br>*<0.001	5.7±1.9<br>*<0.001	7.5±3.5<br>●0.002	5.6±2.3
Platelets (/nL)	-	435±215<br>*<0.001	263±66<br>*<0.001	343±105	277±82
**Organ involvement**					
Kidney -no. (%)	-	29 (78)	-	19 (83)	-
Lung -no. (%)	-	27 (73)	-	11 (48)	-
Ear/Nose/Throat -no. (%)	-	20 (54)	-	5 (22)	-
Muscle/Joints -no. (%)	-	13 (35)	-	4 (17)	-
Skin/ Mouth/Eyes -no. (%)	-	12 (32)	-	1 (4)	-
Central nervous system -no. (%)	-	5 (14)	-	5 (22)	-

**Induction Treatment**					
Naïve – no. (%)	-	7 (18)	-	10 (43)	-
Glucocorticoid (GC) -no. (%)	-	15 (41)	-	7 (30)	-
GC + Cyclophoshamide/ rituximab -no. (%)	-	15 (41)	-	3 (13)	-
Unknown -no. (%)	-	-	-	3 (13)	-
**Maintenance Treatment**					
Months in remission at sampling	-	-	59.7±71.7	-	49.6±64.1
Naïve – no. (%)	-	-	6 (16)	-	3 (19)
Glucocorticoid (GC) -no. (%)		-	17 (45)	-	11 (69)
Mean Glucocorticoid dose (mg/d)		-	5.5±3.6	-	7.4±8.0
RTX – no. (%)		-	25 (66)	-	8 (50)
MTX – no. (%)		-	1 (3)	-	0 ()
AZA		-	0 ()	-	0 ()
Off-treatment – no. (%)#		-	18 (47)	-	6 (38)
On-treatment – no. (%)		-	19 (50)	-	7 (44)
Unknown – no. (%)		-	1 (3)	-	3 (19)
**Follow-up (24 month) – no.**		-	37	-	14
Flare – no. (%)		-	10 (27)	-	0 ()
minor flare – no. (%)		-	6 (16)	-	0 ()
major flare – no. (%)		-	4 (11)	-	0 ()
Sustained rem. – no. (%)§		-	24 (65)	-	13 (93)
Unknown – no. (%)		-	3 (8)	-	1 (7)

Data are presented as no or mean±SD. (#) Off treatment is defined as patients with less than 7.5 mg/d prednisolone and no other immunosuppressant currently or within the last 6 months. (§) Sustained remission means flare-free within the 24-month follow-up. (●) Indicates a significant difference between active PR3-AAV and active MPO-AAV as measured by two-tailed Welch's t-test or Chi-squared test ($P < 0.05$); (*) indicates a significant difference between active AAV and their corresponding remission group as measured by two-tailed Welch's t-test or Chi-squared test ($P < 0.05$). Precise p values of significant comparisons are provided behind corresponding symbol.

Supplementary Table 2. Discovery cohort (Berlin), global DIA proteome, 80/20 split, LASSO regression: 21-protein candidate coefficients and direction.

LASSO applied to 80% training split of the discovery DIA proteomics data (n = 88 training, 22 test). Lambda selected by 10-fold CV (lambda.min). Proteins listed in descending order of |coefficient|. Positive coefficients = higher abundance associated with active disease; negative = higher abundance in remission.

Gene	LASSO coefficient	Association direction
LRG1	−1.472	Remission ↑
F9	−1.499	Remission ↑
KRT78	−0.512	Remission ↑
C9	−0.561	Remission ↑
ITIH4.1	−0.414	Remission ↑
H6PD	−0.242	Remission ↑
HSPA8	−0.217	Remission ↑
KRT6B	−0.069	Remission ↑
MRC1	−0.041	Remission ↑
PKM	−0.039	Remission ↑
CDH2	−0.047	Remission ↑
KRT2	−0.035	Remission ↑
APMAP	−0.142	Remission ↑
IGHV1.24	−0.107	Remission ↑
B2M.1	−0.052	Remission ↑
AHSG	+0.255	Active ↑
CLEC3B	+0.217	Active ↑
COMP	+0.072	Active ↑
IGFBP3	+0.247	Active ↑
MCAM	+0.167	Active ↑
TNXB	+0.440	Active ↑

Supplementary Table 3. Discovery cohort, 21-protein panel: gene/protein annotation and biological process mapping.

Protein group ID	Gene name	Protein names	Compartment	Biological Processes
**<u>Immune Response and Acute Phase Proteins</u>**				
P02765	**AHSG**	**Alpha-2-HS-glycoprotein; Fetuin-A**	secretory	Inflammation, Immune response, Acute-phase response
P61769; H0YLF3	**B2M**	**Beta-2-microglobulin**	secretory	Immune response, Antigen presentation, MHC class I complex
P02748	**C9**	**Complement component C9**	secretory	Immune response, Complement activation, Inflammatory response
E9PHK0; P05452;	**CLEC3B**	**Tetranectin**	secretory, membrane	Innate immunity, Phagocytosis, Endocytosis
P00740	**F9**	**Coagulation factor IX**	secretory	Blood coagulation, Hemostasis
A0A0C4DH33	**IGHV1-24**	**Immunoglobulin heavy variable 1-24**	secretory	Antibody production, Immune response
B7ZKJ8	**ITIH4**	**ITIH4 protein; Inter-alpha-trypsin inhibitor heavy chain 4**	secretory	Inflammation, ECM regulation, Cell growth regulation
P02750	**LRG1**	**Leucine-rich alpha-2-glycoprotein 1**	secretory	Inflammation, Acute-phase response, Immune response
P22897	**MRC1**	**Macrophage mannose receptor 1**	membrane	Innate immunity, Phagocytosis, Antigen processing
**<u>Extracellular Matrix and Cell Adhesion</u>**				
Q9HDC9	**APMAP**	**Adipocyte plasma membrane-associated protein**	membrane	Cell adhesion, Cell differentiation, ECM remodeling
P19022	**CDH2**	**Cadherin-2**	membrane	Cell adhesion, Neuronal development, Tissue formation
G3XAP6; P49747	**COMP**	**Cartilage oligomeric matrix protein**	extracellular	ECM organization, Cartilage development
P43121	**MCAM**	**Cell surface glycoprotein MUC18**	membrane	Cell adhesion, Tumor metastasis, Cell migration

A0A140TA41; A0A140TA52; A0A3B3ISX9; P22105; P22105-1; P22105-4	**TNXB**	**Tenascin-X**	extracellular	ECM organization, Tissue remodeling, Cell adhesion
**Metabolism and Energy Regulation**				
O95479; O95479-2	**H6PD**	**Hexose-6-phosphate dehydrogenase**	cytoplasm	Carbohydrate metabolism, Pentose phosphate pathway
P14618	**PKM**	**Pyruvate kinase PKM**	cytoplasm	Glycolysis, Energy production, Metabolism
**Growth Regulation and Cellular Proliferation**				
A6XND0; A6XND1; P17936; P17936-2	**IGFBP3**	**Insulin-like growth factor binding protein 3**	secretory	Growth regulation, Cell proliferation, Apoptosis regulation
**Cellular Stress Response and Protein Folding**				
P11142	**HSPA8**	**Heat shock cognate 71 kDa protein**	Cytoplasm, nucleus	Protein folding, Stress response, Protein quality control
P35908	**KRT2**	**Keratin, type II cytoskeletal 2**	cytoplasm	Cytoskeleton organization, Cell structure, Epidermis formation
P04259	**KRT6B**	**Keratin, type II cytoskeletal 6B**	cytoplasm	Cytoskeleton organization, Epithelial cell structure
Q8N1N4	**KRT78**	**Keratin, type II cytoskeletal 78**	cytoplasm	Cytoskeleton organization, Keratin intermediate filament formation

Supplementary Table 4. Discovery cohort (Berlin), PRM, 80/20 split, LASSO peptide-level: peptide-to-protein consolidation.

LASSO applied to 80% training split of PRM-MS data (n = 88 training, 22 test). Lambda selected by 10-fold CV (lambda.min). Where multiple peptides from the same protein were retained, the peptide with the largest |coefficient| was selected (highlighted rows).

Peptide ID	LASSO coefficient	Protein (gene)	Selection status	Carried forward
MCAM_048	−3.467	MCAM	Selected (largest |coef|)	Yes
MCAM_046	−0.052	MCAM	Excluded (smaller |coef|)	No
AHSG_004	−2.116	AHSG	Selected (largest |coef|)	Yes
AHSG_002	−0.115	AHSG	Excluded (smaller |coef|)	No
AHSG_001	−0.047	AHSG	Excluded (smaller |coef|)	No
H6PD_031	+1.377	H6PD	Sole peptide	Yes
LRG1_042	+0.692	LRG1	Sole peptide	Yes
CDH2_014	+0.656	CDH2	Sole peptide	Yes
MRC1_073	+0.595	MRC1	Sole peptide	Yes
TNXB_101	−0.687	TNXB	Sole peptide	Yes
KRT78_039	−0.566	KRT78	Sole peptide	Yes
CLEC3B_020	+0.439	CLEC3B	Sole peptide	Yes
C9_076	+0.325	C9	Selected (largest |coef|)	Yes
C9_078	−0.061	C9	Excluded (smaller |coef|)	No
COMP_023	−0.300	COMP	Sole peptide	Yes
ITIH4_103	+0.249	ITIH4	Sole peptide	Yes
IGHV1-24_036	+0.133	IGHV1.24	Selected (largest |coef|)	Yes
IGHV1-24_036 Mox	+0.066	IGHV1.24	Excluded (smaller |coef|)	No
F9_026	+0.158	F9	Sole peptide	Yes
B2M_010	+0.058	B2M	Sole peptide	Yes
PKM_089	−0.043	PKM	Sole peptide	Yes
KRT6B_037	−0.029	KRT6B	Sole peptide	Yes
KRT2_085	-0.103	KRT2	Sole peptide	Yes

Supplementary Table 5. Discovery cohort (Berlin), PRM, Boruta + LRT/AIC/RF consensus: feature selection to final 7-protein panel.

Boruta (set.seed(42), maxRuns=100) was the primary criterion. Three secondary methods (LRT, stepwise AIC, Random Forest top-10) were used for triangulation. The 7 proteins Confirmed by Boruta. Proteins in the final panel are: AHSG, CLEC3B, COMP, F9, LRG1, MCAM, MRC1.

	Feature	Importance	Decision
**MCAM_047**	MCAM_047	9,235	Confirmed
**AHSG_004**	AHSG_001	14,325	Confirmed
**H6PD_031**	H6PD_030	0,488	Rejected
**LRG1_042**	LRG1_042	16,257	Confirmed
**TNXB_101**	TNXB_100	-0,039	Rejected
**CDH2_014**	CDH2_013.N	0,579	Rejected
**MRC1_073**	MRC1_073	6,201	Confirmed
**KRT78_039**	KRT78_039	2,362	Tentative
**CLEC3B_020**	CLEC3B_020	12,011	Confirmed
**C9_078**	C9_078	3,178	Confirmed
**COMP_023**	COMP_023	11,866	Confirmed
**ITIH4_103**	ITIH4_103	1,727	Rejected
**F9_026**	F9_027	3,008	Tentative
**B2M_010**	B2M_011	3,010	Tentative
**IGHV1.24_036**	IGHV1.24_036	-0,227	Rejected
**PKM_089**	PKM_089	0,385	Rejected
**KRT2_085**	KRT2_085	-1,358	Rejected
**KRT6B_037**	KRT6B_037	0,280	Rejected

Supplementary Table 6. Discovery cohort (Berlin), PRM, 80/20 split (Train80/Test20), 7-protein GLM: discrimination, calibration and separation diagnostics.

Model performance and separation diagnostics for the 7-protein GLM evaluated on the 80/20 internal discovery split (n = 110; Train80 n = 88, Test20 n = 22). Calibration metrics are computed on the 20% held-out test set. Overfitting and separation diagnostics refer to the 80% training set. Bootstrap CIs: percentile method, 2,000 resamples, seed = 7.

Metric	Value
Discrimination	
AUC (DeLong 95% CI)	0.975 (0.920 – 1.000)
Brier score (bootstrap 95% CI)	0.070 (0.000 – 0.184)
LOO-AUC range	0.972 – 1.000
Label-permutation p (test set, n = 2,000, one-tailed)	< 0.001
Label-permutation p (full cohort, n = 2,000, one-tailed)	< 0.001
Calibration (20% test set)	
Calibration intercept *	−2.986
Calibration slope *	0.928
Hosmer–Lemeshow p (one-tailed)	< 0.001
Mean predicted probability	0.536
Observed event rate (10 / 22)	0.455
Calibration-in-the-large (overestimation)	+0.082
Overfitting assessment (training set Test20)	
Apparent train AUC	0.983
Bootstrap optimism estimate (B = 2,000)	0.034
Optimism-corrected train AUC	0.949
Separation diagnostics (training set Test20)	
Extreme fitted probabilities (< 0.001 or > 0.999)	33% (29 / 88)
Max GLM / Ridge coefficient ratio (CLEC3B_020)	12.92×
Firth penalised AUC (ΔAUC vs GLM)	0.975 (Δ = 0.000)
Events per variable (EPV)	5.857
Calibration slope on test set	0.928

AUC = area under the ROC curve (DeLong method). EPV = events per variable (guideline ≥ 10). LOO = leave-one-out. ΔAUC = GLM AUC minus Firth AUC. Extreme fitted probabilities: proportion of training observations with predicted probability < 0.001 or > 0.999. Bootstrap optimism: mean difference between apparent and test AUC across 2,000 bootstrap resamples. Firth model uses penalised logistic regression (logistf package) to correct for quasi-complete separation.

** Bootstrap CI not estimable for calibration intercept and slope due to quasi-complete separation in the training set combined with n = 22 in the test set; point estimates reported only.*

† Brier score lower CI = 0.000 is a boundary artefact of bootstrap resampling on a small test set and should not be interpreted as a true minimum.

Supplementary Table 7. Discovery cohort (Berlin), PRM, Train80, 7-protein GLM vs Ridge: coefficient comparison (separation-driven inflation).

GLM versus Ridge coefficient comparison for the 7-protein panel fitted on the 80/20 discovery training set. The |GLM| / |Ridge| ratio quantifies separation-driven coefficient inflation; values substantially above 1 indicate inflated GLM coefficients consistent with quasi-complete separation. Five of seven proteins show >9-fold inflation. MRC1_073 and COMP_023 are unaffected, suggesting their contributions are robust to separation. The Firth penalised model a), which directly corrects for separation-induced inflation, yields an identical test AUC (ΔAUC = 0.000), confirming that discrimination is not driven by inflated coefficients.

Protein	GLM coefficient	Ridge coefficient	|GLM| / |Ridge| ratio
CLEC3B_020	−2.409	0.187	12.92×
MCAM_047	7.005	0.664	10.54×
AHSG_001	4.606	0.442	10.42×
F9_027	−3.179	−0.306	10.38×
LRG1_042	−3.954	−0.421	9.40×
MRC1_073	−0.323	−0.248	1.30×
COMP_023	0.459	0.443	1.04×

Supplementary Table 8. Discovery cohort (Berlin), PRM, 80/20 split, individual proteins and 7-protein panel: AUC, train vs test.

Individual protein and 7-panel AUC estimates (95% CI) in the PRM 80/20 training and test sets. Train and test AUCs are shown separately to allow assessment of consistency across the split.

Protein	AUC train	95% CI train	AUC test	95% CI test
MCAM_047	0.796	0.703 - 0.888	0.967	0.907 - 1.00
AHSG_001	0.857	0.778 - 0.935	0.900	0.774 - 1.00
LRG1_042	0.864	0.785 - 0.944	0.917	0.780 - 1.00
CLEC3B_020	0.842	0.757 - 0.927	0.833	0.622 - 1.00
COMP_023	0.845	0.764 - 0.927	0.950	0.867 - 1.00
MRC1_073	0.768	0.670 - 0.865	0.854	0.676 - 1.00
F9_027	0.642	0.525 - 0.759	0.608	0.352 - 0.865
7-Panel	0.983	0.963 - 1.00	0.975	0.920 - 1.00

Supplementary Table 9. Validation cohort (Prague), AAV patients: demographic and clinical characteristics.

	Diagnosis			
	**active PR3-AAV**	**rem. PR3-AAV**	**active MPO-AAV**	**rem. MPO-AAV**
n	26	27	28	27
Age -year	61.3±9.8	60.7±9.8	62.9±11.2	60.9±11.6
Female -no. (%)	11 (42)	12 (44)	9 (32)	11 (41)
Female -no. (%)	15 (58)	15 (56)	19 (68)	16 (59)
**Disease entity**				
GPA	13	13	5	6
MPA	13	14	22	20
EGPA	0	0	1	1
**Clinical parameters**				
BVAS (0-63)	16.8±3.5	-	16.2±5.1	-
ANCA titer (CLIA, CU)	1404±1070<br>●<0.001<br>*<0.001	335±865<br>*<0.001	377±249<br>●<0.001<br>*<0.001	129±206<br>*<0.001
CRP (mg/L)	72.6±66.2<br>●0.011<br>*<0.001	4.3±6.3<br>*<0.001	32.4±41.9<br>●<0.011<br>*0.001	3.1±3.1<br>*0.001
Hematuria (sediment, Ery/µL)	407±538<br>●0.024<br>*0.001	13±27<br>*0.001	148±127<br>●0.024<br>*<0.001	4±5<br>*<0.001
Creatinine (mg/mL)	4.0±2.6<br>*0.002	2.0±1.7<br>*0.002	3.0±2.0	2.1±1.8
Hemoglobin (g/dL)	10.1±1.5<br>*<0.001	12.0±2.3<br>*<0.001	11.0±2.0<br>*0.003	12.5±1.7<br>*0.003
Hematocrit (%)	30.6±4.4<br>*<0.001	36.8±6.8<br>*<0.001	33.2±6.0<br>*0.002	38.1±5.2<br>*0.002
eGFR (ml/min/1,73 m²)	22.3±16.1.0<br>*<0.001	44.7±22.4<br>*<0.001	31.4±23.2<br>*0.027	45.9±23.6<br>*0.027
Leukocytes (/nL)	12.5±5.9<br>●0.006<br>*<0.001	6.5±1.7<br>*<0.001	8.8±2.8<br>●0.006<br>*0.007	6.8±2.4<br>*0.007
Neutrophils (/nL)	10.6±5.7<br>●0.005<br>*<0.001	4.7±1.5<br>*<0.001	7.0±2.7<br>●0.005<br>*0.006	5.0±2.3<br>*0.006
Platelets (/nL)	357±127<br>*<0.001	253±70<br>*<0.001	344±123<br>*0.002	254±66<br>*0.002
**Organ involvement**				
Kidney -no. (%)	26 (100)	-	28 (100)	-
Lung -no. (%)	7 (27)	-	9 (32)	-
Ear/Nose/Throat -no. (%)	9 (35)	-	5 (18)	-
Muscle/Joints -no. (%)	17 (65)	-	8 (29)	-
Skin/Mouth/Eyes -no. (%)	-	-	-	-
Central nervous system -no. (%)	-	-	2 (7)	-
**Induction Treatment**				

Naïve – no. (%)	8 (31)	-	13 (46)	-
Glucocorticoid (GC) -no. (%)	13 (50)	-	8 (29)	-
GC + Cyclophoshamide or rituximab -no. (%)	5 (19)	-	7 (25)	-
**Maintenance Treatment**				
Months in remission at sampling		7.8±13.2		4.9±2.7
Naïve – no. (%)	-	0 (0)	-	1 (4)
Glucocorticoid (GC) – no. (%)	-	26 (96)	-	26 (96)
Mean Glucocorticoid dose (mg/d)	-	5.2±1.9		5.6±1.9
RTX – no. (%)	-	1 (4)	-	1 (4)
AZA – no. (%)	-	18 (67)	-	16 (59)
MMF – no. (%)	-	7 (26)	-	9 (33)
Off-treatment – no. (%)#	-	0 ()	-	0 ()
On-treatment – no. (%)	-	27 (50)	-	27 (50)
Unknown – no. (%)	-	0 ()	-	0 ()
**Follow-up (24 month)**	-	27	-	27
Flare events – no. (%)	-	3 (11)	-	2 (7)
minor flares – no. (%)	-	2 (7)	-	1 (4)
major flares – no. (%)	-	1 (4)	-	1 (4)
Sustained rem. – no. (%)§		22 (81)		22 (81)
Unknown – no. (%)	-	2 (7)	-	3 (11)

Data are presented as no or mean±SD. (#) Off-treatment is defined as patients with less than 7.5 mg/d prednisolone and no other immunosuppressant currently or within the last 6 months. (§) Sustained remission means flare-free within the 24-month follow-up. (●) Indicates a significant difference between active PR3-AAV and active MPO-AAV as measured by two-tailed Welch's t-test or Chi-squared test ($P < 0.05$); (*) indicates a significant difference between active AAV and their corresponding remission group as measured by two-tailed Welch's t-test or Chi-squared test ($P < 0.05$). Precise p values of significant comparisons are provided behind corresponding symbol.

Supplementary Table 10. TQL data, validation cohort (external, n=108), 7-protein GLM and Ridge (discovery-trained): discrimination, calibration and separation diagnostics.

Discrimination, calibration, and training-set stability metrics for the discovery trained 7-protein GLM and Ridge-penalised model evaluated on the independent external validation cohort (n=108).

Metric	Value
**Discovery cohort trained GLM → independent external validation cohort**	
Discrimination	
AUC (DeLong 95% CI)	0.938 (0.897–0.979)
Brier score (bootstrap 95% CI)	0.116 (0.072– 0.166)
Label-permutation p (full cohort, n = 2,000, one-tailed)	< 0.001
Calibration (external validation cohort)	
Calibration intercept (bootstrap 95% CI)	−0.132 (-1.012–0.493)
Calibration slope (bootstrap 95% CI)	0.440 (0.306–0.759)
Hosmer–Lemeshow p (one-tailed)	< 0.001
Mean predicted probability	0.535
Observed event rate (54/108)	0.50
Calibration-in-the-large (overestimation)	0.035
Separation diagnostics (discovery train cohort)	
Extreme fitted probabilities (< 0.001 or > 0.999)	53 (51%)
Events per variable (EPV)	7.29
**Discovery cohort trained ridged GLM → independent external validation cohort (post hoc)**	
Discrimination	
AUC (DeLong 95% CI)	0.947 (0.911–0.984)
Brier score (bootstrap 95% CI)	0.105 (0.078– 0.134)
Label-permutation p (full cohort, n = 2,000, one-tailed)	< 0.001
Calibration (external validation cohort)	
Calibration intercept (bootstrap 95% CI)	-0.198 (-1.401 – 0.472)
Calibration slope (bootstrap 95% CI)	1.698 (1.165–3.244)
Hosmer–Lemeshow p (one-tailed)	0.291
Mean predicted probability	0.495
Observed event rate (54/108)	0.50
Calibration-in-the-large (overestimation)	-0.005
Separation diagnostics (discovery train cohort)	
Extreme fitted probabilities (< 0.001 or > 0.999)	1 (1%)
Events per variable (EPV)	7.29

AUC = area under the ROC curve (DeLong method). Bootstrap CIs: percentile method, 2,000 resamples, seed = 7. Calibration metrics computed on the external validation cohort (Prague, n = 108). Separation diagnostics (extreme fitted probabilities, EPV) refer to the Berlin discovery training set (n = 104), not the validation cohort. EPV = events per variable (guideline ≥ 10); extreme fitted probabilities defined as predicted probability < 0.001 or > 0.999. Ridge model: L2-penalised logistic regression, alpha = 0, lambda selected by 10-fold CV (lambda.1se). The Ridge model was selected after inspecting external validation calibration metrics and is therefore reported as a post-hoc sensitivity analysis; the Prague cohort does not constitute independent validation for this model.

Supplementary Table 11. TQL data, discovery training set (full, n=104), 7-protein GLM vs Ridge: coefficient comparison (separation-driven inflation).

GLM versus Ridge coefficient comparison for the 7-protein panel fitted on the full discovery training set (n = 104). The |GLM| / |Ridge| ratio quantifies separation-driven coefficient inflation.

Protein	GLM coefficient	Ridge coefficient	|GLM| / |Ridge|
**MCAM_047**	5.279	0.646	8.17×
**AHSG_001**	3.273	0.675	4.85×
**MRC1_073**	−3.688	−0.765	4.82×
**LRG1_042**	−2.954	−0.614	4.81×
**F9_027**	−3.219	−1.071	3.01×
**CLEC3B_020**	2.210	0.743	2.97×
**COMP_023**	−0.364	0.443	0.82×

Supplementary Table 12. TQL data, validation cohort (n=108, 54 events), 7-protein GLM vs CRP/ANCA ± combined: discrimination and threshold metrics (0.5 cut-off).

Discrimination of the 7-protein GLM panel compared with CRP alone, ANCA alone, and combined models in the independent external validation cohort (n = 108, 54 events). Threshold-dependent metrics at the pre-specified 0.5 operating threshold with Clopper–Pearson 95% CIs.

Model	n	Events	AUC (95% CI)	Sens (95% CI)	Spec (95% CI)	PPV (95% CI) *	NPV (95% CI) *	TP	TN	FP	FN
**GLM 7-protein panel**	108	54	0.938 (0.897–0.979)	0.852 (0.729–0.934)	0.796 (0.665–0.894)	0.807 (0.681–0.900)	0.843 (0.714–0.930)	46	43	11	8
CRP alone	108	54	0.908 (0.855–0.961)	0.981 (0.901–1.000)	0.630 (0.487–0.757)	0.726 (0.609–0.824)	0.971 (0.851–0.999)	53	34	20	1
ANCA alone	108	54	0.874 (0.804–0.944)	0.870 (0.751–0.946)	0.611 (0.469–0.741)	0.691 (0.567–0.798)	0.825 (0.672–0.927)	47	33	21	7
7-protein + CRP	108	54	0.940 (0.900–0.980)	0.889 (0.774–0.958)	0.796 (0.665–0.894)	0.814 (0.691–0.903)	0.878 (0.752–0.954)	48	43	11	6
7-protein + ANCA	108	54	0.942 (0.904–0.981)	0.852 (0.729–0.934)	0.796 (0.665–0.894)	0.807 (0.681–0.900)	0.843 (0.714–0.930)	46	43	11	8
7-protein + CRP + ANCA	108	54	0.942 (0.903–0.981)	0.889 (0.774–0.958)	0.759 (0.624–0.865)	0.787 (0.663–0.881)	0.872 (0.743–0.952)	48	41	13	6

*AUC = area under the ROC curve (DeLong 95% CI). Sens = sensitivity; Spec = specificity; PPV = positive predictive value; NPV = negative predictive value. All threshold metrics at 0.5 cut-off with exact Clopper–Pearson 95% CIs. TP = true positive; TN = true negative; FP = false positive; FN = false negative. * PPV and NPV depend on the absolute predicted probabilities and should be interpreted with caution given the poor cross-cohort calibration of the GLM (slope = 0.44); sensitivity and specificity are less affected as they reflect rank ordering at the 0.5 threshold, which remains meaningful given the confirmed discrimination (AUC = 0.938).*

Supplementary Table 13. TQL data, validation cohort, 7-protein GLM: discrimination by eGFR and treatment subgroup.

Discrimination of the 7-protein GLM panel between eGFR low and eGFR high patients in the independent external validation cohort (n = 108, 54 events). Threshold-dependent metrics at the pre-specified 0.5 operating threshold with Clopper–Pearson 95% CIs.

Model	n	Events	AUC (95% CI)	Sens (95% CI)	Spec (95% CI)	PPV (95% CI) *	NPV (95% CI) *	TP	TN	FP	FN
eGFR ≤ 45	72	27	0.942 (0.894–0.991)	0.815 (0.619–0.937)	0.822 (0.679–0.920)	0.733 (0.541–0.877)	0.881 (0.744–0.960)	22	37	8	5
eGFR > 45	36	27	0.889 (0.767–1.00)	0.926 (0.757–0.991)	0.667 (0.299–0.925)	0.893 (0.718–0.977)	0.750 (0.349–0.968)	25	6	3	2
Naïve vs GC	75	21	0.922 (0.864–0.981)	0.870 (0.751– 0.946)	0.714 (0.478 – 0.887)	0.887 (0.77 – 0.957)	0.682 (0.451 – 0.861)	47	15	6	7
Naïve vs GC + CYC + RTX	75	21	0.956 (0.906–1.00)	0.870 (0.751– 0.946)	0.857 (0.637–0.970)	0.940 (0.835–0.987)	0.720 (0.506–0.879)	47	18	3	7
GC vs GC + CYC + RTX	66	12	0.934 (0.860–1.00)	0.870 (0.729–0.934)	0.833 (0.516–0.979)	0.959 (0.86–0.995)	0.588 (0.329–0.816)	47	10	2	7

Supplementary Table 14. TQL data, validation cohort, 7-protein GLM vs comparators: DeLong AUC comparison (ΔAUC and p-values).

DeLong pairwise AUC comparisons for the 7-protein GLM panel versus clinical comparators in the independent external validation cohort (n = 108, 54 events). Reference model: 7-protein GLM panel (AUC = 0.938).

Comparison	AUC (panel)	AUC (comparator)	ΔAUC (95% CI)	p-value (DeLong), two-tailed
7-protein panel (reference)	0.938	—	—	—
vs CRP alone	0.938	0.908	+0.030 (-0.019 – 0.079)	0.234
vs ANCA alone	0.938	0.874	+0.064 (-0.011 - 0.148)	0.198
vs 7-protein + CRP	0.938	0.940	−0.002 (-0.011 – 0.006)	0.574
vs 7-protein + ANCA	0.938	0.942	-0.004 (-0.012 - 0.004)	0.313
vs 7-protein + CRP + ANCA	0.938	0.942	−0.004 (-0.014 – 0.005)	0.394

ΔAUC = AUC of 7-protein panel minus AUC of comparator; positive values favour the panel. The panel numerically outperforms CRP alone (ΔAUC = +0.030) but the difference does not reach statistical significance (p = 0.234), consistent with overlapping confidence intervals. Adding CRP or ANCA to the panel provides no incremental benefit (all p > 0.30).

Supplementary Table 15. TQL data, validation cohort, 7-protein GLM: AUC comparison, eGFR ≤45 vs >45 mL/min/1.73m².

DeLong test comparing 7-protein GLM AUC between eGFR strata in the external validation cohort. eGFR ≤ 45 vs > 45 ml/min/1.73m²

Comparison	AUC — stratum 1	AUC — stratum 2	ΔAUC (95% CI)	p-value (DeLong), two-tailed
eGFR ≤ 45 vs eGFR > 45	0.942	0.889	+0.053 (−0.081 – 0.188)	0.429

ΔAUC = AUC in eGFR ≤ 45 stratum minus AUC in eGFR > 45 stratum. The difference in AUC between eGFR strata is not statistically significant (p = 0.429). D = DeLong test statistic.

Supplementary Table 16. TQL data, validation cohort, 7-protein GLM: AUC comparison across treatment strata (Naïve/GC/GC+CYC+RTX).

DeLong pairwise AUC comparisons between treatment subgroups in the external validation. Treatment strata: Naïve, GC, GC + CYC + RTX.

Comparison	AUC — group 1	AUC — group 2	ΔAUC (95% CI)	p-value(deLong), two-tailed
Naïve vs GC	0.922	0.956	−0.034 (−0.111 – 0.029)	0.395
Naïve vs GC + CYC + RTX	0.922	0.933	-0.011 (−0.105 – 0.091)	0.816
GC vs GC + CYC + RTX	0.955	0.934	+0.022(−0.063 – 0.118)	0.627

Naïve = no immunosuppressive treatment; GC = glucocorticoids; GC + CYC + RTX = glucocorticoids plus cyclophosphamide or rituximab. No statistically significant difference in AUC was detected between any treatment pair (all p > 0.39), indicating consistent discrimination regardless of treatment background.

Supplementary Table 17. TQL data, validation cohort (post hoc), Ridge 7-protein panel vs CRP/ANCA: discrimination and threshold metrics (0.5 cut-off).

Discrimination of the Ridge-penalised 7-protein panel compared with clinical comparators in the independent external validation cohort (n = 108) — post-hoc sensitivity analysis. Threshold-dependent metrics at the 0.5 operating threshold with Clopper–Pearson 95% CIs.

Model	n	Events	AUC (95% CI)	Sens (95% CI)	Spec (95% CI)	PPV (95% CI)	NPV (95% CI)	TP	TN	FP	FN
**Ridge 7-protein panel**	108	54	0.947 (0.911–0.984)	0.852 (0.729–0.934)	0.796 (0.665–0.894)	0.807 (0.681–0.900)	0.843 (0.714–0.930)	46	43	11	8
CRP alone	108	54	0.908 (0.855–0.961)	0.981 (0.901–1.000)	0.630 (0.487–0.757)	0.726 (0.609–0.824)	0.971 (0.851–0.999)	53	34	20	1
ANCA alone	108	54	0.874 (0.804–0.944)	0.870 (0.751–0.946)	0.611 (0.469–0.741)	0.691 (0.567–0.798)	0.825 (0.672–0.927)	47	33	21	7
7-protein + CRP	108	54	0.949 (0.914–0.985)	0.870 (0.751–0.946)	0.815 (0.686–0.907)	0.825 (0.701–0.913)	0.863 (0.737–0.943)	47	44	10	7
7-protein + ANCA	108	54	0.948 (0.912–0.984)	0.833 (0.707–0.921)	0.796 (0.665–0.894)	0.804 (0.676–0.898)	0.827 (0.697–0.918)	45	43	11	9
7-protein + CRP + ANCA	108	54	0.951 (0.916–0.986)	0.889 (0.774–0.958)	0.815 (0.686–0.907)	0.828 (0.706–0.914)	0.880 (0.757–0.955)	48	44	10	6

Supplementary Table 18. TQL data, pooled cohort (Train60→Test40, n=85), 7-protein panel: discrimination, calibration and separation diagnostics.

Discrimination, calibration, and training-set stability metrics for the 7-protein TQL normalized intensity model evaluated on the pooled 60/40 held-out test set (Test40, n = 85). Calibration metrics are computed on Test40. Separation diagnostics and EPV refer to the Train60 training set (n = 127). Bootstrap CIs: percentile method, 2,000 resamples, seed = 7.

Metric	Value
**Pooled cohort 60Train → 40Test**	
Discrimination	
AUC (DeLong 95% CI)	0.974 (0.948 - 1.00)
Brier score (bootstrap 95% CI)	0.067 (0.033 - 0.109)
Label-permutation p (full cohort, n = 2,000, one-tailed)	< 0.001
Calibration (held-out test set (Test40))	
Calibration intercept (bootstrap 95% CI)	−0.436 (- 1.951 - 0.498)
Calibration slope (bootstrap 95% CI)	1.029 (0.739 – 1.988)
Hosmer–Lemeshow p (one-tailed)	0.667
Mean predicted probability	0.532
Observed event rate (43/85)	0.506
Calibration-in-the-large (overestimation)	0.026
Separation diagnostics	
Extreme fitted probabilities (< 0.001 or > 0.999)	23 (29%)
Events per variable (EPV)	8.86

AUC = area under the ROC curve (DeLong method). EPV = events per variable (guideline ≥ 10); extreme fitted probabilities defined as predicted probability < 0.001 or > 0.999 in the Train60 training set. The 60/40 split was defined prior to model fitting on the pooled discovery and validation cohort. The observed event rate of 0.506 in Test40 reflects the composition of the pooled test set (Berlin: 39% remission; Prague: 61% remission) and does not represent either cohort's true prevalence.

Supplementary Table 19. TQL data, pooled cohort, 7-protein panel: actual vs predicted median ratios by disease state.

Protein	Actual	Median TQL ratio	Predicted	Median TQL ratio
**AHSG**	Active	2,663	Active	2,567
	Remission	3,558	Remission	3,544
**CLEC3B**	Active	1,709	Active	1,630
	Remission	2,345	Remission	2,317
**COMP**	Active	-4,527	Active	-4,707
	Remission	-3,959	Remission	-3,950
**F9**	Active	-0,519	Active	-0,506
	Remission	-0,896	Remission	-0,892
**LRG1**	Active	3,192	Active	3,295
	Remission	2,474	Remission	2,474
**MCAM**	Active	-4,798	Active	-4,868
	Remission	-4,111	Remission	-4,105
**MRC1**	Active	-7,807	Active	-7,668
	Remission	-8,242	Remission	-8,194

Supplementary Table 20. TQL data, pooled Test40 (n=85), 7-protein panel: discrimination by ANCA specificity (MPO vs PR3).

Discrimination of all models stratified by ANCA specificity (all: n=85, 43 events; MPO-ANCA: n = 37, 17 events; PR3-ANCA: n = 48, 26 events) in the pooled 60/40 held-out test set (Test40). Threshold-dependent metrics at the 0.5 operating threshold with Clopper–Pearson 95% CIs.

ANCA Subgroup	Model	n	Events	AUC (95% CI)	Sens (95% CI)	Spec (95% CI)	PPV (95% CI)	NPV (95% CI)
**MPO**	**7-protein panel**	37	17	0.953 (0.891–1.000)	0.941 (0.713–0.999)	0.750 (0.509–0.913)	0.762 (0.528–0.918)	0.938 (0.698–0.998)
**PR3**	**7-protein panel**	48	26	1.000 (1.000–1.000) ‡	0.962 (0.804–0.999)	1.000 (0.846–1.000)	1.000 (0.863–1.000)	0.957 (0.781–0.999)
**all**	**7-protein panel**	85	43	0.974 (0.948–1.000)	0.953 (0.842–0.994)	0.881 (0.744–0.960)	0.891 (0.764–0.964)	0.949 (0.827–0.994)

AUC = area under the ROC curve (DeLong 95% CI). Sens = sensitivity; Spec = specificity; PPV = positive predictive value; NPV = negative predictive value. Clopper–Pearson 95% CIs throughout. MPO = myeloperoxidase-ANCA; PR3 = proteinase 3-ANCA. The 7-protein panel and augmented models were fit on Train60 and applied once to Test40; subgroup analyses were pre-specified. ‡ AUC = 1.000 in the PR3 subgroup indicates perfect rank separation of remission from active disease in this subset (n = 48). This is a small subgroup and the result should be interpreted with caution; wide Clopper–Pearson CIs on threshold metrics reflect the limited sample size. DeLong 95% CI collapses to a point estimate at perfect discrimination and is uninformative; bootstrap CIs are not reported as they are similarly degenerate at AUC = 1.000.

Supplementary Table 21. TQL data, pooled Test40 (n=85), 7-protein panel vs CRP/ANCA ± combined: discrimination and threshold metrics (0.5 cut-off).

Discrimination of the 7-protein TQL normalised intensity panel compared with CRP alone, ANCA alone, and combined models in the pooled 60/40 held-out test set (Test40, n = 85, 43 events). Threshold-dependent metrics at the pre-specified 0.5 operating threshold with Clopper–Pearson 95% CIs.

Model	n	Events	AUC (95% CI)	Sens (95% CI)	Spec (95% CI)	PPV (95% CI) *	NPV (95% CI) *	TP	TN	FP	FN
**7-protein panel**	85	43	0.974 (0.948–1.000)	0.953 (0.842–0.994)	0.881 (0.744–0.960)	0.891 (0.764–0.964)	0.949 (0.827–0.994)	41	37	5	2
CRP alone	85	43	0.932 (0.880–0.984)	1.000 (0.918–1.000)	0.690 (0.529–0.824)	0.768 (0.636–0.870)	1.000 (0.881–1.000)	43	29	13	0
ANCA alone	85	43	0.746 (0.635–0.857)	0.767 (0.614–0.882)	0.643 (0.480–0.784)	0.688 (0.537–0.813)	0.730 (0.559–0.862)	33	27	15	10
CRP + ANCA	85	43	0.893 (0.826–0.959)	0.860 (0.721–0.947)	0.690 (0.529–0.824)	0.740 (0.597–0.854)	0.829 (0.664–0.934)	37	29	13	6
7-protein + CRP	85	43	0.973 (0.946–1.000)	0.953 (0.842–0.994)	0.881 (0.744–0.960)	0.891 (0.764–0.964)	0.949 (0.827–0.994)	41	37	5	2
7-protein + ANCA	85	43	0.957 (0.920–0.994)	0.884 (0.749–0.961)	0.881 (0.744–0.960)	0.884 (0.749–0.961)	0.881 (0.744–0.960)	38	37	5	5
7-protein + CRP + ANCA	85	43	0.959 (0.923–0.995)	0.884 (0.749–0.961)	0.881 (0.744–0.960)	0.884 (0.749–0.961)	0.881 (0.744–0.960)	38	37	5	5

AUC = area under the ROC curve (DeLong 95% CI). Sens = sensitivity; Spec = specificity; PPV = positive predictive value; NPV = negative predictive value. All threshold metrics at 0.5 cut-off with exact Clopper–Pearson 95% CIs. TP = true positive; TN = true negative; FP = false positive; FN = false negative. The 60/40 split was defined prior to model fitting; augmented models (7-protein + CRP/ANCA) were fit on Train60 and applied once to Test40. ******* *PPV and NPV depend on the absolute predicted probabilities. The 7-protein panel shows good calibration in Test40 (slope = 1.03), nonetheless they reflect the observed Test40 event rate (43/85 = 0.506), which is an artefact of pooling Berlin (39% remission) and Prague (61% remission) and does not represent either cohort's true prevalence.*

Supplementary Table 22. TQL data, pooled Test40, 7-protein panel vs comparators: DeLong AUC comparison (ΔAUC and p-values).

DeLong pairwise AUC comparisons for the 7-protein GLM panel versus clinical comparators in the pooled 60/40 held-out test set (Test40, n = 85, 43 events). Reference model: 7-protein GLM panel (AUC = 0.974).

Comparison	AUC (panel)	AUC (comparator)	ΔAUC (95% CI)	p-value (DeLong, one-tailed)
7-protein panel (reference)	0.974	—	—	—
vs CRP alone	0.974	0.932	+0.042 (-0.013 – 0.105)	0.08
vs ANCA alone	0.974	0.746	+0.228 (0.121 - 0.346)	< 0.001
vs CRP + ANCA	0.974	0.893	+0.081 (0.015 - 0.159)	< 0.01
vs 7-protein + CRP	0.974	0.973	+0.001 (-0.039 - 0.042)	0.717
vs 7-protein + ANCA	0.974	0.957	+0.017(-0.028 - 0.066)	0.108
vs 7-protein + CRP + ANCA	0.974	0.959	+0.014 (-0.029 - 0.063)	0.129

ΔAUC = AUC of 7-protein panel minus AUC of comparator; positive values favour the panel. 95% CI estimated by stratified bootstrap (2,000 resamples, seed = 7). p-value from DeLong test. The panel significantly outperforms ANCA alone (ΔAUC = +0.228, p < 0.001) and CRP + ANCA combined (ΔAUC = +0.081, p < 0.01). The difference versus CRP alone does not reach statistical significance (ΔAUC = +0.042, p = 0.234), consistent with overlapping confidence intervals and the high AUC of CRP alone in this cohort. Adding CRP or ANCA to the panel provides no incremental benefit (all p > 0.10).

Supplementary Table 23. TQL data, pooled Test40, 7-protein panel vs CRP: AUC stratified by ANCA/CRP status plus likelihood ratios.

CRP- and ANCA-stratified AUC comparison: 7-protein panel vs CRP alone in Test40 (total n = 85).

Subgroup	N (subgroup)	Events AAV-A	Events AAV-R	Panel AUC (95% CI)	CRP AUC (95% CI)	ΔAUC (95% CI)	p-value (DeLong, two-tailed)	IDI (95% CI)
ANCA positive	73	42	31	0.973 (0.940 – 0.995)	0.936 (0.875–0.980)	+0.037 (-0.005–0.094)	0.072	+0.255 (0.156–0.350)
CRP > 5 mg/L, ANCA positive	42	37	5	0.968 (0.893 – 1.000)	0.878 (0.737–0.980)	+0.089 (-0.042–0.232)	0.087	+0.285 (-0.109–0.520)
CRP > 5 mg/L	46	37	9	0.973 (0.925–1.000)	0.908 (0.811–0.979)	+0.065 (-0.012–0.165)	0.069	+0.263 (0.060–0.439)
All	85	42	43	0.974 (0.942–0.994)	0.932 (0.873-0.976)	+0.042 (0.001–0.092)	0.039	+0.261 (0.174–0.354)

AAV-A = active disease (non-remission, group01 = 0); AAV-R = remission (group01 = 1). Panel AUC / CRP AUC: 95% confidence intervals (CI) estimated by bootstrap (2,000 resamples, seed = 7). ΔAUC = Panel AUC − CRP AUC; 95% CI derived from paired bootstrap differences. DeLong p-values: computed using *pROC::roc.test* (method = "DeLong"), one-sided tests evaluate H_1: Panel AUC > CRP AUC. IDI = Integrated Discrimination Improvement (Pencina et al., 2008). Positive IDI indicates higher predicted probabilities for events and lower probabilities for non-events for the panel relative to CRP. 95% CI estimated by bootstrap.

Subgroup	Panel LR+ (95% CI)	Panel LR− (95% CI)	CRP LR+ (95% CI)	CRP LR- (95% CI)
ANCA positive	7.86 (4.09–32.35)	0.073 (0–0.190)	3.23 (2.18–5.57)	~0 (unstable)
CRP > 5 mg/L, ANCA positive	14.80 (3.73–39.00)	0.211 (0–0.722)	4.63 (2.77–10.00)	~0 (unstable)
CRP > 5 mg/L	16.44 (6.00–39.78)	0.117 (0–0.374)	4.63 (2.77–10.25)	~0 (unstable)
All	8.01 (4.24–33.33)	0.053 (0–0.141)	3.23 (2.21–5.57)	~0 (unstable)

Likelihood ratios (LR+ / LR−): derived from sensitivity and specificity at threshold = 0.5. LR+ = sensitivity / (1 − specificity); LR− = (1 − sensitivity) / specificity. 95% CIs estimated by bootstrap; values may be unstable in small subgroups. Note: subgroups with ≤50 patients are underpowered and should be interpreted as exploratory. The CRP > 5 mg/L subgroup contains only 9 remission events (AAV-R), resulting in increased uncertainty of estimates. ANCA-negative patients (n = 12): all were in remission; discrimination analyses were not applicable, and these patients were excluded from subgroup comparisons.

Supplementary Table 24. TQL data, pooled Test40 (n=85), 7-protein panel vs CRP alone: decision curve analysis — ΔNB by threshold.

Paired net benefit difference (ΔNB = panel − CRP alone) at key decision thresholds in the pooled Test40 set (n = 85). Bootstrap 95% CIs, percentile method, 1,000 resamples.

Threshold	NB panel	NB CRP alone	ΔNB	95% CI low	95% CI high
0.15	0.483	0.464	+0.019	0.006	0.033
0.20	0.476	0.447	+0.029	0.012	0.050
0.25	0.475	0.431	+0.043	0.020	0.071
0.26	0.473	0.427	+0.045	0.025	0.074
0.30	0.442	0.410	+0.032	−0.017	0.076
0.40	0.427	0.380	+0.047	−0.004	0.102

NB = net benefit per patient. Threshold = minimum probability of remission required to recommend tapering. Panel score = frozen predicted probability from Train60 7-protein GLM. CRP score = logistic wrapper fitted on Train60. ΔNB at thresholds 0.15–0.26 consistently favours the panel with CIs excluding zero. At thresholds ≥0.27 CIs include zero, reflecting reduced power where fewer patients reach the tapering threshold.

Supplementary Table 25. TQL data, pooled Test40, 7-protein panel vs ANCA alone: decision curve analysis — ΔNB by threshold.

Paired net benefit difference (ΔNB = panel − ANCA alone) at key decision thresholds in the pooled Test40 set (n = 85). Bootstrap 95% CIs, percentile method, 1,000 resamples, seed = 7.

Threshold	NB panel	NB ANCA alone	ΔNB	95% CI low	95% CI high
0.15	0.483	0.375	+0.108	0.057	0.165
0.20	0.476	0.347	+0.129	0.071	0.191
0.25	0.475	0.341	+0.133	0.074	0.200
0.26	0.473	0.337	+0.136	0.075	0.202
0.30	0.442	0.311	+0.131	0.049	0.210
0.40	0.427	0.255	+0.173	0.082	0.259

ANCA score = logistic wrapper fitted on Train60 using zANCA as sole predictor. The panel advantage over ANCA alone is 3–5× larger than over CRP alone at every threshold (ΔNB +0.108–+0.173 vs +0.019–+0.047), reflecting the substantially lower discriminative ability of ANCA titer for remission classification in this cohort. Critically, CIs exclude zero across the entire clinically relevant range (0.15–0.40), confirming that the panel advantage over ANCA is statistically robust throughout. This contrasts with CRP (Table 16a), where the panel advantage is smaller but still significant at thresholds 0.15–0.26, highlighting that CRP remains a stronger single-marker comparator than ANCA for this task.

Supplementary Table 26. **TQL data, pooled Test40, 7-protein panel vs ANCA+CRP: decision curve analysis — ΔNB by threshold.**

Paired net benefit difference (ΔNB = panel − ANCA+CRP) at key decision thresholds — pooled Test40 set (n = 85).
Bootstrap 95% CIs, percentile method, 1,000 resamples, seed = 7. All rows green: CI excludes zero at every bootstrapped threshold. ANCA+CRP score = logistic wrapper fitted on Train60 using both zANCA and CRP_Plasma_mg_L.

Threshold	NB panel	NB ANCA+CRP	ΔNB	95% CI low	95% CI high
0.15	0.483	0.408	**+0.075**	0.032	0.129
0.20	0.476	0.394	**+0.082**	0.035	0.141
0.25	0.475	0.369	**+0.106**	0.051	0.169
0.26	0.473	0.365	**+0.108**	0.051	0.166
0.30	0.442	0.355	**+0.087**	0.015	0.161
0.40	0.427	0.333	**+0.094**	0.016	0.169

† Threshold 0.26 was not included in THRESH_REPORT; point estimate shown, bootstrap CI not available. The panel advantage over ANCA+CRP (ΔNB +0.075–+0.106 across 0.15–0.25) is intermediate between its advantage over CRP alone (+0.019–+0.043) and over ANCA alone (+0.108–+0.133), consistent with ANCA+CRP being marginally worse than CRP alone for this decision task. CIs exclude zero at all five bootstrapped thresholds (0.15–0.40), confirming a robust panel advantage throughout the clinically relevant range.

Supplementary Table 27. TQL data, Test40 by cohort subset, 7-protein panel vs CRP/ANCA/ANCA+CRP: cohort-specific net benefit difference.

Cohort-specific ΔNB in discovery and validation cohort subsets of Test40.
Paired bootstrap 95% CIs, 1,000 resamples, seed = 7. Green = CI excludes zero. Panel − ANCA+CRP column: see Table 16e for full bootstrap details.

Cohort	Thres-hold	ΔNB (panel−CRP)	CI low	CI high	ΔNB (panel−ANCA)	CI low	CI high	ΔNB (panel−ANCA+CRP)	CI low	CI high
**discovery**	0.15	**+0.022**	0.000	0.043	**+0.149**	0.067	0.254	**+0.099**	0.030	0.188
	0.20	**+0.030**	0.000	0.061	**+0.189**	0.098	0.287	**+0.110**	0.037	0.207
	0.25	**+0.049**	0.016	0.089	**+0.187**	0.081	0.293	**+0.154**	0.065	0.252
	0.30	**+0.038**	−0.038	0.105	**+0.181**	0.063	0.310	**+0.136**	0.031	0.251
	0.40	**+0.057**	−0.033	0.138	**+0.244**	0.098	0.381	**+0.138**	0.024	0.260
**validation**	0.15	**+0.016**	0.004	0.032	**+0.070**	0.016	0.128	**+0.053**	0.004	0.122
	0.20	**+0.028**	0.006	0.051	**+0.074**	0.017	0.153	**+0.057**	0.006	0.125
	0.25	**+0.038**	0.008	0.068	**+0.083**	0.022	0.152	**+0.061**	0.008	0.136
	0.30	**+0.026**	−0.036	0.078	**+0.083**	−0.003	0.175	**+0.042**	−0.035	0.133
	0.40	**+0.038**	−0.038	0.114	**+0.106**	0.001	0.212	**+0.053**	−0.030	0.144

Discovery subset: n = 41, 16 remission events (39% prevalence). Prague (validation) subset: n = 44, 27 remission events (61% prevalence). Cohort interaction test (group01 ~ protein_score × cohort): interaction coefficient = −0.897, z = −0.321, p = 0.748; no evidence of effect modification by cohort; pooling is statistically defensible. The panel advantage is consistent in direction across both cohorts at thresholds 0.15–0.25, although CIs in the discovery cohort are wider due to lower event count.

Supplementary Table 28. TQL data, pooled Test40, 7-protein panel, CRP, ANCA, ANCA+CRP: net benefit by threshold (decision-curve values).

Net benefit of the 7-protein panel, CRP alone, and ANCA alone at key decision thresholds in Test40 (n = 85). Panel = protein score. CRP and ANCA = logistic wrapper scores fitted on Train60.

Threshold	Treat all	7-protein panel	CRP alone	ANCA + CRP	ANCA alone	Treat none
0.15	0.419	0.483	0.464	**0.408**	0.375	0.000
0.20	0.382	0.476	0.447	**0.394**	0.347	0.000
0.25	0.341	0.475	0.431	**0.369**	0.341	0.000
0.30	0.294	0.442	0.410	**0.355**	0.311	0.000
0.35	0.240	0.438	0.398	**0.340**	0.285	0.000
0.40	0.176	0.428	0.380	**0.333**	0.255	0.000
0.45	0.102	0.434	0.362	**0.310**	0.236	0.000
0.50	0.012	0.424	0.353	**0.282**	0.212	0.000

The 7-protein panel provides higher net benefit than both CRP alone and ANCA alone at all evaluated thresholds. The advantage over ANCA is large and consistent across the full range. The advantage over CRP is smaller but statistically confirmed at thresholds 0.15–0.26 (see Table DCA-1). Treat all = taper every patient regardless of model; Treat none = taper no patient. Both are reference strategies with no model input.

Supplementary Table 29. 24-month follow-up, pooled cohort (n=96), 7-protein score (pre-trained, no refit): flare/relapse prediction — AUC, Cox, KM.

Analysis	N patients	N flares (24mo)	AUC (95% CI)	p-value
7-protein score vs. flare within 24 months	96	15 (16%)	0.44 (0.29–0.60)	—
Cox regression (per 0.1 increase in protein score)	96	15 (16%)	HR 0.92 (0.76–1.11)	0.49
KM log-rank (median split, illustration only)	96	15 (16%)	—	0.991

AUC = area under the ROC curve; OR = odds ratio from logistic regression; KM = Kaplan–Meier. All analyses applied the pre-trained model; no refitting was performed.

Supplementary Table 30. Concentration data, pooled cohort (Test40), 7-protein panel, absolute concentration (VSN): discrimination, calibration and separation diagnostics.

Discrimination, calibration, and training-set stability metrics for the 7-protein absolute concentration model evaluated on the pooled 60/40 held-out test set (Test40, n = 84).

Metric	Value
**Pooled cohort concentration 60Train → 40Test**	
Discrimination	
AUC (DeLong 95% CI, two-tailed)	0.908 (0.847 – 0.968)
Brier score (bootstrap 95% CI)	0.127 (0.080 - 0.177)
Label-permutation p (full cohort, n = 2,000, one-tailed)	< 0.001
Calibration (held-out test set (Test40))	
Calibration intercept (bootstrap 95% CI)	−0.099 (-0.867 - 0.526)
Calibration slope (bootstrap 95% CI)	0.804 (0.574 – 1.290)
Hosmer–Lemeshow p (one-tailed)	0.079
Mean predicted probability	0.531
Observed event rate (43/85)	0.512
Calibration-in-the-large (overestimation)	0.019
Separation diagnostics	
Extreme fitted probabilities (< 0.001 or > 0.999)	6 (5%)
Events per variable (EPV)	8.86

AUC = area under the ROC curve (DeLong method). EPV = events per variable (guideline ≥ 10); extreme fitted probabilities defined as predicted probability < 0.001 or > 0.999 in the Train60 training set. The concentration model uses absolute plasma protein concentrations converted from TQL-based PRM-MS signal intensities and subsequently variance-stabilisation normalised (VSN). The observed event rate of 0.506 in Test40 reflects the composition of the pooled test set (Berlin: 39% remission; Prague: 61% remission) and does not represent either cohort's true prevalence.

Supplementary Table 31. Concentration data, pooled Test40, 7-protein panel, absolute concentration (VSN): threshold metrics (0.5 cut-off).

Discrimination of the 7-protein absolute concentration model in the pooled 60/40 held-out test set (Test40, n = 84, 43 events). Threshold-dependent metrics at the pre-specified 0.5 operating threshold with Clopper–Pearson 95% CIs.

Model	n	Events	AUC (95% CI)	Sens (95% CI)	Spec (95% CI)	PPV (95% CI)	NPV (95% CI)	TP	TN	FP	FN
**7-protein panel**	85	43	0.908 (0.847–0.968)	0.860 (0.721–0.947)	0.756 (0.567–0.876)	0.787 (0.643–0.893)	0.838 (0.680–0.938)	37	31	10	6

AUC = area under the ROC curve (DeLong 95% CI). Sens = sensitivity; Spec = specificity; PPV = positive predictive value; NPV = negative predictive value. All threshold metrics at 0.5 cut-off with exact Clopper–Pearson 95% CIs. TP = true positive; TN = true negative; FP = false positive; FN = false negative. The concentration model uses absolute plasma protein concentrations converted from TQL-based PRM-MS signal intensities and subsequently variance-stabilisation normalised (VSN). PPV and NPV depend on absolute predicted probabilities; the concentration model shows the observed event rate in Test40 (43/84 = 0.512) reflects the composition of the pooled test set and does not represent either cohort's true prevalence, interpret PPV and NPV accordingly.

Supplementary Table 32. Reporting checklist, TRIPOD adherence: item-by-item development and external validation assessment.

Assessing adherence of prediction model reports to the TRIPOD guideline

This document provides guidance for extracting the relevant information and calculating summary scores to determine adherence of primary prediction model reports to the TRIPOD (Transparent Reporting of studies on prediction models for Individual Prognosis Or Diagnosis) reporting guideline (issued in January 2015; www.tripod-statement.org). **To be able to compare TRIPOD adherence evaluations, e.g. over time or over clinical domains, it is crucial that investigators use uniform methods, i.e. this adherence assessment form. If investigators decide to deviate from this form and scoring rules, they should be explicit and transparent about the changes they make.**

Extracting the data

This TRIPOD adherence assessment form consists of two parts. Part A is to extract general information from a publication about the development and/or validation of a diagnostic or prognostic prediction model, or about the assessment of the incremental value of one or more predictors on top of an existing prediction model. Part B lists all 22 main items of the original TRIPOD reporting guideline, of which ten were divided in sub items (denoted by a, b, c, etc.). Below, these are shaded in blue and further referred to as the TRIPOD items. To properly assess adherence of a study report to the TRIPOD reporting items, we further specified these TRIPOD items into multiple so-called adherence elements (denoted by i, ii, iii, …) simply because the original TRIPOD items often mentioned multiple elements to report. Accordingly, the form below provides a comprehensive tool to look for the information deemed necessary by the TRIPOD reporting guideline to judge the adherence of reports to this guideline.

There are four columns in which information can be entered: one for reports about the development of a prediction model [D], one for reports on external validation of a prediction model [V], one for reports on the incremental value of predictor(s) to an existing prediction model [IV], and one for reports on the development plus external validation of the same model [D+V]. If a report addresses both the development and validation of the same model, then both columns D and V should be used to assess the reporting of the development and external validation, and, subsequently, column D+V to combine the information of these two. If a report addresses the development of a model and external validation of a different model, one can use the columns D and V to assess the reporting however, information should not be combined using column D+V. For publications in which more than one (different) prediction model is developed or validated, scoring could be based on the model of interest (or most clearly reported model).
The adherence elements are formulated as statements, for which there are four potential answer options: yes (Y), no (N), referenced (R), and not applicable (NA). For some elements it may be acceptable if authors in their report specifically reference to another publication (i.e. explicitly mention that the

information of that adherence element is described somewhere else). This is denoted by the answer option “R”. For adherence elements that do not apply to a specific situation, there is the answer option “NA”.

Some TRIPOD items do not apply to all four types of prediction model studes, e.g. TRIPOD item 10a *“Describe how predictors were handled in the analyses”*, is not applicable when reporting about external validation, whereas TRIPOD item 10c *“For validation, describe how the predictions were calculated”* does not apply to the reporting of model development. In such instances we state ‘not applicable’ and grey shaded these adherence elements.

Calculating adherence to TRIPOD

First, adherence of a report is calculated per TRIPOD item. If the answer to all adherence elements of a particular TRIPOD item is scored “yes”, adherence to that TRIPOD item is scored as “1”, and non adherence as “0”. In some situations a different scoring rule is used, which is described in the adherence assessment form below for the corresponding items.

Subsequently, a report’s overall TRIPOD adherence score can be calculated. This is calculated by dividing the sum of the adhered TRIPOD items by the total number of applicable TRIPOD items for that report. This total can vary since some TRIPOD items may be not applicable to all four types of prediction model studies. The total number of applicable TRIPOD items for D studies is 30, for V 30, for D+V 36 and for IV 35.[1] In addition, five TRIPOD items (5c, 10e, 11, 14b, and 17) might not be applicable for specific reports.

If one reviews multiple prediction model studies on their adherence to TRIPOD, overall adherence per TRIPOD item can be calculated by dividing the number of studies that adhered to a specific TRIPOD item by the number of studies in which the specific TRIPOD item was applicable.

[1] TRIPOD item 21 is not taken into account in the overall score in any of the four types of studies.

A. GENERAL INFORMATION	
Study ID	
First author(s)	Uwe Jerke, Marieluise Kirchner, and Theda UP Bartolomaeus
Publication year	
Title	**Towards a proteomic plasma biomarker panel for diagnosing vasculitis remission**
Journal	**Nature Communication**
Diagnostic or prognostic prediction model?	X Diagnostic<br>☐ Prognostic
Type of prediction model study *(multiple options possible)*	x Development<br>x External validation<br>*If both development and external validation:*<br>x same model/score<br>☐ different models/scores<br>☐ Incremental value

B. TRIPOD ITEMS						
			[D] Development	[V] External validation	[IV] Incremental value	[D+V] Development and external validation (of same model)
**Title and abstract**<br>***It is suggested to score items 1 and 2 (Title and Abstract) after scoring items 3 to 22, as only after reading the whole publication it can be judged whether the reporting in the title and abstract is complete.***						
**Title**	**1**	**Identify the study as developing and/or validating a multivariable prediction model, the target population, and the outcome to be predicted.**	**Score 1 if all elements are scored as "Y"**	**Score 1 if all elements are scored as "Y"**	**Score 1 if all elements are scored as "Y"**	**Score 1 if all elements are scored as "Y"**
	i	The words developing/development, validation/validating, incremental/added value (or synonyms) are reported in the title				N
	ii	The words prediction, risk prediction, prediction model, risk models, prognostic models, prognostic indices, risk scores (or synonyms) are reported in the title				Y
	iii	The target population is reported in the title				Y
	iv	The outcome to be predicted is reported in the title				Y
**Abstract**	**2**	**Provide a summary of objectives, study design, setting, participants, sample size, predictors, outcome, statistical analysis, results, and conclusions.**	**Score 1 if all elements are scored as "Y" or "NA"**	**Score 1 if all elements are scored as "Y" or "NA"**	**Score 1 if all elements are scored as "Y" or "NA"**	**Score 1 if all elements are scored as "Y" or "NA"**
	i	The objectives are reported in the abstract				Y
	ii	Sources of data are reported in the abstract<br>*E.g. Prospective cohort, registry data, RCT data.*				Y
	iii	The setting is reported in the abstract<br>*E.g. Primary care, secondary care, general population, adult care, or paediatric care. The setting should be reported for both the development and validation datasets, if applicable.*				Y

	iv	A general definition of the study participants is reported in the abstract<br>*E.g. patients with suspicion of certain disease, patients with a specific disease, or general eligibility criteria.*				Y
	v	The overall sample size is reported in the abstract				Y
	vi	The number of events (or % outcome together with overall sample size) is reported in the abstract<br>*If a continuous outcome was studied, score Not applicable*				NA
	vii	Predictors included in the final model are reported in the abstract. For validation studies of well-known models, at least the name/acronym of the validated model is reported<br>*Broad descriptions are sufficient, e.g. 'all information from patient history and physical examination'.*<br>*Check in the main text whether all predictors of the final model are indeed reported in the abstract.*				Y
	viii	The outcome is reported in the abstract				Y
	ix	Statistical methods are described in the abstract<br>*For model development, at least the type of statistical model should be reported. For validation studies a quote like "model's discrimination and calibration was assessed" is considered adequate. If done, methods of updating should be reported.*				Y
	x	Results for model discrimination are reported in the abstract<br>*This should be reported separately for development and validation if a study includes both development and validation.*				Y
	xi	Results for model calibration are reported in the abstract<br>*This should be reported separately for development and validation if a study includes both development and validation.*				Y
	xii	Conclusions are reported in the abstract<br>*In publications addressing both model development and validation, there is no need for separate conclusions for both; one conclusion is sufficient.*				Y
**Background and objectives**	**3a**	**Explain the medical context (including whether diagnostic or prognostic) and rationale for developing or validating the multivariable prediction model, including references to existing models.**	**Score 1 if both elements are scored as "Y"**	**Score 1 if both elements are scored as "Y"**	**Score 1 if both elements are scored as "Y"**	**Score 1 if both elements are scored as "Y"**
	i	The background and rationale are presented				Y
	ii	Reference to existing models is included (or stated that there are no existing models)				Y
	**3b**	**Specify the objectives, including whether the study describes the development or validation of the model or both.**	**Score 1 if element is scored as "Y"**	**Score 1 if element is scored as "Y"**	**Score 1 if element is scored as "Y"**	**Score 1 if element is scored as "Y"**

	i	It is stated whether the study describes development and/or validation and/or incremental (added) value				Y
**Methods**						
**Source of data**	**4a**	**Describe the study design or source of data (e.g., randomized trial, cohort, or registry data), separately for the development and validation data sets, if applicable.**	**Score 1 if element is scored as "Y"**	**Score 1 if element is scored as "Y"**	**Score 1 if element is scored as "Y"**	**Score 1 if element is scored as "Y"**
	i	The study design/source of data is described<br>*E.g. Prospectively designed, existing cohort, existing RCT, registry/medical records, case control, case series.*<br>*This needs to be explicitly reported; reference to this information in another article alone is insufficient.*				Y
	**4b**	**Specify the key study dates, including start of accrual; end of accrual; and, if applicable, end of follow-up.**	**Score 1 if all elements are scored as "Y", "NA", or "R"**	**Score 1 if all elements are scored as "Y", "NA", or "R"**	**Score 1 if all elements are scored as "Y", "NA", or "R"**	**Score 1 if all elements are scored as "Y", "NA", or "R"**
	i	The starting date of accrual is reported				Y
	ii	The end date of accrual is reported				Y
	iii	The length of follow-up and prediction horizon/time frame are reported, if applicable<br>*E.g. "Patients were followed from baseline for 10 years" and "10-year prediction of…"; notably for prognostic studies with long term follow-up.*<br>*If this is not applicable for an article (i.e. diagnostic study or no follow-up), then score Not applicable.*				NA
**Participants**	**5a**	**Specify key elements of the study setting (e.g., primary care, secondary care, general population) including number and location of centres.**	**Score 1 if all elements are scored as "Y" or "R"**	**Score 1 if all elements are scored as "Y" or "R"**	**Score 1 if all elements are scored as "Y" or "R"**	**Score 1 if all elements are scored as "Y" or "R"**
	i	The study setting is reported (e.g. primary care, secondary care, general population)<br>*E.g.: 'surgery for endometrial cancer patients' is considered to be enough information about the study setting.*				Y
	ii	The number of centres involved is reported<br>*If the number is not reported explicitly, but can be concluded from the name of the centre/centres, or if clearly a single centre study, score Yes.*				Y
	iii	The geographical location (at least country) of centres involved is reported<br>*If no geographical location is specified, but the location can be concluded from the name of the centre(s), score Yes.*				Y
	**5b**	**Describe eligibility criteria for participants.**	**Score 1 if element is scored as "Y"**	**Score 1 if element is scored as "Y"**	**Score 1 if element is scored as "Y"**	**Score 1 if element is scored as "Y"**

	i	In-/exclusion criteria are stated<br>*These should explicitly be stated. Reasons for exclusion only described in a patient flow is not sufficient.*				Y
	**5c**	**Give details of treatments received, if relevant.**	**Score 1 if element is scored as "Y"; score *Not applicable* if element is scored as "NA"**	**Score 1 if element is scored as "Y"; score *Not applicable* if element is scored as "NA"**	**Score 1 if element is scored as "Y"; score *Not applicable* if element is scored as "NA"**	**Score 1 if element is scored as "Y"; score *Not applicable* if element is scored as "NA"**
	i	Details of any treatments received are described<br>*This item is notably for prognostic modelling studies and is about treatment at baseline or during follow-up. The 'if relevant' judgment of treatment requires clinical knowledge and interpretation.*<br>*If you are certain that treatment was not relevant, e.g. in some diagnostic model studies, score Not applicable*				Y
**Outcome**	**6a**	**Clearly define the outcome that is predicted by the prediction model, including how and when assessed.**	**Score 1 if all elements are scored as "Y" or "R"**	**Score 1 if all elements are scored as "Y" or "R"**	**Score 1 if all elements are scored as "Y" or "R"**	**Score 1 if all elements are scored as "Y" or "R"**
	i	The outcome definition is clearly presented<br>*This should be reported separately for development and validation if a publication includes both.*				Y
	ii	It is described how outcome was assessed (including all elements of any composite, for example CVD [e.g. MI, HF, stroke]).				Y
	iii	It is described when the outcome was assessed (time point(s) since T0)				NA
	**6b**	**Report any actions to blind assessment of the outcome to be predicted.**	**Score 1 if element is scored as "Y"**	**Score 1 if element is scored as "Y"**	**Score 1 if element is scored as "Y"**	**Score 1 if element is scored as "Y"**
	i	Actions to blind assessment of outcome to be predicted are reported<br>*If it is clearly a non-issue (e.g. all-cause mortality or an outcome not requiring interpretation), score Yes. In all other instances, an explicit mention is expected.*				Y
**Predictors**	**7a**	**Clearly define all predictors used in developing or validating the multivariable prediction model, including how and when they were measured.**	**Score 1 if all elements are scored as "Y" or "R"**	**Score 1 if all elements are scored as "Y" or "R"**	**Score 1 if all elements are scored as "Y" or "R"**	**Score 1 if all elements are scored as "Y" or "R"**

	i	All predictors are reported<br>*For development, "all predictors" refers to all predictors that potentially could have been included in the 'final' model (including those considered in any univariable analyses).*<br>*For validation, "all predictors" means the predictors in the model being evaluated.*				Y
	ii	Predictor definitions are clearly presented				Y
	iii	It is clearly described how the predictors were measured				Y
	iv	It is clearly described when the predictors were measured				Y
	**7b**	**Report any actions to blind assessment of predictors for the outcome and other predictors.**	**Score 1 if both elements are scored as "Y"**	**Score 1 if both elements are scored as "Y"**	**Score 1 if both elements are scored as "Y"**	**Score 1 if both elements are scored as "Y"**
	i	It is clearly described whether predictor assessments were blinded for outcome<br>*For predictors for which it is clearly a non-issue (e.g. automatic blood pressure measurement, age, sex) and for instances where the predictors were clearly assessed before outcome assessment, score Yes. For all other predictors an explicit mention is expected.*				Y
	ii	It is clearly described whether predictor assessments were blinded for the other predictors				Y
**Sample size**	**8**	**Explain how the study size was arrived at.**	**Score 1 if element is scored as "Y"**	**Score 1 if element is scored as "Y"**	**Score 1 if element is scored as "Y"**	**Score 1 if element is scored as "Y"**
	i	It is explained how the study size was arrived at<br>*Is there any mention of sample size, e.g. whether this was done on statistical grounds or practical/logistical grounds (e.g. an existing study cohort or data set of a RCT was used)?*				Y
**Missing data**	**9**	**Describe how missing data were handled (e.g., complete-case analysis, single imputation, multiple imputation) with details of any imputation method.**	**Score 1 if all elements are scored as "Y" or "NA"**	**Score 1 if all elements are scored as "Y" or "NA"**	**Score 1 if all elements are scored as "Y" or "NA"**	**Score 1 if all elements are scored as "Y" or "NA"**

	i	The method for handling missing data (predictors and outcome) is mentioned<br>*E.g. Complete case (explicit mention that individuals with missing values have been excluded), single imputation, multiple imputation, mean/median imputation.*<br>*If there is no missing data, there should be an explicit mention that there is no missing data for all predictors and outcome. If so, score Yes.*<br>*If it is unclear whether there is missing data (from e.g. the reported methods or results), score No.*<br>*If it is clear there is missing data, but the method for handling missing data is unclear, score No.*				Y
	ii	If missing data were imputed, details of the software used are given<br>*When under 9i explicit mentioning of no missing data, complete case analysis or no imputation applied, score Not applicable*				Y
	iii	If missing data were imputed, a description of which variables were included in the imputation procedure is given.<br>*When under 9i explicit mentioning of no missing data, complete case analysis or no imputation applied, score Not applicable*				Y
	iv	If multiple imputation was used, the number of imputations is reported<br>*When under 9i explicit mentioning of no missing data, complete case analysis or no imputation applied, score Not applicable*				Y
**Statistical analysis methods**	**10a**	**Describe how predictors were handled in the analyses.**	**Score 1 if all elements are scored as "Y" or "NA"**	**Not applicable**	**Score 1 if all elements are scored as "Y" or "NA"**	**Score 1 if all elements are scored as "Y" or "NA"**
	i	For continuous predictors it is described whether they were modelled as linear, nonlinear (type of transformation specified) or categorized<br>*A general statement is sufficient, no need to describe this for each predictor separately.*<br>*If no continuous predictors were reported, score Not applicable.*				Y
	ii	For categorical or categorized predictors, the cut-points were reported<br>*If no categorical or categorized predictors were reported, score Not applicable.*				NA
	iii	For categorized predictors the method to choose the cut-points was clearly described<br>*If no categorized predictors, score Not applicable.*				NA
	**10b**	**Specify type of model, all model-building procedures (including any predictor selection), and method for internal validation.**	**Score 1 if all elements are scored as "Y" or "NA"**	**Not applicable**	**Score 1 if all elements are scored as "Y" or "NA"**	**Score 1 if all elements are scored as "Y" or "NA"**
	i	The type of statistical model is reported<br>*E.g. Logistic, Cox, other regression model (e.g. Weibull, ordinal), other statistical modelling (e.g. neural network)*				Y

	ii	The approach used for predictor selection <u>before</u> modelling is described<br>*'Before modelling' means before any univariable or multivariable analysis of predictor-outcome associations.*<br>*If no predictor selection before modelling is done, score Not applicable.*<br>*If it is unclear whether predictor selection before modelling is done, score No.*<br>*If it is clear there was predictor selection before modelling but the method was not described, score No.*				Y
	iii	The approach used for predictor selection <u>during</u> modelling is described<br>*E.g. Univariable analysis, stepwise selection, bootstrap, Lasso.*<br>*'During modelling' includes both univariable or multivariable analysis of predictor-outcome associations.*<br>*If no predictor selection during modelling is done (so-called full model approach), score Not applicable.*<br>*If it is unclear whether predictor selection during modelling is done, score No.*<br>*If it is clear there was predictor selection during modelling but the method was not described, score No.*				Y
	iv	Testing of interaction terms is described<br>*If it is explicitly mentioned that interaction terms were not addressed in the prediction model, score Yes.*<br>*If interaction terms were included in the prediction model, but the testing is not described, score No.*				NA
	v	Testing of the proportionality of hazards in survival models is described<br>*If no proportional hazard model is used, score Not applicable.*				NA
	vi	Internal validation is reported<br>*E.g. Bootstrapping, cross validation, split sample.*<br>*If the use of internal validation is clearly a non-issue (e.g. in case of very large data sets), score Yes. For all other situations an explicit mention is expected.*				Y
	**10c**	**For validation, describe how the predictions were calculated.**	**Not applicable**	**Score 1 if extraction item is scored as "Y"**	**Score 1 if extraction item is scored as "Y"**	**Score 1 if extraction item is scored as "Y"**
	i.	It is described how predictions for individuals (in the validation set) were obtained from the model being validated<br>*E.g. Using the original reported model coefficients with or without the intercept, and/or using updated or refitted model coefficients, or using a nomogram, spreadsheet or web calculator.*				Y

	**10d**	**Specify all measures used to assess model performance and, if relevant, to compare multiple models.**[2]<br>*These should be described in the methods section of the paper (item 16 addresses the reporting of the results for model performance).*	**Score 1 if elements 10di and 10dii are scored as "Y"**[2]	**Score 1 if elements 10di and 10dii are scored as "Y"**[2]	**Score 1 if all elements are scored as "Y"**[2]	**Score 1 if elements 10di and 10dii are scored as "Y"**[2]
	i	Measures for model discrimination are described<br>*E.g. C-index / area under the ROC curve*				Y
	ii	Measures for model calibration are described<br>*E.g. calibration plot, calibration slope or intercept, calibration table, Hosmer Lemeshow test, O/E ratio.*				Y
	iii	Other performance measures are described<br>*E.g. R^2, Brier score, predictive values, sensitivity, specificity, AUC difference, decision curve analysis, net reclassification improvement, integrated discrimination improvement, AIC*				Y
	**10e**	**Describe any model updating (e.g., recalibration) arising from the validation, if done.**	**Not applicable**	**Score 1 if element is scored as "Y"; score *Not applicable* if element is scored as "NA"**	**Score 1 if element is scored as "Y"; score *Not applicable* if element is scored as "NA"**	**Score 1 if element is scored as "Y"; score *Not applicable* if element is scored as "NA"**
	i	A description of model-updating is given<br>*E.g. Intercept recalibration, regression coefficient recalibration, refitting the whole model, adding a new predictor*<br>*If updating was done, it should be clear which updating method was applied to score Yes.*<br>*If it is not explicitly mentioned that updating was applied in the study, score this item as 'Not applicable'.*				NA
**Risk groups**	**11**	**Provide details on how risk groups were created, if done.**	**Score 1 if element is scored as "Y"; score *Not applicable* if**	**Score 1 if element is scored as "Y"; score *Not applicable* if**	**Score 1 if element is scored as "Y"; score *Not applicable* if**	**Score 1 if element is scored as "Y"; score *Not applicable* if element is scored as "NA"**

[2] Discrimination and calibration are the two key aspects that characterize the performance of a prediction model and the TRIPOD guideline states that these two measures should be mentioned in every prediction model report. Various other measures of model performance can sometimes be reported (see examples provided at data extraction element 10diii). For reports on D and V and DV, we considered that discrimination and calibration had to be reported to adhere to item 10d. Other overall performance measures such as (R^2, Brier score or AIC) were not deemed essential for the scoring of overall adherence in D, V and D+V reports. For reports on the incremental value (IV reports) the reporting of other performance measures, like AUC difference or net reclassification improvement, were considered essential in addition to discrimination and calibration.

			element is scored as "NA"	element is scored as "NA"	element is scored as "NA"	
	i	If risk groups were created, risk group boundaries (risk thresholds) are specified<br>*Score this item separately for development and validation if a study includes both development and validation.*<br>*If risk groups were not created, score this item as not applicable.*				NA
**Development vs. validation**	**12**	**For validation, identify any differences from the development data in setting, eligibility criteria, outcome and predictors.**	**Not applicable**	**Score 1 if element is scored as "Y"**	**Score 1 if element is scored as "Y" or "NA"**	**Score 1 if element is scored as "Y"**
	i	Differences or similarities in <u>definitions</u> with the development study are described<br>*Mentioning of any differences in all four (setting, eligibility criteria, predictors and outcome) is required to score Yes.*<br>*If it is explicitly mentioned that there were no differences in setting, eligibility criteria, predictors and outcomes, score Yes.*<br>*For incremental value reports, in case additional predictors are not added to a previously developed prediction model but rather added to conventional predictors in a newly fitted model, score Not applicable.*				Y
**Results**						
**Participants**	**13a**	**Describe the flow of participants through the study, including the number of participants with and without the outcome and, if applicable, a summary of the follow-up time. A diagram may be helpful.**	**Score 1 if all elements are scored as "Y" or "NA"**	**Score 1 if the elements are scored as "Y" or "NA"**	**Score 1 if all elements are scored as "Y" or "NA"**	**Score 1 if all elements are scored as "Y" or "NA"**
	i	The flow of participants is reported				Y
	ii	The number of participants with and without the outcome are reported<br>*If outcomes are continuous, score Not applicable.*				Y
	iii	A summary of follow-up time is presented<br>*This notably applies to prognosis studies and diagnostic studies with follow-up as diagnostic outcome.*<br>*If this is not applicable for an article (i.e. diagnostic study or no follow-up), then score Not applicable.*				NA
	**13b**	**Describe the characteristics of the participants (basic demographics, clinical features, available predictors), including the number of participants with missing data for predictors and outcome.**	**Score 1 if all elements are scored as "Y"**	**Score 1 if all elements are scored as "Y"**	**Score 1 if all elements are scored as "Y"**	**Score 1 if all elements are scored as "Y"**
	i	Basic demographics are reported				Y
	ii	Summary information is provided for all predictors included in the final developed/validated model				Y

	iii	The number of participants with missing data for predictors is reported				Y
	iv	The number of participants with missing data for the outcome is reported				Y
	**13c**	**For validation, show a comparison with the development data of the distribution of important variables (demographics, predictors and outcome).**	**Not applicable**	**Score 1 if all elements are scored as "Y"**	**Score 1 if all elements are scored as "Y" or "NA"**	**Score 1 if all elements are scored as "Y"**
	i	Demographic characteristics (at least age and gender) of the validation study participants are reported along with those of the original development study *For incremental value reports, in case additional predictors are not added to a previously developed prediction model but rather added to conventional predictors in a newly fitted model, score Not applicable.*				Y
	ii	Distributions of predictors in the model of the validation study participants are reported along with those of the original development study *For incremental value reports, in case additional predictors are not added to a previously developed prediction model but rather added to conventional predictors in a newly fitted model, score Not applicable.*				Y
	iii	Outcomes of the validation study participants are reported along with those of the original development study *For incremental value reports, in case additional predictors are not added to a previously developed prediction model but rather added to conventional predictors in a newly fitted model, score Not applicable.*				Y
**Model development**	**14a**	**Specify the number of participants and outcome events in each analysis.**	**Score 1 if both elements are scored as "Y" or "NA"**	**Not applicable**	**Score 1 if both elements are scored as "Y" or "NA"**	**Score 1 if both elements are scored as "Y" or "NA"**
	i	The number of participants in each analysis (e.g. in the analysis of each model if more than one model is developed) is specified				Y
	ii	The number of outcome events in each analysis is specified (e.g. in the analysis of each model if more than one model is developed) *If outcomes are continuous, score Not applicable.*				NA
	**14b**	**If done, report the unadjusted association between each candidate predictor and outcome.**	**Score 1 if element is scored as "Y"; score *Not applicable* if element is scored as "NA"**	**Not applicable**	**Score 1 if element is scored as "Y"; score *Not applicable* if element is scored as "NA"**	**Score 1 if element is scored as "Y"; score *Not applicable* if element is scored as "NA"**
	i	The unadjusted associations between each predictor and outcome are reported *If any univariable analysis is mentioned in the methods but not in the results, score No.* *If nothing on univariable analysis (in methods or results) is reported, score this item as Not applicable*				Y

**Model specification**	**15a**	**Present the full prediction model to allow predictions for individuals (i.e., all regression coefficients, and model intercept or baseline survival at a given time point).**	**Score 1 if both elements are scored as "Y"**	**Not applicable**	**Score 1 if both elements are scored as "Y"**	**Score 1 if both elements are scored as "Y"**
	i	The regression coefficient (or a derivative such as hazard ratio, odds ratio, risk ratio) for each predictor in the model is reported				Y
	ii	The intercept or the cumulative baseline hazard (or baseline survival) for at least one time point is reported				NA
	**15b**	**Explain how to use the prediction model.**	**Score 1 if element is scored as "Y"**	**Not applicable**	**Score 1 if element is scored as "Y"**	**Score 1 if element is scored as "Y"**
	i	An explanation (e.g. a simplified scoring rule, chart, nomogram of the model, reference to online calculator, or worked example) is provided to explain how to use the model for individualised predictions.				Y
**Model performance**	**16**	**Report performance measures (with confidence intervals) for the prediction model.[3]** *These should be described in results section of the paper (item 10 addresses the reporting of the methods for model performance).*	**Score 1 if elements 16i-16iii are scored as "Y"[3]**	**Score 1 if elements 16i-16iii are scored as "Y"[3]**	**Score 1 if all elements are scored as "Y"[3]**	**Score 1 if elements 16i-16iii are scored as "Y"[3]**
	i	A discrimination measure is presented *E.g. C-index / area under the ROC curve*				Y
	ii	The confidence interval (or standard error) of the discrimination measure is presented				N
	iii	Measures for model calibration are described *E.g. calibration plot, calibration slope or intercept, calibration table, Hosmer Lemeshow test, O/E ratio.*				Y
	iv	Other model performance measures are presented *E.g. R^2, Brier score, predictive values, sensitivity, specificity, AUC difference, decision curve analysis, net reclassification improvement, integrated discrimination improvement, AIC.*				Y
**Model updating**	**17**	**If done, report the results from any model updating (i.e., model specification, model performance, recalibration).** *If updating was not done, score this TRIPOD item as 'Not applicable'.*	**Not applicable**	**Score 1 if all elements are scored as "Y"**	**Not applicable**	**Score 1 if all elements are scored as "Y"**

[3] See also footnote 2. Discrimination and calibration are the two key aspects that characterize the performance of a prediction model and the TRIPOD guideline states that these two measures should be reported in every prediction model report. Various other measures of model performance can sometimes be reported (see examples provided at data extraction element 16iv). For reports on D and V and D+V, we considered that discrimination and calibration had to be reported to adhere to item 16. Other overall performance measures such as (R^2, Brier score or AIC) were not deemed essential for the scoring of overall adherence in D, V and D+V reports. For reports on the incremental value (IV reports) the reporting of other performance measures, like AUC difference or net reclassification improvement, were considered essential in addition to discrimination and calibration.

	i	The updated regression coefficients for each predictor in the model are reported<br>*If model updating was described as 'not needed', score Yes.*				NA
	ii	The updated intercept or cumulative baseline hazard or baseline survival (for at least one time point) is reported<br>*If model updating was described as 'not needed', score Yes.*				NA
	iii	The discrimination of the updated model is reported				NA
	iv	The confidence interval (or standard error) of the discrimination measure of the updated model is reported				NA
	v	The calibration of the updated model is reported				NA
**Discussion**						
**Limitations**	**18**	**Discuss any limitations of the study (such as nonrepresentative sample, few events per predictor, missing data).**	**Score 1 if element is scored as "Y"**	**Score 1 if element is scored as "Y"**	**Score 1 if element is scored as "Y"**	**Score 1 if element is scored as "Y"**
	i	Limitations of the study are discussed<br>*Stating any limitation is sufficient.*				Y
**Interpretation**	**19a**	**For validation, discuss the results with reference to performance in the development data, and any other validation data.**	**Not applicable**	**Score 1 if element is scored as "Y"**	**Score 1 if element is scored as "Y"**	**Score 1 if element is scored as "Y"**
	i	Comparison of results to reported performance in development studies and/or other validation studies is given				Y
	**19b**	**Give an overall interpretation of the results considering objectives, limitations, results from similar studies and other relevant evidence.**	**Score 1 if element is scored as "Y"**	**Score 1 if element is scored as "Y"**	**Score 1 if element is scored as "Y"**	**Score 1 if element is scored as "Y"**
	i	An overall interpretation of the results is given				Y
**Implications**	**20**	**Discuss the potential clinical use of the model and implications for future research.**	**Score 1 if both elements are scored as "Y"**	**Score 1 if both elements are scored as "Y"**	**Score 1 if both elements are scored as "Y"**	**Score 1 if both elements are scored as "Y"**
	i	The potential clinical use is discussed<br>*E.g. an explicit description of the context in which the prediction model is to be used (e.g. to identify high risk groups to help direct treatment, or to triage patients for referral to subsequent care).*				Y
	ii	Implications for future research are discussed<br>*E.g. a description of what the next stage of investigation of the prediction model should be, such as "We suggest further external validation".*				Y
**Other information**						
**Supplementary information**	**21**	**Provide information about the availability of supplementary resources, such as study protocol, web calculator, and data sets.**	**Not included in overall scoring**	**Not included in overall scoring**	**Not included in overall scoring**	**Not included in overall scoring**

	i	Information about supplementary resources is provided				Y
**Funding**	**22**	**Give the source of funding and the role of the funders for the present study.**	**Score 1 if both elements are scored as "Y"**	**Score 1 if both elements are scored as "Y"**	**Score 1 if both elements are scored as "Y"**	**Score 1 if both elements are scored as "Y"**
	i	The source of funding is reported or there is explicit mention that there was no external funding involved				Y
	ii	The role of funders is reported or there is explicit mention that there was no external funding				Y
