# Supplementary material for: Towards a proteomic plasma biomarker panel for diagnosing vasculitis remission
Source: Nat Commun. 2026 Jul 21;17:6825. doi: 10.1038/s41467-026-75755-6 (PMC13389191; doi:10.1038/s41467-026-75755-6)
Supplement: Supplementary file 2 — Description of Additional Supplementary Files [file 41467_2026_75755_MOESM2_ESM.pdf]

Description of Additional Supplementary Files

Supplementary Data 1: Global proteome data matrix (DIA-MS), discovery cohort.

Supplementary Data 2: GO enrichment analysis results, global proteome data, discovery cohort.

Supplementary Data 3: Metadeconfounder results, global proteome data, discovery cohort.

Supplementary Data 4: Peptide selection for Parallel Reaction Monitoring-Mass (PRM-MS).

Supplementary Data 5: (PRM-MS)-based targeted proteomic data matrix, 21-panel, discovery cohort.

Supplementary Data 6: TQL-PRM data matrix with 7-protein panel from discovery and validation cohort, including concentrations values.
