# Supplementary material for: Towards a proteomic plasma biomarker panel for diagnosing vasculitis remission
Source: Nat Commun. 2026 Jul 21;17:6825. doi: 10.1038/s41467-026-75755-6 (PMC13389191; doi:10.1038/s41467-026-75755-6)
Supplement: Supplementary file 9 — Reporting Summary [file 41467_2026_75755_MOESM9_ESM.pdf]

Corresponding author(s): Ralph Kettritz
Last updated by author(s): Jun 3, 2026

Reporting Summary

Nature Portfolio wishes to improve the reproducibility of the work that we publish. This form provides structure for consistency and transparency in reporting. For further information on Nature Portfolio policies, see our Editorial Policies and the Editorial Policy Checklist.

Statistics

For all statistical analyses, confirm that the following items are present in the figure legend, table legend, main text, or Methods section.

n/a	Confirmed	
☐	☒	The exact sample size (*n*) for each experimental group/condition, given as a discrete number and unit of measurement
☐	☒	A statement on whether measurements were taken from distinct samples or whether the same sample was measured repeatedly
☐	☒	The statistical test(s) used AND whether they are one- or two-sided<br>*Only common tests should be described solely by name; describe more complex techniques in the Methods section.*
☐	☒	A description of all covariates tested
☐	☒	A description of any assumptions or corrections, such as tests of normality and adjustment for multiple comparisons
☐	☒	A full description of the statistical parameters including central tendency (e.g. means) or other basic estimates (e.g. regression coefficient) AND variation (e.g. standard deviation) or associated estimates of uncertainty (e.g. confidence intervals)
☐	☒	For null hypothesis testing, the test statistic (e.g. *F*, *t*, *r*) with confidence intervals, effect sizes, degrees of freedom and *P* value noted<br>*Give P values as exact values whenever suitable.*
☒	☐	For Bayesian analysis, information on the choice of priors and Markov chain Monte Carlo settings
☒	☐	For hierarchical and complex designs, identification of the appropriate level for tests and full reporting of outcomes
☐	☒	Estimates of effect sizes (e.g. Cohen's *d*, Pearson's *r*), indicating how they were calculated

Our web collection on statistics for biologists contains articles on many of the points above.

Software and code

Policy information about availability of computer code

Data collection	Mass spectrometry data were aquired using Xcalibur software v4.6.67.17 (Thermo Scientific).
Data analysis	Raw proteomics data from DIA runs were analyzed with Spectronaut software (version 14.3) with a human Uniprot database (2021-01) and a universal protein contaminants list, using and project specific library. PRM data analysis was carried out using the Skyline software package (24.1.0.199). Gene Ontology enrichment was performed using g:Profiler version e113_eg59_p19_6be5291); Gene Ontology terms redundancy was were reduced using the REVIGO (Reduce and Visualize Gene Ontology, version 1.8.2) web tool and hierarchical depth filter using AmiGO2 (version 2.5.17); for Confounder assessment metadeconfoundR package (v0.3.0) was used. All analyses were conducted in R version 4.1.2, employing the tidyverse (v1.3.1), caret (v6.0-94), pROC (v1.18.5), glmnet (v4.1-7), enrichGO (v3.0.4), and rmda (v1.6) packages. Data visualization was performed with ggplot2 (v3.5.1) and GraphPad® Prism V.8.4.1 software.

For manuscripts utilizing custom algorithms or software that are central to the research but not yet described in published literature, software must be made available to editors and reviewers. We strongly encourage code deposition in a community repository (e.g. GitHub). See the Nature Portfolio guidelines for submitting code & software for further information.

Data

Policy information about availability of data

All manuscripts must include a data availability statement. This statement should provide the following information, where applicable:

- Accession codes, unique identifiers, or web links for publicly available datasets
- A description of any restrictions on data availability
- For clinical datasets or third party data, please ensure that the statement adheres to our policy

The mass spectrometry proteomics raw data have been deposited to the ProteomeXchange Consortium via the PRIDE partner repository with the dataset identifier PXD079232". Code and full reproducibility documentation including all random seeds are provided at GitHub: https://github.com/Theda-sys/vasculitis-remission-proteomics (https://doi.org/10.5281/zenodo.20450403).

Research involving human participants, their data, or biological material

Policy information about studies with human participants or human data. See also policy information about sex, gender (identity/presentation), and sexual orientation and race, ethnicity and racism.

Reporting on sex and gender	Clinical metadata including sex are summarised in Supplementary Table 1 and 11. Both male and female samples were included without showing any imbalance.
Reporting on race, ethnicity, or other socially relevant groupings	No data collection concerning race, ethncity or other socially relevant groupings was performed.
Population characteristics	Patients and healthy human individuals: Patients with AAV, based on Chapel Hill Consensus Conference criteria, were grouped according to ANCA specificity (PR3-ANCA versus MPO-ANCA) and disease activity (active versus remission). Disease activity was assessed by the Birmingham Vasculitis Activity Score Version 3 (BVAS). Individual samples have been pseudonymized to comply with general data protection regulation. Metadata, including clinical parameter, are described in Supplementary Table 1 and 11.<br>test of independence for categorical variables (sex), a summary of which can be found in the corresponding Extended Data Tables.
Recruitment	Patients with AAV were recruited (during Jan 2020 and July 2021) in the Department of Nephrology and Medical Intensive Care, Charité Universitätsmedizin Exploratory Cohort: Berlin, Department of Nephrology, Helios Klinikum Berlin-Buch, Berlin, and Department of Nephrology, Endocrinology and Diabetology, Ernst von Bergmann Klinikum, Potsdam, all Germany. Validation Cohort: Patients were recruted during Jan 2019 and Oct 2023 in the General University Hospital Prague, Czech Republic
Ethics oversight	The study was approved by the local ethic committee of Charité-Universitätsmedizin Berlin, Germany (EA4/025/18) and the General University Hospital Prague, Czech Republic (No. 1443/11 S-IV). Patients and healthy controls (HC) gave written, informed consent.

Note that full information on the approval of the study protocol must also be provided in the manuscript.

Field-specific reporting

Please select the one below that is the best fit for your research. If you are not sure, read the appropriate sections before making your selection.

[x] Life sciences [ ] Behavioural & social sciences [ ] Ecological, evolutionary & environmental sciences

For a reference copy of the document with all sections, see nature.com/documents/nr-reporting-summary-flat.pdf

Life sciences study design

All studies must disclose on these points even when the disclosure is negative.

Sample size	Maximal available AAV-patient numbers in each group were recruited during study time (Jan 2020 and July 2021) for the exploratory cohort and during Jan 2019 and Oct 2023 for the validation cohort
Data exclusions	For global proteome analyses two of 114 samples were excluded from analysis because of low data quality. Similarly, two samples were excluded from 21-panel PRM analyses. Patients were excluded from all PRM analyses due to recent plasma exchange (n = 4), septic constellation (n = 2), and high dose steroid treatment (n = 4) that exceeded established protocols at sampling.
Replication	One replicate was used and analysed per patient. For global proteome and PRM measurements peptides from independent sample processing batches were used. TQL measurements correlated excellently with the corresponding data from global proteome measurements (Extended Data Figure 2).
Randomization	Samples were processed in randomized order. For mass spectrometry measurements, an additional level of stratified randomization was applied, ensuring equal distribution of clinical groups over the entire measurement, with different run order for untargeted global measurements and targeted PRM measurements.

Blinding	Clinical patient characterization and proteome measurements and data analyses were performed independently. Sample conditions were blinded to investigators during sample processing and measurements.

Reporting for specific materials, systems and methods

We require information from authors about some types of materials, experimental systems and methods used in many studies. Here, indicate whether each material, system or method listed is relevant to your study. If you are not sure if a list item applies to your research, read the appropriate section before selecting a response.

Materials & experimental systems

n/a	Involved in the study
☒	☐ Antibodies
☒	☐ Eukaryotic cell lines
☒	☐ Palaeontology and archaeology
☒	☐ Animals and other organisms
☐	☒ Clinical data
☒	☐ Dual use research of concern
☒	☐ Plants

Methods

n/a	Involved in the study
☒	☐ ChIP-seq
☒	☐ Flow cytometry
☒	☐ MRI-based neuroimaging

Clinical data

Policy information about clinical studies

All manuscripts should comply with the ICMJE guidelines for publication of clinical research and a completed CONSORT checklist must be included with all submissions.

Clinical trial registration	No clinical trial was performed.
Study protocol	Prospective collection of blood and clinical data with written informed consent.
Data collection	Clinical and routine laboratory data from patients were collected (see Supplementary Table 1 and 6), and samples were assessed based on material availability.
Outcomes	n/a

Plants

Seed stocks	---
Novel plant genotypes	---
Authentication	---
