# Supplementary material for: Towards a proteomic plasma biomarker panel for diagnosing vasculitis remission
Source: Nat Commun. 2026 Jul 21;17:6825. doi: 10.1038/s41467-026-75755-6 (PMC13389191; doi:10.1038/s41467-026-75755-6)
Supplement: Supplementary file 10 — Peer Review file [file 41467_2026_75755_MOESM10_ESM.pdf]

nature portfolio

Peer Review File

Towards a proteomic plasma biomarker panel for diagnosing vasculitis remission

Corresponding Author: Professor Ralph Kettritz

This file contains all reviewer reports in order by version, followed by all author rebuttals in order by version.

Attachments originally included by the reviewers as part of their assessment can be found at the end of this file.

Version 0:

Reviewer comments:

Reviewer #1

(Remarks to the Author)
See attachment

(Remarks on code availability)

Reviewer #2

(Remarks to the Author)
This is an impressive study. Although I'm not an expert in proteomics nor in some of the data-analysis techniques, in the end it really doesn't matter how the authors came up with their high-performing 7-protein assay, because it also performed very well in an independent cohort. The AUC is far higher than the frequent benchmark of 0.8 as a minimum for having value, and the positive likelihood ratio, if they had calculated it, would be well over 10, another common benchmark for clinical utility. The authors are correct that this study addresses a clinically important need in which research advances over many years have not yet led to new clinically useful tests.

I only have two sets of comments:

1. Confounder-aware feature selection. The authors excluded proteins that were significantly associated with CRP, GFR, or "other patient metadata." I think these should be specified, sorry if I missed that in the supplementary tables (it isn't in the methods). Based on supplementary tables 1 and 6, lack of association with either hematocrit/hemoglobin or platelets would be particularly important. In the unlikely event that that wasn't done, that should be added as a limitation. It would be preferable to show correlation plots with GFR rather than just CRP (extended data fig 2), and also Hct/Hgb and platelets if done. In the process of looking for this, I noticed that the call-outs to Extended Data Figure 1 don't match that figure, including a call-out to 1e, when the figure only has a-d. I also didn't see a call-out to Extended Data Figure 2, which I expect was intended to be referenced in that section on page 8.

2. Effects of treatment. I don't understand Figure 4d, which is essential for saying that possible direct effects of treatment were explored and that the finding was similar regardless. The legend, and the acocmpanying text, indicate that only samples from patients with active disease were used. Is so, what is the dichotomous outcome being modeled with an AUC curve? If it isn't active disease vs remission, then that needs to be clear in both the text and the legend, and I'm skeptical that any other comparison would show that treatment has negligible direct effects on assay results. Even if the problem is simply an error in not reporting that samples from patients in remission were also used in this analysis, I still think it would be important to add as a limitation in the discussion something like, "The study was too small, and the diseases and details of treatment are too heterogeneous, to definitively rule out effects of medications on biomarker concentrations independent of disease activity."

(Remarks on code availability)

Reviewer #3

(Remarks to the Author)
Overall, well planned study with powerful tools. I agree with your thought process and is thoroughly executed. I have minor comments to point out.

(1) It seems that, throughout the paper, the purpose of the search for biomarkers is to “diagnose” AND “evaluate the activity”. In fact, MPO and PR3 levels can be used for both diagnosis and evaluation of activity. However, the title of this paper is “towards a proteomic plasma biomarker panel for diagnosing vasculitis remission”. In the paper, few more sentences need to be added to clarify that not only seven-biomakers can help to evaluate the AAV activities but also aid in diagnosis.

(2) (line 90-92): In practice, there are AAV patients whose laboratory results do not match with actual disease activities. However, I believe that majority of them actually show match between the lab results and disease activities. Please provide approximate percentage of patients whose disease activities are not represented by currents biomarkers (ANCA and CRP)

(3) In few supplementary tables, EGPA is listed under MPO-AAV. In fact, large portion of EGPA patients have MPO titers and may play pathogenic roles. However, MPA and EGPA are clearly different entities, and it needs to be differentiated. Please clarify in the writing.

(Remarks on code availability)

Reviewer #4

(Remarks to the Author)
I co-reviewed this manuscript with one of the reviewers who provided the listed reports. This is part of the Nature Communications initiative to facilitate training in peer review and to provide appropriate recognition for Early Career Researchers who co-review manuscripts.

(Remarks on code availability)
It appears that the dataset/record at Zenodo with DOI 10.5281/zenodo.16927900 indicates “Files: Restricted” — the files are not publicly accessible without permission. Could you please grant reviewer access or send the code repository link?

Reviewer #5

(Remarks to the Author)
The need for better biomarkers in AAV in clear, as the authors state, treatment is toxic and there is often uncertainty in the mind of the clinician as to when remission has occurred, the implication being that treatment can be reduced. Using plasma proteins, a panel of 7 has been identified which discriminate between active and remission states and perform better than existing biomarkers. This panel could readily be translated to a clinically useful tool.

Major points:

1. A thorough and robust approach to identification and validation of protein biomarkers in AAV active and remission patients and healthy controls. Resulting in a 7 panel protein set. Important strengths are
a) Confounder-aware selection: explicit deconfounding vs CRP/eGFR before modeling.
b) External validation: independent cohort + robustness across treatment strata (naïve, GC, GC+IS).
c) Interpretability & utility: parsimonious GLM, benchmarking vs CRP/ANCA, and decision-curve analysis.
The presented description of the methodology and Figures are of a high standard, accessible to the reader and compliment the report.

2. There is potential weakness over the 'remission' definition. Because AAV is typically a relapsing disease, in many patients 'remission' is disease suppression on therapy, then relapse occurs on reduction of therapy. Guidelines have defined 'on treatment' and 'off treatment' remission for example.

3. Although there may appear to be control of systemic inflammation - CRP, ANCA, clinical assessment, patient symptoms - there can be ongoing lower level 'activity' in organs, for example in the kidney. This is often referred to as 'grumbling disease' although it can be hard to define or detect without tissue biopsy. Further work is required to understand to what extent this panel can 'detect' such lower level disease activity, yet these are the manifestations that will drive relapse and may drive chronic tissue injury.
I see the issues in comments 2 and 3 as being the main concerns I have with this paper and would like to see them further addressed under limitations or discussion of future studies.

4. Treatment is always a major confounder, especially glucocorticoids. It is notable that rituximab was widely used for

induction which helps control heterogeneity but of the 'active' patients some were on therapy and some not, and my guess is that glucocorticoid doses would have been variable. Further, for the 'remission' cohort some were on glucocorticoids and some not. Is there a way of controlling for glucocorticoid confounding ?

5. CRP and GFR are pre-defined confounders. What about ANCA ? I accept that the AAV patient groups were clearly divided by ANCA serotype, but ANCA are one element of pathogenesis and ANCA binding levels vary between patients both in their initial levels and in their response to treatment. Also, persisting ANCA has been associated with higher relapse risk.

6. As yet, there have not been studies in disease controls, i.e. other systemic inflammatory diseases, or in AAV patients with co-morbidities causing inflammation, such as infection or certain drug toxicities.

7. I would also like to see validation against organ specific features - extending the discussion over grumbling versus systemic disease. The obvious organ to do this would be the kidney and to compare the plasma protein signal with urine and possibly histology. This lies outside the aims for this report but could be mentioned as a question to be addressed in the future.

Minor points:

1. Add uncertainty + calibration (one small table/figure). Please provide 95% CIs for AUC, sensitivity, specificity, PPV/NPV and report calibration slope/intercept (or Brier score) with a small calibration plot. Rationale: Completes TRIPOD items and strengthens clinical credibility without re-analysis.

2. Stratify performance by kidney functon(one supplementary table). Add AUC (95% CI) stratified by eGFR (e.g., ≤45 vs >45 mL/min/1.73 m²). Rationale: They already deconfounded against eGFR; this simple stratification confirms robustness across renal impairment (they've already shown treatment-stratified performance).

3. One-sentence workflow clarification. State explicitly that all feature selection/tuning were performed within training data only, and that the held-out test set + external cohort were untouched until final evaluation; confirm the operating threshold was fixed from training. Rationale: Avoids any concern about data leakage; aligns with what they already did.

4. Clinicians do not usually 'rely' on BVAS, BVAS is a tool to record items of vasculitis activity and, as stated in the report, it is subjective, but really summarises the clinician's opinion on the extent of disease activity.

5. EGPA patients were included in the AAV cohorts. I accept they were MPO-ANCA positive, but they do introduce other pathogenetic pathways and further contribute to heterogeneity.

6. There is no mention of urine biomarkers in the introduction, yet the kidney is the most commonly affected vital organ and urine CD163 has been validated against kidney histology.

(Remarks on code availability)

Version 1:

Reviewer comments:

Reviewer #1

(Remarks to the Author)
The authors are to be congratulated for having very thoroughly and systematically addressed all the points I previously raised. The manuscript is greatly improved as a result and I would recommend for publication.

(Remarks on code availability)

Reviewer #2

(Remarks to the Author)
I appreciate the response and revision and do not think further revision is needed.

(Remarks on code availability)

Reviewer #3

(Remarks to the Author)
It is believed that the authors have provided sincere and sufficient responses to the comments suggested by this reviewer. This reviewer has no more comments.

(Remarks on code availability)

Reviewer #4

(Remarks to the Author)
I co-reviewed this manuscript with one of the reviewers who provided the listed reports. This is part of the Nature Communications initiative to facilitate training in peer review and to provide appropriate recognition for Early Career Researchers who co-review manuscripts.

(Remarks on code availability)
The authors provided extensive and generally well-documented R code, together with a structured README and reproducibility documentation. The code appears to be a useful and relatively reproducible resource for the community.

Reviewer #5

(Remarks to the Author)
There have been good responses to the reviewers' comments.

(Remarks on code availability)

Open Access This Peer Review File is licensed under a Creative Commons Attribution 4.0 International License, which permits use, sharing, adaptation, distribution and reproduction in any medium or format, as long as you give appropriate credit to the original author(s) and the source, provide a link to the Creative Commons license, and indicate if changes were made.
In cases where reviewers are anonymous, credit should be given to 'Anonymous Referee' and the source.
The images or other third party material in this Peer Review File are included in the article’s Creative Commons license, unless indicated otherwise in a credit line to the material. If material is not included in the article’s Creative Commons license and your intended use is not permitted by statutory regulation or exceeds the permitted use, you will need to obtain permission directly from the copyright holder.
To view a copy of this license, visit https://creativecommons.org/licenses/by/4.0/

Overview

The authors use a mass spec-based approach to perform plasma proteomics in ANCA-associated vasculitis (AAV), combining an initial untargeted approach with targeted MS. The field of plasma/serum proteomics is rapidly advancing, and this is a timely manuscript using an exciting technological approach applied to a serious autoimmune disease. While AAV is an uncommon disease, it is an important condition in terms of morbidity and mortality. Modern treatment has reduced complications from disease but comes at a cost of treatment-related morbidity, particularly infections associated with immunosuppression. The project here attempts to address an important unmet translational need by identifying biomarkers to judge disease activity which could in theory help guide treatment decisions.

The manuscript was generally clearly written, although there were issues around hyperbolic language and overstated claims about prospects for clinical translation.

I have some major concerns (including some of the statistics) but I believe these are addressable with a revised manuscript and new analyses.

<u>Major</u>

<u>Conceptual</u>
1) The authors have chosen to focus on biomarker discovery/clinical applicability rather than for biological insights into AAV. I felt that the paper would be stronger if the authors used the data to its full extent. In other words, part 1 should be what biological insights can be gained from analysis of the data before presenting biomarker analysis in part 2. Currently the biological insights are cursory/minimal. Perhaps the authors have looked at this and concluded that there is not much novel biology and thus elected not to focus on this aspect, although I must say that I would be surprised if this was the case, particularly given that they see biomarker signatures.

For the remainder of the review, I have judged the paper on its potential in terms of biomarker discovery since this is what the authors have elected to focus on.

2) The authors rightly point out the difficulty in decisions about when to continue or stop immunosuppression. However, they oversimply the problem to judging "remission" at the time of seeing the patient (which is actually judging disease activity since they define "remission" BVAS of zero) and they overstate the difficulty of doing this clinically. AAV treatment is divided up into "induction" and "maintenance". The point of maintenance remission is to *maintain* the patient in remission. The decision about stopping maintenance therapy is thus more complex than solely judging current disease activity; it also involves prognosticating on what will happen in the future. In other words, a clinically helpful biomarker for therapeutic decisions such as reducing immunosuppression needs go beyond an assessment of the patient's current disease activity and to be able to predict the risk of flare *over some future time window* (e.g. 12-18 months). The authors' claim of having made a clinically transformative paradigm shift thus full short.

This is not to say that the existing results are without merit, but there needs to be reigning back of hyperbole (see also below). Essentially what the authors have done is identified useful additional biomarkers for judging an inactive vs active disease state.

To address this, I suggest the following analysis:

> Re-define remission by adding a “no flare” definition in addition to BVAS. The no flare time window could be 12-24 months depending on flare rate and numbers of patients with follow up data post sample. This should then be used to re-derive the signature of ‘remission’.

<u>Statistical</u>

3) The authors perform feature selection to identify a small number of proteins that discriminate the groups of interest (e.g. active vs remission). They then perform PCA on this small number of selected proteins and claim that separation of these groups on this PCA is evidence that the approach has worked. This logic is flawed and is entirely circular; obviously proteins selected for their discriminative properties will lead to separation of groups of interest on the PCA plot. A PCA plot does not constitute a proper metric for biomarker performance. In addition, performing PCA a handful of proteins (just 7 in Figure 4!) makes little sense in any situation, and even less when there is low correlation between features due to the nature of the LASSO algorithm. The point of PCA is dimension reduction when there is a large number of correlated features i.e. the exact opposite of this situation.

4) Lines 259-263. Use of just two samples in the final section as “evidence” of their pipeline’s effectiveness. We cannot draw any meaningful conclusions from this. The probability of a coin toss classifying the two patients correctly would be: 0.5x0.5 =0.25. This analysis should be removed or performed on a meaningful sample size.

5) The test set in the 80/20 split is very small and vulnerable to the play of chance.

6) The final AUCs (0.98) in the test set using the 7 protein logistic model seems implausibly good. Either this is a statistical “fluke” due to the very small sample size or there is some information leak in the model development.

<u>7) Pre-analytical variation not considered</u>

Time from bleed to spin, time in freezer, and number of freeze thaws are potential sources of non-biological variation and can have major impacts on the proteome (often manifesting in the first few PCs). These factors may differ between groups of interest, and can confound analysis. It’s notable that these confounders were not included in the “confounder” analysis.

The authors should present analyses examining the effect of technical covariates on the data in order that we can assess for any systematic variation related to pre-analytical variation.

E.g. PCAs coloured by:

-time in freezer

-time from bleed to spin

-if applicable, the number of freeze thaws that the samples have undergone

8) Language
The language was often hyperbolic.

Adjectives such as “robust” or “rigorous” were used liberally without clear justification (e.g. there were 6 instances of “rigorous”).

Examples of misuse of “robust”:

-Line 138 “*Principal Component Analysis (PCA) revealed a clear separation of active AAV samples from both HC and remission AAV, indicating robust disease-associated proteomics signatures”*

The PCA plots simply show the major axes of variation in the data. They do not indicate anything about robustness.

-“*These results define a robust, non-redundant, and interpretable 21-protein signature capable of distinguishing remission from active AAV*”

In what way is this 21 protein signature any more “interpretable” than any other signature? Would a different set of proteins be less interpretable? Are 21 proteins any more interpretable than 50 proteins?

In what way is the signature “robust”? Statistically robust means the signature is insensitive to the effects of outliers or violations of assumptions. To claim robustness, we need to see further analysis demonstrating these properties; otherwise remove the hyperbole.

-Lines 116-130
“*This confounder-controlled proteomics framework offers a scalable blueprint for precision diagnostics in immune-mediated disease. Beyond AAV, our approach underscores the potential of targeted multi-marker panels to support clinical decision-making in complex, relapsing- remitting disorders where treatment burden is high and timely identification of remission is critical.”*

This is unsupported conjecture that goes well beyond the actual findings of this paper in terms of AAV, let alone extensions to other diseases. Please remove.

Other specific comments

a) Line 53 “active autoimmune vasculitis” – I appreciate the attempt to make the manuscript accessible to the non-specialist and also sympathize with the unwieldy vasculitis disease nomenclature. However, there are many forms of autoimmune vasculitis and the authors should be specific about which one they are talking about (ANCA-associated vasculitis).

b) Line 58 “We *hypothesized* that the plasma proteome, when rigorously controlled for patient characteristics and clinical confounders, contains biomarkers that reliably diagnose remission in autoimmune vasculitis patients.”

This is not a hypothesis. Please rephrase. This is an unbiased/discovery science paper and there is no need to pretend otherwise. There is great value in unbiased approaches.

c) Section starting line 153
The terminology of confounders is not used correctly.
An association between A and B can arise due:

i) A causing B (causal relationship), or
ii) B causing A (reverse causation), or
iii) A and B being caused by a common confounder C.

The authors describe CRP as a "confounder". This is an incorrect description of the problem they are trying to address. An example of a confounded relationship would be height and breast risk in the adult population; in this case the confounder is sex (since women have a lower mean height than men).

CRP is an excellent read-out of inflammation. However, it lacks specificity for disease activity because CRP can be increased by other causes of inflammation such as infection. This does not mean the "CRP" is a confounder of disease activity.
Rather, what the authors have done is find CRP-independent associations with disease activity rather than remove the effect of "confounding" by CRP. The analysis is worthwhile since CRP lacks specificity; the authors just need to carefully consider what they are describing and adjust the language accordingly.

d) Line 168
" Importantly, the selected proteins showed minimal redundancy:
apart from moderate correlations among three keratins (KRT2, KRT6B, KRT78), the markers
were largely uncorrelated (Extended Data Figure 1e)"

LASSO, by its nature, selects uncorrelated predictors so the statement is "Importantly…" is redundant and inevitable given the choice of method. This type of statement gives the reviewer cause for concern with regard to authors' statistical understanding.

e) Lines 163-168 : I think there may be an over-fitting problem where the authors evaluate a number of supervised learning algorithms and select a method/signature based on performance.

"Using 80/20 train/test data split, we applied different ML algorithms such as random forest, elastic net and
found LASSO (Least Absolute Shrinkage and Selection Operator) regression produced the best-
performing model while maintaining interpretability. LASSO reduced the protein list to 21-
protein features (Figure 2d, Extended Data Figure 1d, Supplementary Table 3), which achieved
an AUC of 0.92 in the test set."

The writing is not entirely clear but raises the possibility that the method and hence signature selection is based on performance in the *test* data. If so, this is a problem and instead the authors should pick the "final" algorithm based on performance in the *training* data and then evaluate their final choice of algorithm in the unseen test data. By selecting the algorithm (and by virtue of this the set of proteins) in the test data there is potential for overfitting. Please clarify how LASSO was defined as best-performing model (training vs test).

I could not see the ROC curve with the 0.92 AUC in either Figure 2d or Extended Data Fig 1d; this would be helpful to see.

Discussion:

f) MS-derived signatures are unlikely to be feasible for clinical translation. A comment on taking the signature forward using affinity-based protein measurement approaches would be useful.

g) The finding that COMP (Cartilage oligomeric matrix protein aka thrombospondin-5) was among the biomarker panel is interesting, and from the Figures, I see it was *reduced* in active disease. COMP has been reported as a biomarker in various conditions, including osteoarthritis where it is elevated (e.g. PMID: 19652761). I note that it is has also recently been reported as part of a biomarker signature in another form of vasculitis (Takayasu arteritis) and intriguingly it was also reduced in active disease (Maughan et al Arthritis Rheumatol 2025 https://doi.org/10.1002/art.43110). This is worthy of comment. Could the authors try to determine the cellular/tissue source of circulating COMP (e.g. from the literature or public databases/atlases and discuss why they think it is *down* in active vasculitis in two distinct diseases (Takayasu arteritis and AAV)?

h) "*More broadly, our study provides a generalizable paradigm for biomarker discovery in complex immunological diseases, illustrating how rigorous control of clinical confounders combined with state-of-the-art proteomics can yield clinically actionable insights."*

The authors have not demonstrated that their results are "clinically actionable" and this sentence should be removed. To be able to make this statement, the authors would need to evaluate their biomarker and a clinical trial and judge whether it enabled safe reduction of immune suppression. To underline this point, previous claims of a transcriptomic biomarker in first described in ANCA-associated vasculitis (McKinney et al Nature Medicine 2010;16(5):586-91 PMID: 20400961) and then by the same group in inflammatory bowel disease (Lee JC et al J Clin Invest. 2011;121(10):4170-9. doi: 10.1172/JCI59255, PMID: 21946256) ultimately showed no clinical utility when evaluated in a clinical trial (Noor et al The Lancet Gastroenterology & Hepatology 2024; 415 – 427).

Figures

- **Fig 1 and Graphical Abstract** Remove the brain logo: this representation is inappropriate since LASSO is not a neural network; it is very much a statistical feature selection tool rather than "AI".

-Fig 2a:
Remove the ellipses from the PCA. These obscure the data.
I would suggest using different symbol to help better discriminate active and remission samples.

-Fib 2b: volcano plots convey very little information without protein labels. Please annotate at least the most significant hits.

-Fig 2e: this needs to deleted; PCA on the 21 proteins selected by the supervised learning method is meaningless.

-Fig 3e: is this AUC generated on the training or the test set. The AUC of 98% seems implausible and suggests information leak or overfitting.

-Fig 4c: please remove
"(c) the PCA shows excellent performance in separating active from remission AAV in the validation cohort."

This analysis of is non-sensical. The authors are doing a PCA on just 7 proteins! Moreover,
These proteins were selected to have low correlation. The point of PCA is for dimension reduction when one has a large number of correlated features i.e. the exact opposite situation to here. Furthermore, PCA shows linear combinations of features that explain the most variation in the data; it is not a suitable method for assessing model performance. The PCA is bound to show separation of groups since the 21 proteins included were selected by the model to i.e. this analysis is entirely circular.

Figure 5. Towards clinical application of the diagnostic 7-protein panel"
The data presented here are a long way from clinical application. Please come up with a less hyperbolic, more factual title.

Response to the reviewers

Reviewer #1

We thank the referee for taking the time to carefully review the manuscript and give constructive comments to improve our study. We appreciate the positive evaluation of our study and addressed all the reviewer's concerns. We performed new analyses, clarified critical points, improved the figures, and edited hyperbolic language to address the reviewer's concerns.

Major points:

Conceptual

1) *The authors have chosen to focus on biomarker discovery/clinical applicability rather than for biological insights into AAV. I felt that the paper would be stronger if the authors used the data to its full extent. In other words, part 1 should be what biological insights can be gained from analysis of the data before presenting biomarker analysis in part 2. Currently the biological insights are cursory/minimal. Perhaps the authors have looked at this and concluded that there is not much novel biology and thus elected not to focus on this aspect, although I must say that I would be surprised if this was the case, particularly given that they see biomarker signatures.*

We agree with the reviewer that the proteomics data contain valuable information for biomarker discovery but could also be explored for biological insights into AAV. We had initially planned to separate the biomarker from the biological exploration but followed the reviewer's suggestion and provide a first insight into plasma protein and pathway regulation focusing on shared and distinct patterns in MPO- and PR3-AAV, including a focused complement-pathway analysis.

We added to the result section on page 6, line 149:

To characterize biological processes associated with active AAV, we examined differentially abundant proteins that were either shared between or specific to active PR3- and MPO-AAV, followed by Gene Ontology (GO) enrichment analysis (Figure 2b–d). Comparison with HC showed that 51% of differential proteins and 29% of enriched GO terms overlapped between active PR3- and MPO-AAV (Figure 2c and Supplementary Table 3).

Shared GO biological processes included coagulation, adhesion, wound response, and gene expression; shared GO cellular components encompassed extracellular matrix, cytoskeleton, and granule structures (Figure 2d). These findings indicate coordinated regulation of inflammation, tissue remodeling, fibrosis, and structural adaptation, consistent with known AAV pathology. Active PR3-AAV patients showed stronger inflammatory signatures than MPO-AAV, in line with higher CRP concentrations, leukocyte counts, and neutrophil numbers (Supplementary Table 1).

Selected GO terms linked to inflammatory and pathophysiological processes were further analyzed using representative proteins from the global proteome (Supplementary Table 2). Median $\log_2$ intensity values confirmed a robust inflammatory response in active AAV, more pronounced in PR3-AAV, that did not normalize in remission despite a BVAS of zero (Extended Data Figure 1g). Proteins associated with blood coagulation and venous thromboembolism (VTE) were strongly regulated in active AAV consistent with reported thromboembolic events during active disease (PMID:33523099) (Extended Data Figure 1h,i). Organ fibrosis signatures were elevated in both AAV serotypes and remained increased in remission (Extended Data Figure 1j). Analysis of complement pathways that provide mechanistic and clinical disease feature (PMID: 33596356; PMID: 19073822) showed activation of the alternative pathway in both active PR3- and MPO-AAV, whereas classical and mannose-binding lectin (MBL) pathway activation was restricted to active PR3-AAV (Figure 2e).

We added to the discussion on page 16, line 404:

Fourth, our global proteome analysis provides insights into pathways and proteins engaged in active AAV. We describe shared but also distinct protein signatures in PR3- and MPO-AAV patients, thereby providing a rationale for future mechanistic and translational studies.

2) *The authors rightly point out the difficulty in decisions about when to continue or stop immunosuppression. However, they oversimply the problem to judging “remission” at the time of seeing the patient (which is actually judging disease activity since they define “remission” BVAS of zero) and they overstate the difficulty of doing this clinically. AAV treatment is divided up into “induction” and “maintenance”. The point*

of maintenance remission is to maintain the patient in remission. The decision about stopping maintenance therapy is thus more complex than solely judging current disease activity; it also involves prognosticating on what will happen in the future. In other words, a clinically helpful biomarker for therapeutic decisions such as reducing immunosuppression needs go beyond an assessment of the patient's current disease activity and to be able to predict the risk of flare over some future time window (e.g. 12-18 months). The authors' claim of having made a clinically transformative paradigm shift thus full short.

This is not to say that the existing results are without merit, but there needs to be reigning back of hyperbole (see also below). Essentially what the authors have done is identified useful additional biomarkers for judging an inactive vs active disease state.

To address this, I suggest the following analysis:

Re-define remission by adding a "no flare" definition in addition to BVAS. The no flare time window could be 12-24 months depending on flare rate and numbers of patients with follow up data post sample. This should then be used to re-derive the signature of 'remission'.

We thank the reviewer for this important critique. We agree that factors in addition to establishing remission (absence of disease activity) at a given time point influence the decision to stop treatment, including the relapse/flare probability. We addressed the reviewer's comment in two parts: (1) an exploratory analysis testing whether our existing 7-protein score predicts 24-month relapse, and (2) a discussion of why re-deriving the signature with a longitudinal remission definition is not feasible with the current dataset, together with a plan for future work.

1) We applied the 40Train 7-protein model to the subset of remission patients from the combined discovery and validation patient cohort with available 24-month follow-up data and tested whether the predicted probability (protein_score) could discriminate patients who subsequently flared from those who remained in remission.

Results are shown in the table below. Among 96 patients sampled in remission who had a 24-month follow-up, 15 (16%) experienced a flare within 24 months. The protein score was not associated with time to flare (Cox per 0.1 increase: HR 0.92 (95% CI 0.76-1.11); p = 0.49). Discrimination for 24-month flare was likewise poor (AUC 0.44 (95% CI 0.29-0.60)).

Analysis	N patients	N flares (24mo)	AUC (95% CI)	p-value
7-protein score vs. flare within 24 months	96	15 (16%)	0.44 (0.29–0.60)	—
Cox regression (per 0.1 increase in protein score)	96	15 (16%)	HR 0.92 (0.76–1.11)	0.49
KM log-rank (median split, illustration only)	96	15 (16%)	—	0.991

AUC = area under the ROC curve; OR = odds ratio from logistic regression; KM = Kaplan–Meier. All analyses applied to the pre-trained model; no refitting was performed.

We added the new data showing that the 7-protein model does not predict future relapses to the Results on page 15, line 368.

2) The reviewer suggests re-deriving the protein signature using a composite remission definition (BVAS = 0 and no flare within 24 months). We considered this approach carefully, but it is statistically not feasible with the available data because we have only 15 flare events as a comparison group (16% event rate) in the remission cohort within 24 months after plasma sampling. Thus, the 'relapser' category is too small to derive a stable protein signature. Flares are now included in tables showing patient characteristics (Supplementary Table 1 and 11)

To specifically address the question of flare prediction, we are currently conducting the prospective PRE-FLARED 2 study, which aims to predict flares using several biomarkers including plasma and urine proteomics (German Clinical Trials Register DRKS00034858). This study is specifically designed with prospective flare endpoints, adequate sample size, and repeated sampling to address the clinically important question raised by the reviewer. We now discuss this approach on page 20, line 486.

We agree with the reviewer that our findings should be framed accurately: our study identifies a validated multi-protein biomarker panel for assessing current disease activity (cross-sectional discrimination of active vs remission at time of sampling), not for prospective relapse prediction. We have revised the manuscript to remove overstated language around clinical decision-making for treatment de-escalation (e.g. page 16, line 394 and page 20, line 506).

<u>*Statistical*</u>

3) *The authors perform feature selection to identify a small number of proteins that discriminate the groups of interest (e.g. active vs remission). They then perform PCA*

on this small number of selected proteins and claim that separation of these groups on this PCA is evidence that the approach has worked. This logic is flawed and is entirely circular; obviously proteins selected for their discriminative properties will lead to separation of groups of interest on the PCA plot. A PCA plot does not constitute a proper metric for biomarker performance. In addition, performing PCA a handful of proteins (just 7 in Figure 4!) makes little sense in any situation, and even less when there is low correlation between features due to the nature of the LASSO algorithm. The point of PCA is dimension reduction when there is a large number of correlated features i.e. the exact opposite of this situation.

We accept the reviewer's criticism and removed the figure and any claims that implied PCA separation is evidence of classifier performance from the manuscript (see also this reviewer's request for figure 4c removal below). We now restrict performance claims to discrimination and calibration metrics in held-out and external datasets.

4) *Lines 259-263. Use of just two samples in the final section as "evidence" of their pipeline's effectiveness. We cannot draw any meaningful conclusions from this. The probability of a coin toss classifying the two patients correctly would be: 0.5x0.5 =0.25. This analysis should be removed or performed on a meaningful sample size.*

We agree with the reviewer's assessment and removed this analysis from the manuscript as requested.

5) *The test set in the 80/20 split is very small and vulnerable to the play of chance.*

We acknowledge that the internal 80/20 split (n = 22; 10 remission, 12 active AAV) is small and therefore subject to sampling variability; however, conclusions regarding generalizability are based on the independent validation cohort (n = 108) and the 60/40 re-split of the combined dataset, which increased sample size for subgroup and comparative analyses. The 80/20 split was used solely as an internal development check, and performance statements in the abstract and discussion rely on the independent validation cohort and the 60/40 re-split analysis. We have clarified this issue in the Discussion page 18, line 447 by adding: „Although the internal 80/20 split was small, the excellent performance of the 7-protein panel was corroborated in the

independent validation cohort and in the 60/40 re-split analysis of the dataset from the combined discovery and validation cohort."

6) *The final AUCs (0.98) in the test set using the 7 protein logistic model seems implausibly good. Either this is a statistical "fluke" due to the very small sample size or there is some information leak in the model development.*

We take this concern seriously and performed several new analyses to assess this issue. We focused on two aspects: (i) whether the AUC reflects information leakage during model development, and (ii) whether the result is a statistical fluke driven by small sample size. We conducted four independent analyses to evaluate whether the high AUC reflects data leakage or genuine signal.

1. Separation and calibration diagnostics in the 80/20 internal split

The 7-protein GLM training set showed quasi-complete separation (EPV = 5.86 in the 80% training subset; 33% of training fitted probabilities at or near 0/1), with >9-fold coefficient differences between GLM/Ridge estimates for several proteins. However, three internal checks demonstrate that this does not explain the observed AUC. First, Firth-penalized logistic regression, robust to separation by design, yielded an identical test AUC of 0.98 (ΔAUC = 0.00), ruling out separation as the cause of the high discrimination. Second, bootstrap-estimated optimism of the training AUC was 0.03, yielding an optimism-corrected estimate of 0.95, consistent with minor memorization only. Third, label-permutation on the 22-patient internal test set confirms the AUC exceeds chance ($p < 0.0005$), ruling out a purely random result. (Supplementary Tabel 9a-c)

Calibration on the 22-patient test set shows a large negative intercept of −2.986, reflecting a systematic shift in absolute probability. Given a small sample size, calibration metrics are expected to be variable and are therefore interpreted with caution. The Hosmer–Lemeshow test ($p < 0.001$) was not used for inference due to its known unreliability in small samples and is reported for completeness only. The calibration slope is 0.93 for the GLM and 1.300 for the Firth model, both consistent with preserved rank-ordering of predictions. Definitive calibration assessment therefore relies on the larger held-out sets described below (pooled cohort Test40, n = 85; external cohort, n = 108).

2. Independent external validation cohort (n = 108)

The 7-protein model, trained exclusively in the full discovery cohort, was applied without modification to the external validation cohort (n = 108, 54 events), which was never used at any stage of model development, feature selection, or threshold tuning. Label-permutation testing confirms that the validation AUC is significantly above chance (permutation $p < 0.001$) (Supplementary Tabel 12a-c). The validation AUC of 0.94 (95% CI: 0.90–0.98), achieved in an entirely independent population of 108 patients that was never involved in model development, provides strong evidence that the discriminative panel performance reflects genuine protein signal rather than a statistical artefact of the small internal split.

To further assess whether separation-induced coefficient inflation drives discrimination rather than genuine protein signals, we refit the same 7-protein panel on discovery training data using Ridge regularization (alpha = 0, lambda selected by 10-fold CV). Ridge shrinks coefficients by a median factor of approximately 10× relative to the GLM (e.g. CLEC3B: GLM −2.41 vs. Ridge 0.19; MCAM: GLM 7.01 vs. Ridge 0.66), eliminating quasi-complete separation entirely (0% extreme training predictions vs. 33% for the GLM). Despite this substantial coefficient shrinkage, the external cohorts AUC yielded 0.95 (95% CI: 0.91–0.98), nearly identical to the GLM AUC of 0.94. Discrimination is therefore driven by the protein signal, not by inflated coefficients. We note that Ridge model selection was informed by calibration performance and therefore constitutes a sensitivity analysis rather than an independent validation; the GLM remains the primary model for all discrimination comparisons. Ridge results have been added as supplementary tables (Supplementary Table 12) and figures (Extended Data Figure 4a,b) in the revised manuscript.

3. Pooled 60/40 re-split with refit-permutation overfitting test

To further assess robustness, we performed a pre-defined 60/40 re-split of the pooled dataset (Train60: n = 127; Test40: n = 85), enabling evaluation across both cohorts. The model was refit on Train60 and applied once to Test40. The observed AUCs were 0.975 (train) and 0.974 (test), with a difference of 0.001. A refit-permutation procedure (2,000 resamples) yielded $p = 0.744$, indicating no detectable inflation of test performance relative to training performance.

4. Concentration-based model as independent implementation check

As a step toward clinical implementation, we additionally evaluated a concentration-based version of the same 7-protein panel, in which TQL-based PRM-MS signal intensities were converted to absolute plasma concentrations and subsequently variance-stabilization normalized (VSN) to account for differing concentration scales (Methods; Supplementary Table 7c). This model achieved an AUC of 0.91 (95% CI: 0.85–0.97) on Test40 (n = 84), with a calibration slope of 0.804. We note post-hoc that the concentration model shows better calibration than the normalized intensity model, a finding we did not anticipate and report transparently here. The modest reduction in AUC (0.91 vs. 0.97) is expected when moving from optimized normalized intensities to absolute concentrations. The 7-protein panel performance, however, remains reliable in either implementation.

Taken together, these four lines of evidence, the Firth-penalized replication of the internal AUC, the permutation-validated independent external validation, the permutation-confirmed absence of a train/test gap in the 60/40 re-split, and the concentration-based model on held-out data consistently demonstrate high discriminative performance regardless of estimator, regularization strategy, or implementation, confirming that the seven proteins themselves, not modelling artefact (such as overfitting, separation, or information leakage), are the source of the signal.

We also acknowledge that formal calibration diagnostics on the training split were not pre-specified; the original study design focused on discrimination as the primary metric, and this cannot be corrected retroactively.

All calibration analyses, Ridge regularization sensitivity analyses, and overfitting permutation tests reported here were conducted after submission in response to this review. They are transparently labelled as post-hoc diagnostics, not pre-specified confirmatory analyses, and have been incorporated as supplementary material in the revised manuscript with corresponding updates to the Results on page 10, line 247, page 12, line 282, page 13, line 316, Discussion on page 18, line 447, and Methods on page 35, line 880.

7) *__Pre-analytical variation not considered__*

Time from bleed to spin, time in freezer, and number of freeze thaws are potential sources of non-biological variation and can have major impacts on the proteome (often

manifesting in the first few PCs). These factors may differ between groups of interest, and can confound analysis. It's notable that these confounders were not included in the "confounder" analysis.

The authors should present analyses examining the effect of technical covariates on the data in order that we can assess for any systematic variation related to pre-analytical variation.

E.g. PCAs coloured by:

-time in freezer

-time from bleed to spin

-if applicable, the number of freeze thaws that the samples have undergone

We thank the reviewer for drawing attention to pre-analytical and technical factors that can affect proteome analyses. We performed new analyses to examine the effect of three documented technical covariates on the global proteome of the exploratory cohort. These factors were (i) time in freezer, defined as the interval in months between blood draw and MS measurement; (ii) MS plate/batch identity; and (iii) MS run order. Freeze-thaw cycles were limited to a maximum of two but not further specified (now stated in Methods on page 26, line 648).

In our new analyses, principal component analysis of (i), (ii) and (iii) was conducted on the centered and scaled 605-protein matrix (Extended Data Figure 1b-f). We evaluated associations between the first three principal components (PC1–PC3) and the recorded technical covariates (freezer storage duration in months, MS run order, and MS plate/batch). Linear models demonstrated negligible explanatory power of freezer storage time for PC1 ($R^2 = 0.0053$, $p = 0.461$), PC2 ($R^2 = 0.0125$, $p = 0.259$), and PC3 ($R^2 = 0.0042$, $p = 0.515$). Similarly, MS run order showed minimal association with PC1 ($R^2 = 0.00003$, $p = 0.956$), PC2 ($R^2 = 0.0026$, $p = 0.611$), and PC3 ($R^2 = 0.0008$, $p = 0.778$). For MS plate/batch (tested via ANOVA), no significant association was observed with PC1 ($p = 0.967$), PC2 ($p = 0.875$), or PC3 ($p = 0.177$). Thus, none of the recorded technical variables explained a meaningful proportion of variance in the leading principal components.

We next performed a joint PERMANOVA (adonis2; Euclidean distance on the scaled protein matrix; 999 permutations; marginal tests) including disease status, freezer time,

MS plate, and MS run order. Disease status explained 34.2% of the total multivariate variance ($R^2 = 0.342$, $F = 52.38$, $p = 0.001$). In contrast, freezer storage time explained 0.8% of the variance ($R^2 = 0.008$, $F = 1.26$, $p = 0.259$), MS plate explained 1.2% ($R^2 = 0.012$, $F = 1.80$, $p = 0.123$), and MS run order explained 1.0% ($R^2 = 0.010$, $F = 1.54$, $p = 0.157$). None of the technical covariates reached statistical significance in the multivariate model.

Taken together, these results indicate that disease activity represents the dominant source of structure in the 605-protein plasma proteome, whereas recorded technical variables contribute only a very small proportion of variance (≤1.2%) and do not materially influence the observed separation between active and remission samples. We acknowledge, however, that certain pre-analytical parameters (e.g., exact bleed-to-spin time and detailed freeze–thaw histories beyond protocol limits) were not systematically recorded and are therefore explicitly noted now as a limitation.

We added to the Results on page 6, line 144: "PCA and PERMANOVA of the 605-protein plasma proteome showed that freezer time, MS plate, and MS run order each explained ≤1.2% of total variance, whereas disease status explained 34.2% ($p = 0.001$), indicating that technical covariates did not materially shape the dominant proteomic structure (Extended Data Figure 1b-f)."

We added to the limitations in the Discussion on page 19, line 461: "Unrecorded pre-analytical variables could have affected the plasma proteome. Although biosampling was not strictly harmonized between the exploratory and validation cohorts, a stable protein signature was confirmed in both cohorts."

8) *__Language__*

The language was often hyperbolic. Adjectives such as "robust" or "rigorous" were used liberally without clear justification (e.g. there were 6 instances of "rigorous").

Examples of misuse of "robust":

-Line 138 "Principal Component Analysis (PCA) revealed a clear separation of active AAV samples from both HC and remission AAV, indicating robust disease-associated proteomics signatures"

The PCA plots simply show the major axes of variation in the data. They do not indicate anything about robustness.

-"These results define a robust, non-redundant, and interpretable 21-protein signature capable of distinguishing remission from active AAV"

In what way is this 21 protein signature any more "interpretable" than any other signature? Would a different set of proteins be less interpretable? Are 21 proteins any more interpretable than 50 proteins?

In what way is the signature "robust"? Statistically robust means the signature is insensitive to the effects of outliers or violations of assumptions. To claim robustness, we need to see further analysis demonstrating these properties; otherwise remove the hyperbole.

-Lines 116-130

"This confounder-controlled proteomics framework offers a scalable blueprint for precision diagnostics in immune-mediated disease. Beyond AAV, our approach underscores the potential of targeted multi-marker panels to support clinical decision-making in complex, relapsing- remitting disorders where treatment burden is high and timely identification of remission is critical."

This is unsupported conjecture that goes well beyond the actual findings of this paper in terms of AAV, let alone extensions to other diseases. Please remove.

We agree with the reviewer and have edited the text accordingly. We removed or replaced terms such as "robust/rigorous/clinically transformative" where they were not directly supported by the data. We deleted "interpretable" and the paragraph on the "transferable blueprint" as requested and restrict conclusions to what the data demonstrate (cross-sectional classification of active vs remission at sampling with external validation).

Other specific comments

a) *Line 53 "active autoimmune vasculitis" – I appreciate the attempt to make the manuscript accessible to the non-specialist and also sympathize with the unwieldy vasculitis disease nomenclature. However, there are many forms of autoimmune*

vasculitis and the authors should be specific about which one they are talking about (ANCA-associated vasculitis).

The reviewer is right, and we have specified that we studied ANCA-associated vasculitis as suggested, including in (now) line 50.

b) *Line 58 "We hypothesized that the plasma proteome, when rigorously controlled for patient characteristics and clinical confounders, contains biomarkers that reliably diagnose remission in autoimmune vasculitis patients."*

This is not a hypothesis. Please rephrase. This is an unbiased/discovery science paper and there is no need to pretend otherwise. There is great value in unbiased approaches.

We followed the reviewer's suggestion and have deleted the hypothesis part on page 3, line 56 and on page 5, line 112.

c) *Section starting line 153*

The terminology of confounders is not used correctly. An association between A and B can arise due:

i) A causing B (causal relationship), or

ii) B causing A (reverse causation), or

iii) A and B being caused by a common confounder C.

The authors describe CRP as a "confounder". This is an incorrect description of the problem they are trying to address. An example of a confounded relationship would be height and breast risk in the adult population; in this case the confounder is sex (since women have a lower mean height than men).

CRP is an excellent read-out of inflammation. However, it lacks specificity for disease activity because CRP can be increased by other causes of inflammation such as infection. This does not mean the "CRP" is a confounder of disease activity.

Rather, what the authors have done is find CRP-independent associations with disease activity rather than remove the effect of "confounding" by CRP. The analysis is

worthwhile since CRP lacks specificity; the authors just need to carefully consider what they are describing and adjust the language accordingly.

We thank the reviewer for this precise and important correction. The reviewer is entirely right: CRP is not a confounder of disease activity in the epidemiological sense. What we did, and should have described more accurately, is identify protein–disease activity associations that persist independently of CRP, eGFR, and other clinical variables, not remove the effect of causal confounding.

We have revised the terminology throughout the manuscript accordingly. We no longer describe CRP or eGFR as 'confounders' of disease activity. Instead, we use language such as 'CRP-independent associations with disease activity' and 'proteins whose disease signal was not statistically reducible to co-variation with CRP, eGFR, or other measured clinical variables.'

In the Methods, we retain the software's internal terminology (status labels OK_sd, C, AD etc.) since these are defined by the MetaDeconfoundR framework, but we now provide a better description of the methodology beginning on page 31, line 786, and explicitly state that in our application these labels denote statistical reducibility within the observed metadata structure, not causal confounding in the epidemiological sense (Methods, page 32, line 804).

d) *Line 168*

“Importantly, the selected proteins showed minimal redundancy: apart from moderate correlations among three keratins (KRT2, KRT6B, KRT78), the markers were largely uncorrelated (Extended Data Figure 1e)”. LASSO, by its nature, selects uncorrelated predictors so the statement is “Importantly…” is redundant and inevitable given the choice of method. This type of statement gives the reviewer cause for concern with regard to authors’ statistical understanding.

We thank the reviewer for this comment and agree that, given the use of LASSO, a low degree of redundancy among selected predictors is expected and should not be framed as an independent statistical result. We have removed “Importantly” in this context (page 8, line 200) and revised the text to present the correlation structure of the selected proteins as a descriptive context only (page 9, line 213) without implying additional methodological advantage.

e) *Lines 163-168: I think there may be an over-fitting problem where the authors evaluate a number of supervised learning algorithms and select a method/signature based on performance.*

"Using 80/20 train/test data split, we applied different ML algorithms such as random forest, elastic net and found LASSO (Least Absolute Shrinkage and Selection Operator) regression produced the best- performing model while maintaining interpretability. LASSO reduced the protein list to 21-protein features (Figure 2d, Extended Data Figure 1d, Supplementary Table 3), which achieved an AUC of 0.92 in the test set."

The writing is not entirely clear but raises the possibility that the method and hence signature selection is based on performance in the test data. If so, this is a problem and instead the authors should pick the "final" algorithm based on performance in the training data and then evaluate their final choice of algorithm in the unseen test data. By selecting the algorithm (and by virtue of this the set of proteins) in the test data there is potential for overfitting. Please clarify how LASSO was defined as best-performing model (training vs test).

I could not see the ROC curve with the 0.92 AUC in either Figure 2d or Extended Data Fig 1d; this would be helpful to see.

We thank the reviewer for raising this point. To clarify: the initial feature reduction model (random forest, elastic net and LASSO) was conducted during an early exploratory phase on the raw proteomics dataset but consistently surfaced CRP-correlated inflammatory proteins. We therefore adopted the MetaDeconfoundR as a first step to the full proteome and subsequently applied LASSO, as a single pre-specified feature reduction method trained exclusively on the 80%, without looking into the 20% test set.

We realize that our initial description was misleading and removed the reference to random forest and elastic net (page 8, line 195). Consequently, we provide a better description of the workflow starting on page 9, line 207.

Regarding the 21-protein AUC of 0.92: We wish to be transparent that this figure was generated post-hoc for manuscript reporting purposes. A GLM of the 21 LASSO-selected proteins was fitted on the 80% training data and evaluated in the 20% held-out test set. This was done after the 7-protein panel had already been finalized and

was not used to inform any modelling decision. Given the small test set (n = 22), the 95% CI is wide (0.81–1.00) and should be interpreted with caution. We have clarified this issue explicitly in the revised manuscript (Methods, page 35, line 880 and show the figure in Extended Data Figure 2e. The call out was added to the appropriate Result section page 9, line 221.

Discussion:

f) *MS-derived signatures are unlikely to be feasible for clinical translation. A comment on taking the signature forward using affinity-based protein measurement approaches would be useful.*

As requested, we added a short paragraph discussing translational measurement options (page 19, line 480). While PRM-MS is well suited for multiplexed, antibody-independent quantification, future clinical deployment could also be supported by alternative targeted platforms (e.g., immunoassays or affinity-based multiplex assays) following analytical validation and inter-laboratory standardization.

g) *The finding that COMP (Cartilage oligomeric matrix protein aka thrombospondin-5) was among the biomarker panel is interesting, and from the Figures, I see it was reduced in active disease. COMP has been reported as a biomarker in various conditions, including osteoarthritis where it is elevated (e.g. PMID: 19652761). I note that it is has also recently been reported as part of a biomarker signature in another form of vasculitis (Takayasu arteritis) and intriguingly it was also reduced in active disease (Maughan et al Arthritis Rheumatol 2025 https://doi.org/10.1002/art.43110). This is worthy of comment. Could the authors try to determine the cellular/tissue source of circulating COMP (e.g. from the literature or public databases/atlases and discuss why they think it is down in active vasculitis in two distinct diseases (Takayasu arteritis and AAV)?*

We thank the reviewer for this important detail on COMP. We have expanded the discussion on page 17, line 427. It now reads: We observed reduced plasma COMP levels in active AAV patients. Increased COMP levels were reported in osteoarthritis and rheumatoid arthritis and reduced levels recently in Takayasu arteritis. COMP is an extracellular matrix glycoprotein that is thought to contribute to endochondral

ossification. However, it is found not only in cartilage, synovium and bone from where it is released during arthritis (PMID:19652761, PMID: 39672391) but also in the vascular wall (PMID: 41009743). Vascular smooth muscle cells (VSMCs) produce and release COMP into the plasma and the vessel wall where it maintains vascular homeostasis. Conceivably, vasculitis causes VSMCs injury and dedifferentiation resulting in decreased plasma concentrations and possibly contributing to vascular injury. Reduced COMP was recently reported in another vasculitis entity, namely in Takayasu's suggesting shared disease mechanisms (PMID: 39817309).

h) *"More broadly, our study provides a generalizable paradigm for biomarker discovery in complex immunological diseases, illustrating how rigorous control of clinical confounders combined with state-of-the-art proteomics can yield clinically actionable insights."*

The authors have not demonstrated that their results are "clinically actionable" and this sentence should be removed. To be able to make this statement, the authors would need to evaluate their biomarker and a clinical trial and judge whether it enabled safe reduction of immune suppression. To underline this point, previous claims of a transcriptomic biomarker in first described in ANCA-associated vasculitis (McKinney et al Nature Medicine 2010;16(5):586-91 PMID: 20400961) and then by the same group in inflammatory bowel disease (Lee JC et al J Clin Invest. 2011;121(10):4170-9. doi: 10.1172/JCI59255, PMID: 21946256) ultimately showed no clinical utility when evaluated in a clinical trial (Noor et al The Lancet Gastroenterology & Hepatology 2024; 415 – 427).

We agree and removed the statement on page 20, line 500 that implied that the findings are already "clinically actionable". We now frame clinical utility as a proof-of-concept supported by external validation and decision-curve analysis, and we explicitly state that prospective clinical trials are needed to evaluate whether the panel can safely inform clinicians to de-escalate treatment.

Figures

- ***Fig 1 and Graphical Abstract**: Remove the brain logo: this representation is inappropriate since LASSO is not a neural network; it is very much a statistical feature selection tool rather than "AI".*

Done as suggested.

-***Fig 2a**: Remove the ellipses from the PCA. These obscure the data.*

I would suggest using different symbol to help better discriminate active and remission samples.

We believe that the ellipses help to comprehend and visualize the separation of the clinical groups despite the high biological variability. However, we removed the ellipse fillings to increase the readability of the plot.

-***Fig 2b**: volcano plots convey very little information without protein labels. Please annotate at least the most significant hits.*

Done as suggested. Please note that these data are now shown in Figure 2f and new Figure 2b.

-***Fig 2e**: this needs to be deleted; PCA on the 21 proteins selected by the supervised learning method is meaningless.*

Done as suggested

-***Fig 3e**: is this AUC generated on the training or the test set. The AUC of 98% seems implausible and suggests information leak or overfitting.*

We would like to refer the reviewer to our response to comment 6. We hope that we were able to clarify any confusion that may have occurred originally.

-***Fig 4c**: please remove "(c) the PCA shows excellent performance in separating active from remission AAV in the validation cohort."*

This analysis of is non-sensical. The authors are doing a PCA on just 7 proteins! Moreover,

These proteins were selected to have low correlation. The point of PCA is for dimension reduction when one has a large number of correlated features i.e. the exact opposite situation to here. Furthermore, PCA shows linear combinations of features that explain the most variation in the data; it is not a suitable method for assessing model performance. The PCA is bound to show separation of groups since the 21 proteins included were selected by the model to i.e. this analysis is entirely circular.

We agree and have removed the figure as suggested.

***Figure 5**. Towards clinical application of the diagnostic 7-protein panel"*

The data presented here are a long way from clinical application. Please come up with a less hyperbolic, more factual title.

We have edited the legend title. It now reads: "Performance of the diagnostic 7-protein panel in the combined cohort."

Reviewer #2

This is an impressive study. Although I'm not an expert in proteomics nor in some of the data-analysis techniques, in the end it really doesn't matter how the authors came up with their high-performing 7-protein assay, because it also performed very well in an independent cohort. The AUC is far higher than the frequent benchmark of 0.8 as a minimum for having value, and the positive likelihood ratio, if they had calculated it, would be well over 10, another common benchmark for clinical utility. The authors are correct that this study addresses a clinically important need in which research advances over many years have not yet led to new clinically useful tests.

We thank the referee for the positive evaluation of our study and the encouraging comments. We addressed both comments of this reviewer.

I only have two sets of comments:

***1.** Confounder-aware feature selection. The authors excluded proteins that were significantly associated with CRP, GFR, or "other patient metadata." I think these*

should be specified, sorry if I missed that in the supplementary tables (it isn't in the methods). Based on supplementary tables 1 and 6, lack of association with either hematocrit/hemoglobin or platelets would be particularly important. In the unlikely event that that wasn't done, that should be added as a limitation. It would be preferable to show correlation plots with GFR rather than just CRP (extended data fig 2), and also Hct/Hgb and platelets if done. In the process of looking for this, I noticed that the call-outs to Extended Data Figure 1 don't match that figure, including a call-out to 1e, when the figure only has a-d. I also didn't see a call-out to Extended Data Figure 2, which I expect was intended to be referenced in that section on page 8.

We apologize for the insufficient detail. MetaDeconfoundR was applied to the full 605-protein dataset using nested linear model likelihood-ratio testing with the following covariates: age, sex, eGFR, CKD, creatinine, CRP, hemoglobin, hematocrit, leukocytes, neutrophils, platelets, ANCA (continuous ELISA titer), and organ involvement. Proteins were excluded from panel development if their disease association was classified as confounded (status ≠ OK_sd (association not reducible to any covariate), OK_nc (no other significant covariates present) or AD (ambiguously deconfounded) after FDR correction. We added a more detailed description to the methods on page 32, line 789.

The full MetaDeconfoundR output for all 605 proteins and all covariates is provided in Supplementary Table 4 of the revised manuscript. Supplementary Table 4 table shows all seven proteins are classified as OK_sd, indicating that their association with disease activity cannot be statistically explained by CRP, eGFR, hemoglobin, hematocrit, platelet count, or ANCA titer within the observed metadata structure Notably, for five of the seven proteins (AHSG, COMP, F9, MCAM, MRC1), the protein–CRP association itself is classified as C: disease_numeric, indicating that the observed correlation with CRP is attributable to shared disease activity rather than the proteins acting as CRP surrogates. In contrast, LRG1 and CLEC3B show AD status with CRP, consistent with their established roles in acute-phase and inflammatory responses. Importantly, however, their associations with disease activity remain non-reducible, supporting their inclusion in the disease activity-associated protein panel.

In addition, we include a visualization of the MetaDeconfoundR results for the 21 LASSO-selected candidate proteins (Extended Data Figure 2d). Within this heatmap,

the seven panel proteins and their associations with clinical covariates can be directly inspected.

***2.** Effects of treatment. I don't understand Figure 4d, which is essential for saying that possible direct effects of treatment were explored and that the finding was similar regardless. The legend, and the acocmpanying text, indicate that only samples from patients with active disease were used. Is so, what is the dichotomous outcome being modeled with an AUC curve? If it isn't active disease vs remission, then that needs to be clear in both the text and the legend, and I'm skeptical that any other comparison would show that treatment has negligible direct effects on assay results. Even if the problem is simply an error in not reporting that samples from patients in remission were also used in this analysis, I still think it would be important to add as a limitation in the discussion something like, "The study was too small, and the diseases and details of treatment are too heterogeneous, to definitively rule out effects of medications on biomarker concentrations independent of disease activity."*

We apologize for any confusion with old Figure 4d, now Figure 4c. We now provide a better explanation in the corresponding figure legend that hopefully clarifies what the analysis revealed. The reviewer is correct; the figure depicts the 7-protein panel performance (AUCs) for diagnosing remission by comparing the results of the remission group to the three active disease groups with respect to their treatments. The ROC curves demonstrate that the biomarker panel performed excellent when remission patients were compared with either of the three subgroups of patients with active AAV, namely completely treatment naïve, glucocorticoids (GC) only, GC plus cyclophosphamide/rituximab patients. This stratified analysis supports consistency of performance across these treatment strata but does not definitively prove the absence of drug effects. The last sentence was added to the limitations in the discussion section on page 19, line 469.

Reviewer #3

Overall, well planned study with powerful tools. I agree with your thought process and is thoroughly executed. I have minor comments to point out.

We thank the referee for the very positive evaluation of our study. The referee had only minor comments that we have addressed in the revised manuscript.

(1) *It seems that, throughout the paper, the purpose of the search for biomarkers is to "diagnose" AND "evaluate the activity". In fact, MPO and PR3 levels can be used for both diagnosis and evaluation of activity. However, the title of this paper is "towards a proteomic plasma biomarker panel for diagnosing vasculitis remission". In the paper, few more sentences need to be added to clarify that not only seven-biomarkers can help to evaluate the AAV activities but also aid in diagnosis.*

We agree that MPO- and PR3-ANCA are very helpful in establishing the diagnosis of AAV. ANCA, however, are less accurate in evaluating the disease activity status of AAV. Diagnosing remission is where we see the main contribution of the 7-protein panel. In our study, the performance of ANCA was inferior compared to the 7-protein panel (see Figure 5b). Since we did not perform studies in AAV disease controls, we feel that we should not comment on performance of the 7-protein panel as a diagnostic tool for diagnosing AAV. We added this issue to the limitation section in the discussion on page 20, line 489. It now reads: "In addition, whether the 7-protein panel helps with diagnosing AAV needs to be explored by comparisons with appropriate disease controls."

(2) *(line 90-92): In practice, there are AAV patients whose laboratory results do not match with actual disease activities. However, I believe that majority of them actually show match between the lab results and disease activities. Please provide approximate percentage of patients whose disease activities are not represented by currents biomarkers (ANCA and CRP).*

We agree that particularly diagnosing AAV remission becomes more challenging when patients have positive ANCA or elevated CRP. Among 105 patients in clinical remission, 69 (65.7%) remained ANCA-positive and 21 (20.0%) had elevated CRP. Overall, 77 of 105 remission patients (73.3%) had at least one conventional biomarker (ANCA or CRP) still elevated, and 13 (12.4%) had both markers elevated despite BVAS-defined remission. These data demonstrate that a substantial proportion of remission patients continue to display markers associated with disease activity. This discordance underscores the limited specificity of CRP and ANCA for defining true clinical remission at the individual-patient level and highlights the clinical need for more robust, multi-protein biomarkers. These data are now included in Results on page 14, line 336.

__(3)__ In few supplementary tables, EGPA is listed under MPO-AAV. In fact, large portion of EGPA patients have MPO titers and may play pathogenic roles. However, MPA and EGPA are clearly different entities, and it needs to be differentiated. Please clarify in the writing.

The reviewer is right; a small number, namely 3/39 MPO-AAV patients of the exploratory and 2/55 MPO-AAV patients of the validation cohort, were diagnosed with EGPA. We agree, EGPA is likely to have common but also distinct disease mechanisms compared to MPA. As suggested, we now discuss this issue in the discussion section on page 19, line 470.

Reviewer #4

I co-reviewed this manuscript with one of the reviewers who provided the listed reports. This is part of the Nature Communications initiative to facilitate training in peer review and to provide appropriate recognition for Early Career Researchers who co-review manuscripts.

We thank the reviewer for co-reviewing the manuscript with an Early Career Researcher (Reviewer #1) and for thoughtful comment to improve our manuscript.

(Remarks on code availability): It appears that the dataset/record at Zenodo with DOI 10.5281/zenodo.16927900 indicates "Files: Restricted" — the files are not publicly accessible without permission. Could you please grant reviewer access or send the code repository link?

The Zenodo repository experienced a temporary outage. We have additionally made all code and model objects available at https://github.com/Theda-sys/vasculitis-remission-proteomics for immediate reviewer access.

Reviewer #5

The need for better biomarkers in AAV in clear, as the authors state, treatment is toxic and there is often uncertainty in the mind of the clinician as to when remission has occurred, the implication being that treatment can be reduced. Using plasma proteins,

a panel of 7 has been identified which discriminate between active and remission states and perform better than existing biomarkers. This panel could readily be translated to a clinically useful tool.

We thank the referee for carefully reviewing the manuscript and constructive comments and suggestions to improve our study. As requested, we performed new analyses, clarified critical points, and edited the manuscript accordingly.

1. *A thorough and robust approach to identification and validation of protein biomarkers in AAV active and remission patients and healthy controls. Resulting in a 7 panel protein set. Important strengths are:*

a) Confounder-aware selection: explicit deconfounding vs CRP/eGFR before modeling.

b) External validation: independent cohort + robustness across treatment strata (naïve, GC, GC+IS).

c) Interpretability & utility: parsimonious GLM, benchmarking vs CRP/ANCA, and decision-curve analysis.

The presented description of the methodology and Figures are of a high standard, accessible to the reader and compliment the report.

We thank the reviewer for the positive evaluation of our manuscript and the encouraging comments on the strength of the study.

2. *There is potential weakness over the 'remission' definition. Because AAV is typically a relapsing disease, in many patients 'remission' is disease suppression on therapy, then relapse occurs on reduction of therapy. Guidelines have defined 'on treatment' and 'off treatment' remission for example.*

We agree that the issue of remission on versus off treatment is clinically important in AAV. We now added this information to our patient cohort description (Supplementary Tables 1 and 11). We defined remission off-treatment as less than 7.5 mg/d prednisolone and no other immunosuppressant currently or within the last 6 months. As off-treatment remission occurred exclusively in the Berlin cohort, with no

corresponding cases in Prague, external validation of model performance in on- and off- treatment groups was not possible.

3. *Although there may appear to be control of systemic inflammation - CRP, ANCA, clinical assessment, patient symptoms - there can be ongoing lower level 'activity' in organs, for example in the kidney. This is often referred to as 'grumbling disease' although it can be hard to define or detect without tissue biopsy. Further work is required to understand to what extent this panel can 'detect' such lower level disease activity, yet these are the manifestations that will drive relapse and may drive chronic tissue injury.*

I see the issues in comments 2 and 3 as being the main concerns I have with this paper and would like to see them further addressed under limitations or discussion of future studies.

The reviewer is right in pointing out that low-level AAV activity, referred to as “grumbling disease”, is not easy to capture. The performance of the 7-protein panel in this AAV subgroup should be explored in future studies. We followed the reviewer’s suggestion and have included this issue in the discussion of future studies on page 20, line 490.

4. *Treatment is always a major confounder, especially glucocorticoids. It is notable that rituximab was widely used for induction which helps control heterogeneity but of the 'active' patients some were on therapy and some not, and my guess is that glucocorticoid doses would have been variable. Further, for the 'remission' cohort some were on glucocorticoids and some not. Is there a way of controlling for glucocorticoid confounding?*

The reviewer points out correctly that treatments, including glucocorticoids (GC) varied in the (early) active patient cohort depending on the time of plasma sampling. We tried to assess the effect of this early treatment variation in active AAV patients by assessing the AUCs of three groups separately, namely completely treatment naïve, GC only, GC plus (cyclophosphamide/rituximab). Treated patients were sampled within 10 days after treatment had started (median 3.0 days). In this short period, GC doses were high, usually initially 250-500 mg daily pulses for 3 consecutive days, followed by 1 mg/kg body weight/day. Thus, during this short initial time, the GC administration was

relatively homogenous. We found that the 7-protein panel performed similar accurately when comparing remission patients with active AAV patients belonging to either of the three induction treatment groups (Figure 4e).

With respect to remission on- and off-treatment, we refer to our response to the reviewer comment #2. However, because of the retrospective treatment analysis it was not possible to specifically evaluate glucocorticoid effects.

These issues were now discussed under limitations in the discussion (page 19, line 469).

5. *CRP and GFR are pre-defined confounders. What about ANCA ? I accept that the AAV patient groups were clearly divided by ANCA serotype, but ANCA are one element of pathogenesis and ANCA binding levels vary between patients both in their initial levels and in their response to treatment. Also, persisting ANCA has been associated with higher relapse risk.*

We agree with this reviewer that ANCA are mechanistically involved in AAV and harbor clinically relevant information depending on titer dynamic, persistence, and reappearance. We included ANCA serotype and titer in our study but were not able to retrospectively retrieve information on persisting versus reoccurring ANCA.

However, the reviewer is correct, like CRP and eGFR, ANCA titer was included as a covariate in MetaDeconfoundR together with additional covariates, see also our response to Reviewer #2, where the full covariate list and methodology are detailed. Importantly, our analysis shows that all seven panel proteins are classified as OK_sd for disease activity in the global dataset, indicating that their disease association is not statistically reducible to ANCA or any other measured covariate. We now include this information in the Supplementary Table 4 and Extended Data Figure 2d.

6. *As yet, there have not been studies in disease controls, i.e. other systemic inflammatory diseases, or in AAV patients with co-morbidities causing inflammation, such as infection or certain drug toxicities.*

The reviewer is right that we did not perform studies in AAV disease controls and AAV patients with specified co-morbidities. We agree, it would be interesting to test the 7-

protein panel performance in other inflammatory and autoimmune diseases. We have added this issue to the future study section in the discussion on page 20, line 489.

7. *I would also like to see validation against organ specific features - extending the discussion over grumbling versus systemic disease. The obvious organ to do this would be the kidney and to compare the plasma protein signal with urine and possibly histology. This lies outside the aims for this report but could be mentioned as a question to be addressed in the future.*

We agree with the reviewer that the comparison of the plasma 7-protein panel, urine proteomics, and kidney histology would be interesting but is beyond the scope of the current study. As suggested, we included this issue as a question to be addressed in the future (page 20, line 486).

<u>*Minor points:*</u>

1. Add uncertainty + calibration (one small table/figure). Please provide 95% CIs for AUC, sensitivity, specificity, PPV/NPV and report calibration slope/intercept (or Brier score) with a small calibration plot. Rationale: Completes TRIPOD items and strengthens clinical credibility without re-analysis.

We thank the reviewer for this important suggestion. In accordance with TRIPOD recommendations, we have expanded the model evaluation to include uncertainty and calibration metrics. Specifically, we now report 95% confidence intervals for all AUC in the Results, as well as for sensitivity, specificity, PPV and NPV. In addition, we provide Brier scores and calibration slope/intercept estimates in newly created Supplementary Tables together with a calibration plot in Figure 5a.

2. Stratify performance by kidney functon(one supplementary table). Add AUC (95% CI) stratified by eGFR (e.g., ≤45 vs >45 mL/min/1.73 m²). Rationale: They already deconfounded against eGFR; this simple stratification confirms robustness across renal impairment (they've already shown treatment-stratified performance).

Although we initially excluded proteins that were eGFR–associated, we additionally assessed, as suggested by the reviewer, robustness across renal function. We stratified the external validation cohort from Prague by eGFR (≤45 vs >45 mL/min/1.73 m²) and re-evaluated the 7-protein model performance (new Figure 4b). In patients from the validation cohort with eGFR ≤45, the AUC was 0.94 (95% CI 0.89–0.99), and in those with eGFR >45, the AUC was 0.89 (95% CI 0.77–1.00). DeLong test did not show a statistically significant difference between strata (p = 0.43). Because the higher-eGFR group is small and its confidence interval wide, we treat these stratified results as descriptive only. We added the new data to Results page 12, line 291 and present them in Supplementary Table 13.

3. One-sentence workflow clarification. State explicitly that all feature selection/tuning were performed within training data only, and that the held-out test set + external cohort were untouched until final evaluation; confirm the operating threshold was fixed from training. Rationale: Avoids any concern about data leakage; aligns with what they already did.

We thank the reviewer for this suggestion and apologize for any confusion with the workflow description. We now confirm explicitly: all feature selection (MetaDeconfoundR on the full discovery cohort, followed by LASSO and Boruta within the 80% discovery training split) was performed without any access to held-out data. The 20% discovery test split, the external validation cohort, and the pooled Test40 partition were each untouched until final evaluation, and no feature, parameter, or threshold was modified based on their results. The operating threshold (0.5 probability) was fixed prior to any validation. No hyperparameter optimization was performed; LASSO and Boruta were applied once within training data, and the resulting 7-protein panel was fixed at that point. All model objects, random seeds, and a detailed chronological workflow are publicly available at https://github.com/Theda-sys/vasculitis-remission-proteomics. We have now provided a better description of the machine learning model development, feature selection and classifier construction in Methods starting on page 35, line 880.

4. Clinicians do not usually 'rely' on BVAS, BVAS is a tool to record items of vasculitis activity and, as stated in the report, it is subjective, but really summarises the clinician's opinion on the extent of disease activity.

We agree with the reviewer that BVAS is a tool to record items of vasculitis activity, and that the term “rely on BVAS” is a little too strong. We edited the sentence on page 17, line 415, accordingly. It reads now: ”In clinical practice, the BVAS is commonly used to record vasculitis disease activity.”

5. EGPA patients were included in the AAV cohorts. I accept they were MPO-ANCA positive, but they do introduce other pathogenetic pathways and further contribute to heterogeneity.

We agree that EGPA has most likely mechanistic pathway that differ from GPA and MPA. Our study included a small number of EGPA patients, namely 3/39 MPO-AAV patients of the exploratory and 2/55 MPO-AAV patients of the validation cohort. We now mention that these patients likely have common but also distinct features compared to MPA and GPA patients (page 19, line 470).

6. There is no mention of urine biomarkers in the introduction, yet the kidney is the most commonly affected vital organ and urine CD163 has been validated against kidney histology.

We agree with the reviewer that urine biomarker assist in capturing AAV activity in the kidney. Therefore, we have included urine biomarkers in the introduction on page 5, line 98. Moreover, we referenced urine biomarkers (references 45-48) when discussing their role in assessing kidney AAV activity in the discussion. We now explicitly name CD163, MCP-1, and T-cells on page 18, line 436.
